# Photoaddition reactions of *N*-benzylglycinates containing α-trimethylsilyl group with dimethyl acetylenedicarboxylate: competitive formation of pyrroles *vs.* β-enamino esters†

**DOI:** 10.1039/c8ra09996k

**Published:** 2019-02-14

**Authors:** Suk Hyun Lim, Amol B. Atar, Gunoh Bae, Kyung-Ryang Wee, Dae Won Cho

**Affiliations:** Department of Chemistry, Yeungnam University Gyeongsan Gyeongbuk 38541 Republic of Korea dwcho00@yu.ac.kr; Department of Chemistry, Daegu University Gyeongsan Gyeongbuk 38453 Republic of Korea

## Abstract

A study was conducted to gain insight into the preparative potential of photosensitized reactions of acyclic *N*-benzylglycinates containing an α-trimethylsilyl group with dimethyl acetylenedicarboxylate (DMAD). The photosensitizers employed in the reactions include 9,10-dicyanoanthracene (DCA), 1,4-dicyanonaphthalene (DCN), rose bengal (RB) and fullerene C_60_. The results show that photoirradiation of oxygenated solutions containing the photosensitizers, glycinates and dimethyl acetylenedicarboxylate leads to competitive formation of pyrroles and β-enamino-esters. The distributions of pyrrole and β-enamino-ester products formed in these reactions are highly influenced by the electronic nature of the phenyl ring substituent on the benzylglycinates and the photosensitizer used. These photoaddition reactions take place *via* mechanistic pathways involving competitive formation of azomethine ylides and secondary amines, generated by a mechanistic routes involving initial SET from the benzylglycinates to photosensitizers.

## Abstract 2

A study was conducted to gain insight into the preparative potential of photosensitized reactions of acyclic *N*-benzylglycinates containing an α-trimethylsilyl group with dimethyl acetylenedicarboxylate (DMAD).

## Graphical abstract

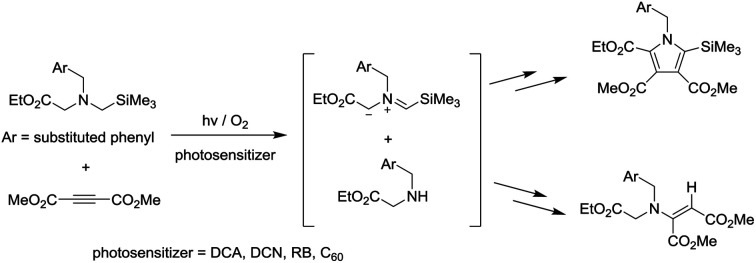

## Introduction

**Scheme 1:**
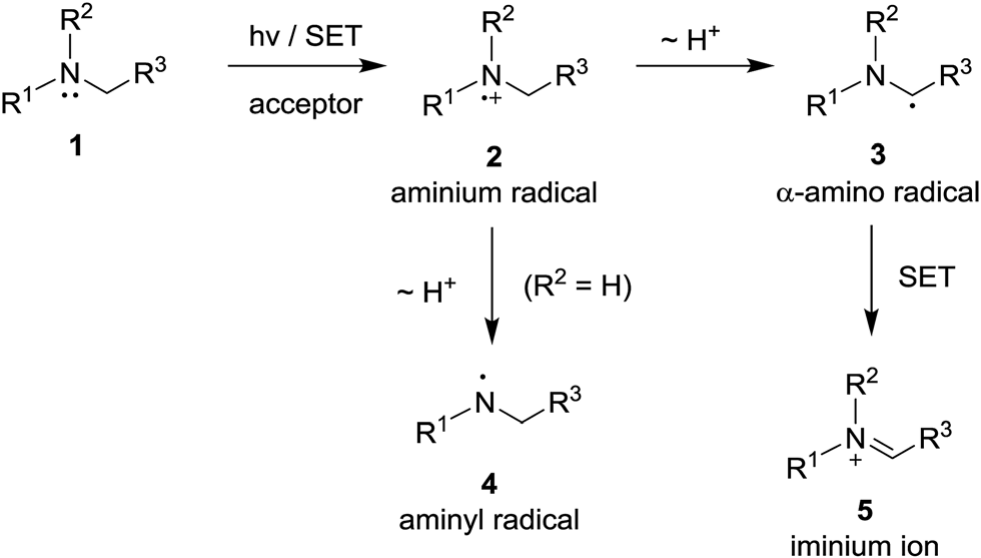
Photoinduced single electron transfer (SET) reaction pathways opened for amine substrates.

Photoinduced single electron transfer (SET), occurring between a variety of electron donating and accepting pairs, is now recognized to be one of the most important events taking place in mechanistically interesting and preparatively useful photochemical reactions.^1–6^ Among a wide variety of electron donating substances that participate in these processes, aliphatic/aromatic amines have perhaps been among the most widely explored.^7–10^ In photoinduced SET reactions of aliphatic/aromatic amines 1, aminium radicals 2 (*i.e.*, amine radical cations) serve as key reactive intermediates (Scheme 1). The most common reaction pathways open to aminium radicals are base-promoted deprotonation from either nitrogen to produce either aminyl radicals 4 (in the cases of primary and secondary aminium radicals)^11,12^ or α-carbon to form α-amino radicals 3 (in the case of tertiary aminium radicals).^7,9*b*,13^ The radical intermediates generated in this manner take part in a variety of addition reactions with electron deficient alkenes, alkynes and unsaturated carbonyl compounds.^8,10*a*,12–15^ In addition, owing to their extremely low oxidation potential,^16^ α-amino radicals 3 undergo ready secondary SET oxidation in the presence of appropriate oxidants to form iminium ions 5.^8*a*,17^

**Scheme 2:**
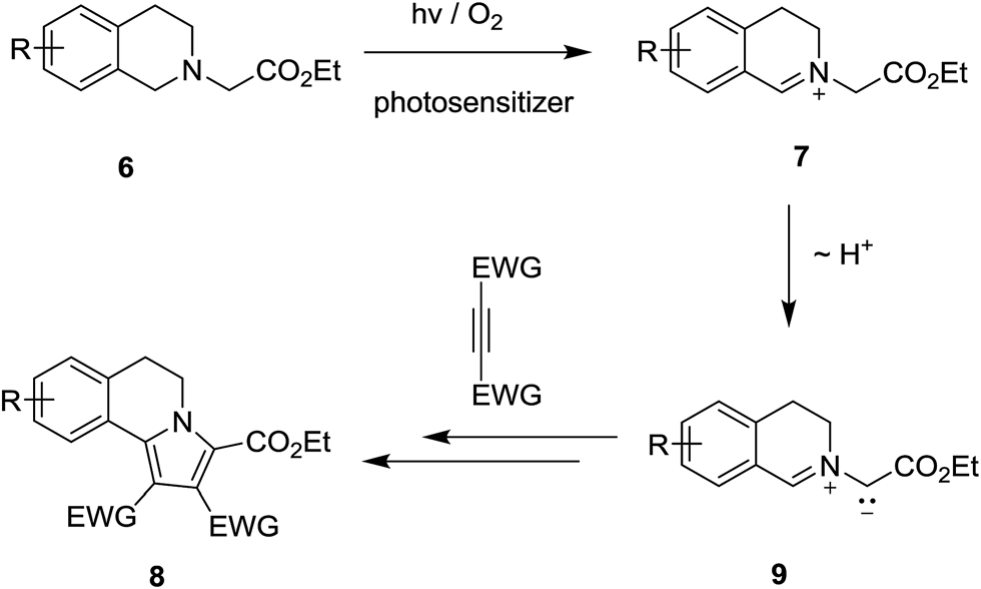
SET-promoted 1,3-dipolar cycloaddition reactions of ethyl 2-(3,4-dihydroisoquinolin-2(1*H*)-yl)acetate 6 with dipolarophile.

Among the interesting photochemical reactions that take place through the sequential SET – α-CH deprotonation pathway are those involving tertiary amines, which possess *N-*carboxymethyl groups (*i.e.*, glycinates).^18^ Tertiary glycinate esters undergo unique cycloaddition reactions with alkenes/alkynes, which take place *via* the intermediacy of 1,3-dipolar azomethine ylides. An interesting example was uncovered in early studies by Xiao^18*a*^ and Rueping,^18*b*^ which showed that photoirradiation of solutions of ethyl 2-(3,4-dihydroisoquinolin-2(1*H*)-yl)acetate derivatives 6 and electron deficient alkenes/alkynes, containing a photosensitizer and molecular oxygen, gives rise to generation of pyrrolo[2,1-*a*]isoquinoline products 8 (Scheme 2). This process likely occurs through a mechanistic pathway entailing deprotonation of an intermediate iminium ion 7 produced by a route (see Scheme 1) that begins with SET from 6 to the electronic excited state of the photosensitizer. The azomethine ylide intermediate 9, generated in this way, undergoes 1,3-dipolar cycloaddition reactions with alkene/alkyne substrates.

**Scheme 3:**
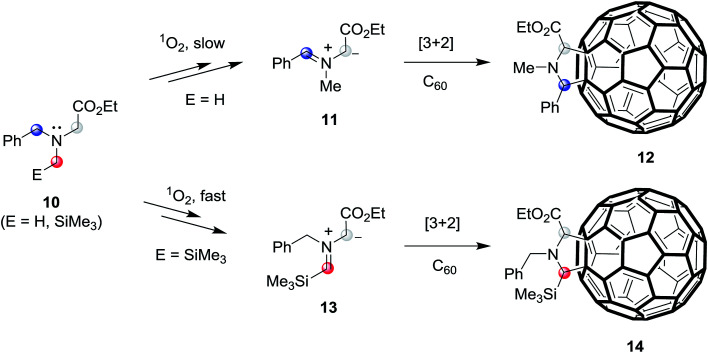
SET-promoted 1,3-dipolar cycloaddition reactions of tertiary *N*-benzylglycinates 10 (E = H, SiMe_3_) with fullerene C_60_.

In recent studies aimed at exploring photoaddition reactions of tertiary amines with fullerene C_60_, we also observed that *N*-benzylglycinates 10 (E = H) could serve as precursors of azomethine ylides (Scheme 3).^10*c*,19^ Specifically, irradiation of solutions of *N*-benzylglycinates 10 (E = H) and C_60_ in the presence of molecular oxygen gives rise to formation of pyrrolidine ring-fused fullerene derivatives 12 (fulleropyrrolidines) (Scheme 3). In this process, C_60_ serve as both the photosensitizer as well as reactive dipolarophile. Moreover, we found that irradiation of oxygenated solutions of C_60_ and *N*-benzylglycinates 10, which possess α-trimethylsilyl groups (E = SiMe_3_), promotes generation of trimethylsilyl group containing fulleropyrrolidines 14 in a much more efficient manner.^19^ These observations suggested that the routes for formation of these products begin with the generation of singlet oxygen (^1^O_2_) *via* energy transfer from the triplet state 
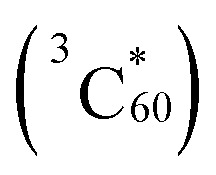
 of the photosensitizer.^20^ Following the suggestion made earlier by Foote and his coworkers,^20*c*^ the formed ^1^O_2_ abstract H-atom from the benzylic position of benzylglycinate 10 (E = H), lacking the α-trimethylsilyl group, followed by SET oxidation to form iminium precursor of azomethine ylides 11, which then cycloadd to C_60_ to form 12. However, in photoreactions of the α-trimethylsilyl group containing *N*-benzylglycinate 10 (E = SiMe_3_), H-atom abstraction mediated by ^1^O_2_ takes place more efficiently at the α-trimethylsilyl substituted carbon position to produce azomethine ylides 13 that cycloadd to C_60_ to generate 14.

Although a variety of photosensitized 1,3-dipolar cyclization reactions of either cyclic *N*-carboxyalkyl substituted tetrahydroisoquinolines^21*a*^ or iminoester derivatives^21*b*^ with dipolarophiles have been mainly performed so far, to the best of knowledge, cyclization reactions utilizing acyclic *N*-carboxyalkyl substituted benzylamine substrates are not common. Thus, the observations made in the investigation described above suggested that azomethine ylides generated from acyclic *N*-benzylglycinates containing α-trimethylsilyl group might participate in photosensitized cycloaddition reactions with a variety of electron deficient dipolarophiles to produce five membered ring *N-*heterocycles. In order to assess this proposal, we explored photosensitized addition reactions of dimethyl acetylenedicarboxylate (DMAD) with *N*-α-trimethylsilyl-*N*-benzylglycinates, possessing a variety of substituents on the arene ring of the benzyl group. The photosensitizers (PS) employed in these processes include 9,10-dicyanoanthracene (DCA), 1,4-dicyanonaphthalene (DCN), rose bengal (RB) and fullerene C_60_. The results arising from this effort show that two competitive pathways are followed in these reactions, one of which involves cycloaddition to form pyrroles and the other generates β-enamino-esters. In addition, the photoproduct distributions (*i.e.*, pyrrole/β-enamine ester ratios) are influenced by the electronic nature of the glycinate substrates and photosensitizer used.

## Results and discussion

**Scheme 4:**
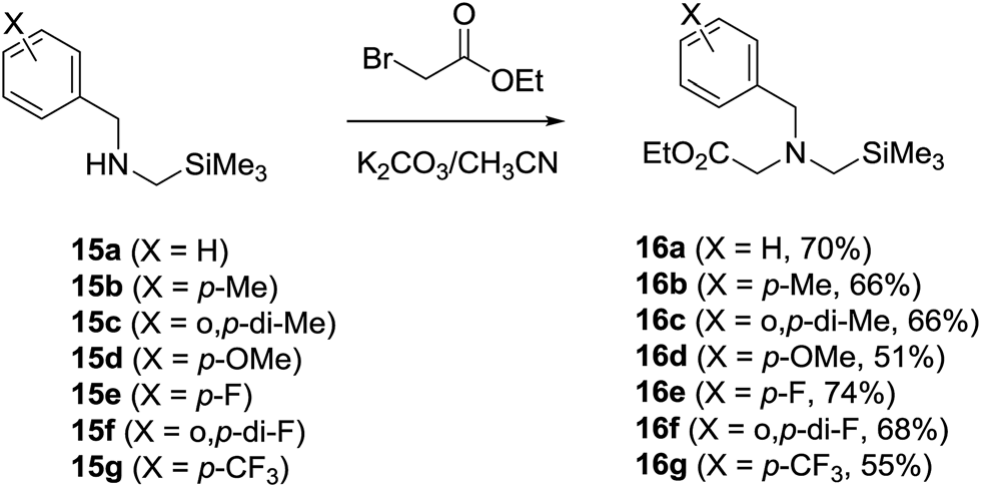
Synthesis of *N*-α-trimethylsilyl-*N*-benzylglycinates 16a–16g.

*N*-α-Trimethylsilyl-*N*-benzylglycinates 16a–16g, in which the benzyl moieties contain various substituents on the phenyl ring, were prepared using an earlier developed synthetic protocol^10*c*,19,22^ (Scheme 4). Photochemical reactions were carried out using O_2_-purged MeCN or toluene solutions containing 16a–16g (3.2 mM) and DMAD (17, 3.2 mM) in the presence of SET photosensitizer. The solutions were irradiated for time periods that bring about 100% conversion of the starting glycinates by using a 450 W Hanovia mercury lamp equipped with a glass filter (*λ* > 310 nm). In all cases, the photolysates were concentrated and the residues were subjected to column chromatography to produce pure samples of the respective photoproducts.

### DCA-promoted photochemical reactions

Prior to carrying out DCA promoted photochemical reactions, oxidation potentials (*E*_ox_) of the glycinate substrates 16a–16g were determined in order to ascertain whether SET from the glycinates to the singlet excited state of the SET photosensitizer DCA (^1^DCA) is thermodynamically favorable. In addition, the rate constants for quenching of the fluorescence of DCA by glycinates 16a–16g in MeCN were determined in order to derive the rate constants (*k*_SET_) for SET. As can be seen viewing the data in Table 1, the glycinates have *E*_ox_ values in the range of 0.83–0.91 V (*vs.* SCE), which are below the reduction potential of singlet state of DCA (^1^DCA) (^1^*E*_red_(DCA) = 1.99 V *vs.* SCE). As a result, free energy changes for SET (Δ*G*_SET_) from all glycinates to ^1^DCA are negative, suggesting that the rates of these processes should be near the diffusion controlled limit. Stern–Volmer analysis of plots of fluorescence intensities of DCA *vs.* glycinate concentrations (Fig. S1, ESI†), showed that the rates of fluorescence quenching by SET from the glycinates 16a–16g to ^1^DCA are near that of diffusion in MeCN.

**Table 1:** Oxidation potentials (*E*_ox_), free energy changes for SET (Δ*G*_SET_) and rate constants for DCA fluorescence quenching by *N*-α-trimethylsilyl-*N*-benzylglycinates 16a–16g in MeCN solutions

Glycinate	*E*_ox_(+) (V *vs.* SCE)	Δ*G*_SET_^a^ (V)	*k*_SET_ × 10^−10^ (M^−1^ s^−1^)
16a	0.84	−1.15	1.1
16b	0.83	−1.16	1.0
16c	0.83	−1.16	—b
16d	0.84	−1.15	—b
16e	0.80	−1.19	0.99
16f	0.89	−1.10	—b
16g	0.91	−1.08	0.86

a Determined by using Δ*G*_SET_ = *E*_ox_ (glycinate) − *E*_red_ (DCA) − *E*_ex_ (DCA), where *E*_red_ (DCA) is −0.91 V (*vs.* SCE) (ref. 23) and *E*_ex_ (DCA) is 2.90 V (ref. 23).

b Not measured.

**Scheme 5:**
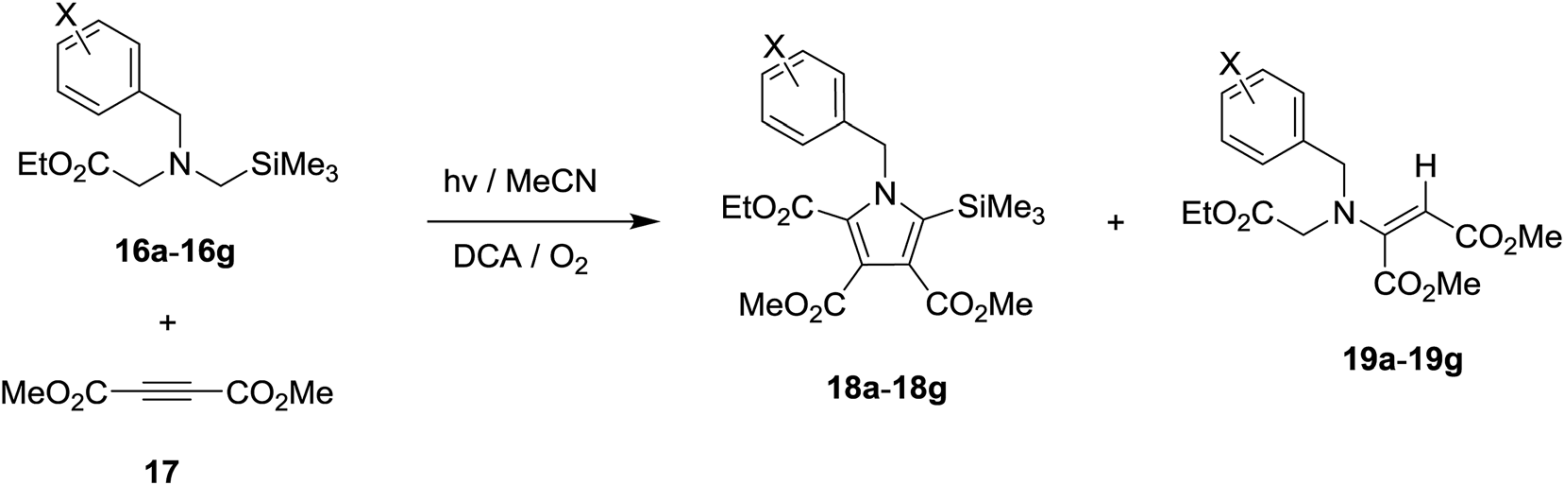
DCA-promoted photochemical reactions of *N*-benzylglycinates 16a–16g with DMAD 17.

Photochemical reactions of *N*-benzylglycinates 16a–16g with DMAD (17) were performed in O_2_-purged MeCN solutions in the presence of DCA for the time periods given in Table 2. Concentration of the photolysates followed by chromatographic separation gave the products shown in Scheme 5 and the yields listed in Table 2. As can be seen by viewing the results, 5 min irradiation of O_2_-purged MeCN solutions containing DCA, acetylene 17 and glycinates 16a–16d, which possess H and electron donating groups (Me, OMe) on the phenyl ring of the benzyl substituent, gave rise to production of the respective β-enamino-esters 19a–19d, along with minor amounts of the corresponding substituted pyrroles 18a–18d (entries 1–4, Table 2). In contrast, 10 min irradiation of solutions containing the mono- and di-fluoro substituted phenyl containing glycinates 16e–16f, 17 and DCA produced *ca.* 1 : 1.5 ratios of pyrroles (18e–18f) and β-enamino-esters (19e–19f) (entries 5 and 6, Table 2). Finally, DCA-promoted photoreaction of the *para*-CF_3_-phenyl containing glycinate 16g with 17 took place to produce both trimethylsilyl-substituted pyrrole 18g (32%) and β-enamino-ester 19g (22%) by 20 min irradiation (entry 7 in Table 2). Importantly, the concentration of DCA in all photoreactions was kept constant and control experiments (entries 8 and 9 in Table 2) revealed that both molecular oxygen and DCA are necessary for reactions to take place.

**Table 2:** Products and yields of DCA-photosensitized reactions of oxygen purged MeCN solutions containing glycinates 16a–16g and acetylene 17^a^

Entry	Amine	X	Irradiation time (min)	Product^b^ (%)
1	16a	H	5	18a (6), 19a (50)
2	16b	*p*-Me	5	18b (4), 19b (51)
3	16c	*o*,*p*-di-Me	5	18c (trace), 19c (48)
4	16d	*p*-OMe	5	18d (trace), 19d (45)
5	16e	*p*-F	10	18e (21), 19e (31)
6	16f	*o*,*p*-di-F	10	18f (24), 19f (33)
7	16g	*p*-CF_3_	20	18g (32), 19g (22)
8^c^	16a	H	300	n.r^d^
9^e^	16a	H	300	n.r^d^

a 220 mL of MeCN solutions containing glycinates 16a–16g (3.2 mM), acetylene 17 (3.2 mM) and DCA (0.27 mM).

b Isolated yields.

c Photoreaction in N_2_-purged MeCN solution.

d No reaction.

e Photoreaction in O_2_-purged MeCN solution without DCA.

### DCN-promoted photochemical reactions

DCN was also utilized as a SET photosensitizer to promote reactions of the glycinates with acetylene 17. Similar to the case of DCA, the calculated free energy changes (Δ*G*_SET_) for SET from the glycinates to the singlet state of DCN (^1^*E*_red_ = 2.3 V *vs.* SCE)^23^ were negative. In addition, the results of fluorescence quenching experiments showed that the glycinates quench the singlet state of DCN (^1^DCN) with near diffusion controlled rates (Fig. S2 of ESI†).

The results of photochemical reactions of *N*-α-trimethylsilyl-*N*-benzylglycinates 16a, 16b, 16e and 16g with acetylene 17 in O_2_-purged MeCN solutions in the presence of DCN are shown in Table 3. As the data show, the DCN-promoted photoreactions took place to produce pyrroles and β-enamino-esters but, in contrast to the DCA-photosensitized reactions, they required much longer irradiation times to bring about complete conversion of glycinate substrates. For example, 60 min irradiation of an oxygenated MeCN solution containing 16a, 17 and DCN gave rise to formation of β-enamino-ester 19a (40%), along with pyrrole 18a (11%) (entry 1 in Table 3). Moreover, 60 min photochemical reaction of the *p*-Me-phenyl substituted glycinate 16b and 17 in the presence of DCN produced β-enamino-ester 19b exclusively (entry 2 in Table 3), and photoreactions of the *p*-F- (16e) and *p*-CF_3_-substituted (16g) glycinates with 17 occurred to produce *ca.* 1 : 3 and 2 : 3 respective ratios of pyrroles (18e and 18g) and the respective enamino-esters (19e and 19g) by much longer irradiation (entries 3 and 4 in Table 3).

**Table 3:** Products and yields of DCN-photosensitized reactions of O_2_-purged MeCN solutions containing glycinates (16a, 16b, 16e and 16g) and acetylene 17^a^

Entry	Glycinate	Irradiation time (min)	Product^b^ (%)
1	16a	60	18a (11), 19a (40)
2	16b	60	18b (4), 19b (49)
3	16e	90	18e (12), 19e (40)
4	16g	180	18g (21), 19g (32)

a 220 mL of MeCN solutions containing glycinates (16a, 16b, 16e and 16g, 3.2 mM), acetylene 17 (3.2 mM) and DCN (0.32 mM).

b Isolated yields.

### RB-promoted photochemical reactions

RB-promoted photoaddition reactions of *N*-α-trimethylsilyl-*N*-benzylglycinates with acetylene 17 were also investigated. Similar to the cases of DCA and DCN, the free energy changes for SET occurring between glycinates and RB (^S1^*E*_red_ = 1.18 V, ^T1^*E*_red_ = 1.02 V *vs.* SCE)^24^ are all negative.

The results displayed in Table 4 show that RB-promoted photochemical reactions O_2_-purged MeCN solutions of glycinates 16a, 16b, 16e and 16g with acetylene 17 take place efficiently to produce pyrroles and β-enamino-esters. Specifically, 5 min irradiation of RB solutions containing 16a–16b and 17 produced a *ca.* 1 : 4 ratios of the corresponding pyrroles 18a–18b (11–12%) and β-enamino-esters 19a–19b (47–49%) (entries 1 and 2 in Table 4). Interestingly, in contrast to those photosensitized by DCA, RB-promoted reactions of the *p*-F- (16e) and *p*-CF_3_- (16g) phenyl substituted glycinates with 17 produced higher yields of the respective β-enamino-esters 19e and 19g (41–45%) than pyrroles 18e and 18g (11–12%) (entries 3 and 4 in Table 4).

**Table 4:** Products and yields of RB-photosensitized reactions of O_2_-purged MeCN solutions containing glycinates (16a, 16b, 16e and 16g) and acetylene 17^a^

Entry	Glycinate	Irradiation time (min)	Product^b^ (%)
1	16a	5	18a (11), 19a (49)
2	16b	5	18b (12), 19b (47)
3	16e	10	18e (12), 19e (45)
4	16g	30	18g (11), 19g (41)

a 220 mL of MeCN solution containing glycinate (3.2 mM), acetylene (3.2 mM) and RB (0.32 mM).

b Isolated yields.

### C_60_-promoted photochemical reactions

A consideration of redox potentials suggests that C_60_ (^3^*E*_red_ = 1.14 V *vs.* SCE)^25^ should serve as a SET-photosensitizer for reactions between the glycinates and DMAD. Owing to the generally low solubility of fullerene C_60_ in MeCN, toluene was used as the solvent for C_60_-promoted photoaddition reactions of *N*-α-trimethylsilyl-*N*-benzylglycinates 16a–16g with acetylene 17. Inspection of the results summarized in Table 5 showed that the product distributions patterns arising from these photochemical reactions are comparable with those from DCA-, DCN- and RB-promoted photochemical reactions of these substrates. Specifically, irradiation of O_2_-purged toluene solutions containing glycinates 16a–16g, 17 and C_60_ produced pyrroles 18a–18g as major photoproducts and β-enamino-esters 19a–19g as minor products (Table 5). Noticeably, no photoproduct arising from addition reactions between glycinates and C_60_ were observed.^19^

**Table 5:** Products and yields of C_60_-photosensitized reactions of O_2_-purged toluene solutions containing glycinates 16a–16g and acetylene 17^a^

Entry	Glycinate	Irradiation time (min)	Product^b^ (%)
1	16a	10	18a (38), 19a (21)
2	16b	10	18b (41), 19b (19)
3	16c	10	18c (38), 19c (16)
4	16d	10	18d (39), 19d (18)
5	16e	20	18e (35), 19e (20)
6	16f	20	18f (32), 19f (21)
7	16g	30	18g (31), 19g (21)

a 220 mL of toluene solution containing glycinate (3.2 mM), acetylene (3.2 mM) and C_60_ (016 mM).

b Isolated yields.

### *N*-trimethylsilyl- and ester-substituent effects on photochemical reactions

In order to gain additional information about how *N*-substituents on the glycinates (*e.g.*, trimethylsilyl and ester groups) influence the efficiencies and product distributions, we probed SET photosensitized reactions of acetylene 17 with the non-trimethylsilyl and non-ester group containing *N*-benzylamine substrates, 20 (ref. 19) and 21,^10*c*^ respectively. As the results depicted in Table 6 show, in the photoreactions of non-trimethylsilyl substituted amine 20 with acetylene 17, mixtures of several types of β-enamino-esters, 19a, 22 and 23,^26^ were produced and no pyrrole products are generated (entries 1–4 in Table 6). In contrast, photoreactions of non-ester group substituted amine 21 with 17 gave rise to β-enamino-ester 23 as a single photoadduct (entries 5–8 in Table 6). Thus, it appears that the presence of both the trimethylsilyl and ester groups in the *N*-benzylamine substrates are essential for these SET-photosensitized reactions to produce pyrrole adducts.

**Table 6:** Products and yields of SET-photosensitized reactions of *N*-benzylamines 20–21 with acetylene 17^a^

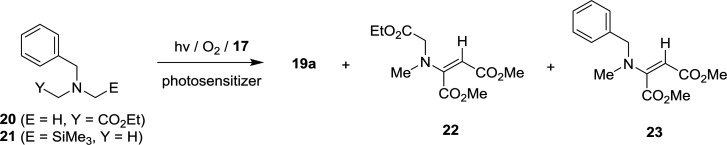				
Entry	Amine	Reaction condition	Irradiation time (min)	Product^b^ (%)
1	20	DCA in MeCN	10	19a (10), 22 (34), 23 (10)
2	20	DCN in MeCN	60	19a (14), 22 (32), 23 (10)
3	20	RB in MeCN	10	19a (17), 22 (29), 23 (11)
4	20	C_60_ in toluene	30	19a (42), 22 (19)
5	21	DCA in MeCN	5	23 (61)
6	21	DCN in MeCN	60	23 (60)
7	21	RB in MeCN	5	23 (58)
8	21	C_60_ in toluene	10	23 (78)

a 220 mL of MeCN or toluene solutions containing *N*-benzylamines (3.2 mM), acetylene (3.2 mM) and photosensitizer (DCA (0.27 mM), DCN (0.32 mM), RB (0.32 mM) and C_60_ (0.16 mM)).

b Isolated yields.

### Mechanistic pathways

**Scheme 6:**
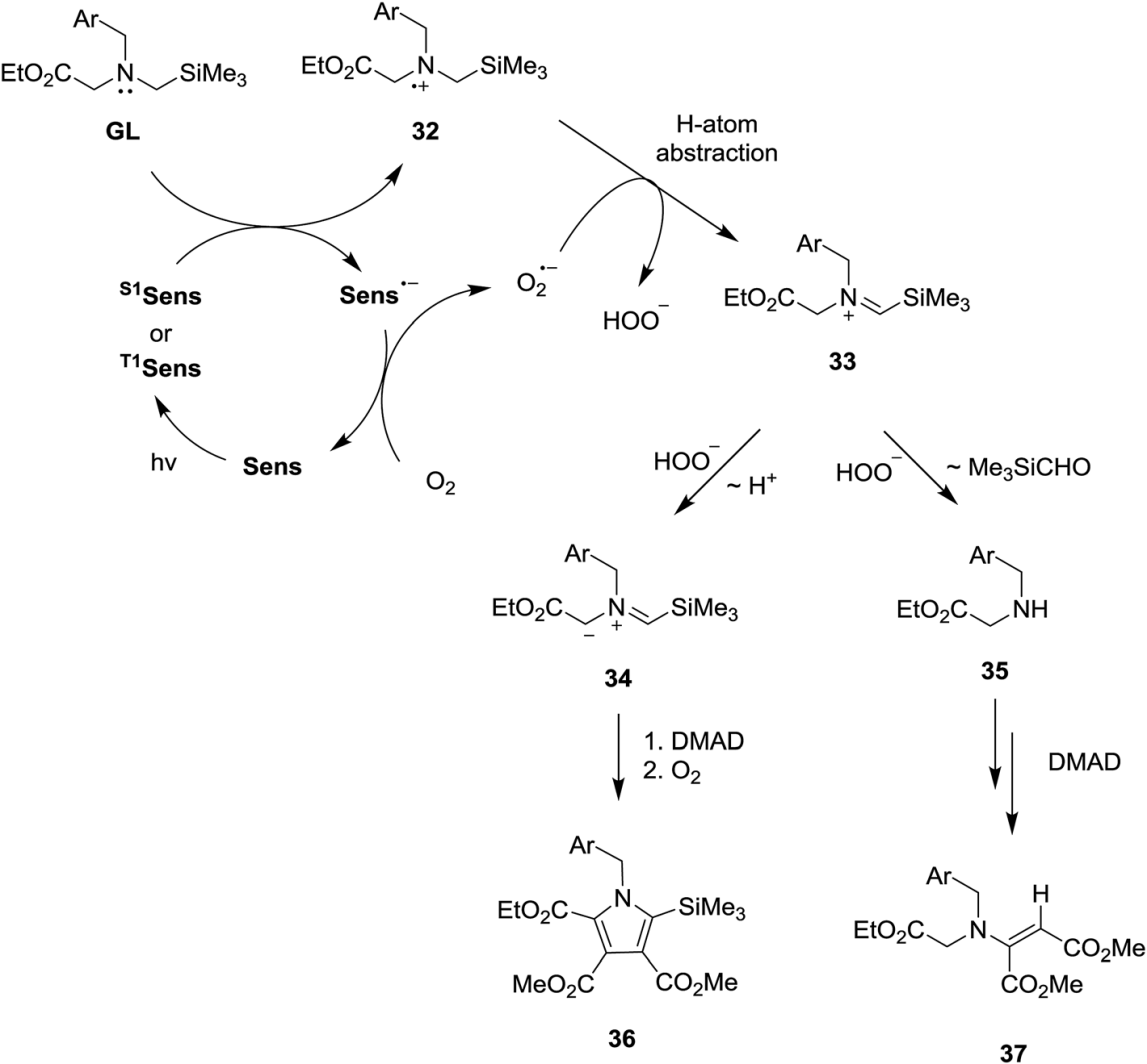
Proposed mechanistic pathways.

The SET-photosensitized reactions described above most likely follow the pathways outlined in Scheme 6, which are terminated either by Michael addition of secondary amines 37 to DMAD to generate β-enamino-esters 40 or by dipolar cycloaddition of azomethine ylides 36 to DMAD to produce precursors of the pyrroles 39. In these processes, the secondary amines (37) and ylides (36) could be formed from the *N*-α-trimethylsilyl-*N*-benzyl glycinates (GL) through a number of different routes. However, it is nearly certain that the β-enamino-ester and pyrrole forming photoreactions are both initiated by SET from the glycinates to the excited states of the sensitizers (^S1 or T1^Sens). This proposal is based on the results of the fluorescence quenching studies described above and a consideration of the concentrations of the glycinates used. Accordingly, the rates of the SET processes (*k*_SET_ [glycinate], where *k*_SET_ = *k*_diff_ = *ca.* 1 × 10^9^ M^−1^ s^−1^ and [glycinate] = 3.2 × 10^−3^ M) should far exceed those for excited state sensitizer decay and energy transfer to molecular oxygen (*k*_ET_ [O_2_], where *k*_ET_ = *ca.* 1.9–7.5 × 10^9^ M^−1^ s^−1^ and [O_2_] ≪ [glycinate]).^20*a*,27^

SET from GL to ^S1 or T1^Sens in the mechanistic pathway generates a glycinate derived aminium radical 32 and a radical anion of the photosensitizer (Sens˙^−^), the former of which serves as a key intermediate in the pathways that produce the pyrrole and β-enamino-ester products. The generated radical anion of the photosensitizer is oxidized by ^3^O_2_ to ground state of photosensitizer (Sens) and O_2_˙^−^ is generated at the same time. While a number of pathways are possible for conversion of 32 to secondary amine 35 and ylide 34, an initial route involving O_2_˙^−^ promoted H-atom abstraction of the aminium radical 32 (leading to 33) is most plausible based on observations made in earlier studies.^18,28^ Then, generated silyl containing iminium ion 33 undergo hydrolytic cleavage by hydrogen peroxide anion (HOO^−^) to form secondary amine 35, which add to DMAD to yield β-amino-ester 37. Competitively, deprotonation of silyl group containing iminium ion 33 produces azomethine ylide 34, which cycloadd to DMAD, followed aromatization, to form pyrrole 36.

**Scheme 7:**
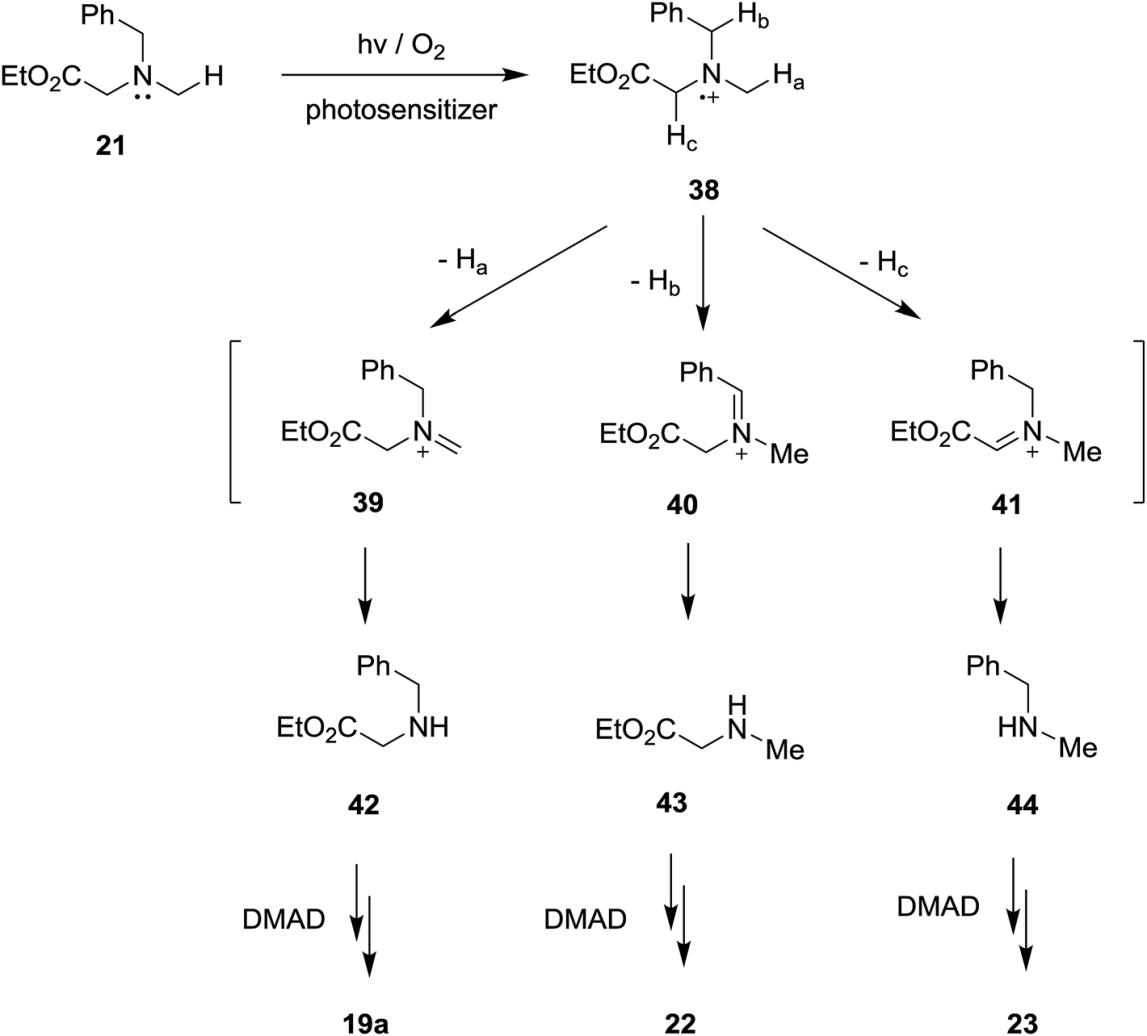
Non-regioselective formation of iminium ions 39–41 from non-silyl amine analog 21.

While conforming to observations made in this study, it is difficult to explain the regioselectivity for H-atom abstraction process from 32 (leading to iminium ion 33) and, in particular, why loss of benzylic or α-ester hydrogen do not take place competitively. However, it is clear that in contrast to non-regioselectivity of aminium radical 38 derived from non-silyl amine analog 21 (Scheme 7), a presence of electrofugal group (*i.e.*, SiMe_3_) in the aminium radicals 32 might lead to regioselective H-atom abstraction process.

## Conclusion

In this study, photoaddition reactions of *N*-α-trimethylsilyl-*N*-benzylglycinates, which contain various kinds of substituents on arene ring, with electron deficient dimethyl acetylenedicarboxylate (DMAD) in the presence of various kinds of SET-photosensitizers were explored in order to assess the preparative utility of trimethylsilyl group containing acyclic *N*-benzylglycinates as substrates in azomethine ylide dipolar cycloaddition reactions.

The results showed that two competitive pathways are followed in these reactions, one of which involves cycloaddition to form pyrroles *via* azomethine ylides and the other generates β-enamino-esters *via* secondary amines. Importantly, the photoproduct distributions (*i.e.*, pyrrole/β-enamine ester ratios) are influenced by the electronic nature of the glycinate substrates and photosensitizer used.

Finally, a plausible mechanistic pathway has been proposed for SET-promoted oxidation reactions of tertiary amines. This proposal along with other interesting features of these reactions will guide the design of future mechanistic and preparative studies.

## Experimental

### General

The ^1^H (300 MHz) and ^13^C NMR (75 MHz) spectra were recorded on CDCl_3_, and chemical shifts were reported in parts per million (*δ*, ppm) relative to CHCl_3_ (7.24 ppm for ^1^H and 77.0 ppm for ^13^C) as an internal standard. High resolution (HRMS) mass spectra were obtained by use of quadrupole mass analyzer and electron impact ionization unless otherwise noted. All new compounds described were isolated as oils unless noted otherwise.

### Synthesis of *N*-α-trimethylsilyl-*N*-benzylglycinates 16a–16g

Individual solutions of *N*-α-trimethylsilyl-*N*-benzylamines 15a–15g (ref. 22*a*) (10 mmol) in acetonitrile (100 mL) containing K_2_CO_3_ (42 mmol) and ethyl bromoacetate (30 mmol) were stirred for 12 h at room temperature and concentrated *in vacuo* to give residues that were triturated with CH_2_Cl_2_. The triturates were dried and concentrated *in vacuo* to afford residues, which were subjected to silica gel column chromatography (EtOAc/hexane = 1 : 5 to 1 : 8) to yield 16a (ref. 10*c*) (70%), 16b (ref. 19) (66%), 16c (66%), 16d (ref. 19) (51%), 16e (ref. 19) (74%), 16f (68%) and 16g (ref. 19) (55%) respectively.

#### 16c (yellow liq)

^1^H-NMR 0.05 (s, 9H), 1.26 (t, 3H, *J* = 6.9 Hz), 2.24 (s, 2H), 2.30 (s, 3H), 2.34 (s, 3H), 3.25 (s, 2H), 3.75 (s, 2H), 4.14 (q, 2H, *J* = 6.9 Hz), 6.95 (d, 1H, *J* = 7.5 Hz), 6.96 (s, 1H), 7.19 (d, 1H, *J* = 7.5 Hz); ^13^C-NMR −1.6, 14.2, 19.0, 20.9, 45.5, 56.4, 59.8, 126.0, 129.8, 130.9, 134.0, 136.4, 137.3, 171.3; HRMS (EI) *m*/*z* 307.1965 (M^+^, C_17_H_29_NO_2_Si requires 307.1968).

#### 16f (yellow liq)

^1^H-NMR 0.01 (s, 9H), 1.22 (t, 3H, *J* = 7.2 Hz), 2.16 (s, 2H), 3.22 (s, 2H), 3.73 (s, 2H), 4.12 (q, 2H, *J* = 7.2 Hz), 6.68–6.75 (m, 1H), 6.76–6.82 (m, 1H), 7.36–7.43 (m, 1H); ^13^C-NMR −1.7, 14.2, 45.6, 53.6, 57.0, 60.1, 103.4 (t, *J* = 25.5 Hz), 110.9 (dd, *J* = 20.8 Hz, 3.8 Hz), 121.9 (dd, *J* = 14.3 Hz, 3.6 Hz), 131.8 (dd, *J* = 9.4 Hz, 6.2 Hz), 161.2 (dd, *J* = 246.9 Hz, 11.8 Hz), 162.0 (dd, *J* = 245.9 Hz, 12 Hz), 171.1; HRMS (EI) *m*/*z* 315.1465 (M^+^, C_15_H_23_F_2_NO_2_Si requires 315.1466).

### General procedure of photoreactions of *N-α*-trimethylsilyl-*N*-benzylglycinates and dimethyl acetylenedicarboxylate (DMAD) in the presence of photosensitizer

Preparative photochemical reactions were conducted using an apparatus consisting of a 450 W Hanovia medium vapor pressure mercury lamp equipped with a flint glass filter (>310 nm) in a water-cooled quartz immersion well surrounded by the solution being irradiated, consisting of solution (220 mL) containing glycinate (0.7 mmol, 3.2 mM), acetylene 17 (0.7 mmol, 3.2 mM), and photocatalyst (DCA (0.27 mM), DCN (0.32 mM), RB (0.32 mM), C_60_ (0.16 mM)). The solution being irradiated was purged with oxygen before and during irradiations for the time periods given below. The photolysates were concentrated *in vacuo* to yield residues, which were subjected to silica gel column chromatography to isolate the pure photoproducts.

### Photoreactions of oxygenated solution of 16a and 17

In MeCN solution of DCA: 5 min irradiation, column chromatography (EtOAc : hexane = 1 : 5) to yield 18a (18 mg, 6%) and 19a (117 mg, 50%). In MeCN solution of DCN: 60 min irradiation, column chromatography to yield 18a (32 mg, 11%) and 19a (94 mg, 40%). In MeCN solution of RB: 5 min irradiation, column chromatography to yield 18a (32 mg, 11%) and 19a (115 mg, 49%). In toluene solution of C_60_: 20 min irradiation, column chromatography to yield 18a (111 mg, 38%) and 19a (49 mg, 21%).

#### 18a (yellow liq)

^1^H-NMR 0.26 (s, 9H), 1.19 (t, 3H, *J* = 7.2 Hz), 3.80 (s, 3H), 3.88 (s, 3H), 4.12 (q, 2H, *J* = 7.2 Hz), 5.77 (s, 2H), 6.78 (d, 2H, *J* = 7.2 Hz), 7.18–7.31 (m, 3H); ^13^C-NMR 1.2, 14.0, 51.5, 51.8, 52.6, 61.1, 122.7, 124.4, 125.2, 126.7, 127.3, 128.8, 138.4, 145.6, 159.6, 164.4, 166.9; HRMS (FAB) *m*/*z* 418.1680 (M + 1, C_21_H_28_NO_6_Si requires 418.1686).

#### 19a (yellow liq)

^1^H-NMR 1.23 (t, 3H, *J* = 6.9 Hz), 3.59 (s, 3H), 3.70 (s, 2H), 3.89 (s, 3H), 4.16 (q, 1H, *J* = 6.9 Hz), 4.38 (s, 2H), 4.70 (s, 1H), 7.22–7.34 (m, 5H); ^13^C-NMR 14.2, 50.2, 51.1, 53.2, 55.2, 61.7, 87.0, 128.1, 128.3, 129.0, 134.9, 154.5, 165.9, 167.9, 168.3; HRMS (FAB) *m*/*z* 336.1445 (M + 1, C_17_H_22_NO_6_ requires 336.1447).

### Photoreactions of oxygenated solution of 16b and 17

In MeCN solution of DCA: 5 min irradiation, column chromatography (EtOAc : hexane = 1 : 5) to yield 18b (12 mg, 4%) and 19b (125 mg, 51%). In MeCN solution of DCN: 60 min irradiation, column chromatography to yield 18b (12 mg, 11%) and 19b (120 mg, 49%). In MeCN solution of RB: 5 min irradiation, column chromatography to yield 18b (36 mg, 12%) and 19b (115 mg, 47%). In toluene solution of C_60_: 10 min irradiation, column chromatography to yield 18b (124 mg, 41%) and 19b (46 mg, 19%).

#### 18b (yellow liq)

^1^H-NMR 0.27 (s, 9H), 1.19 (t, 3H, *J* = 6.9 Hz), 2.28 (s, 3H), 3.79 (s, 3H), 3.87 (s, 3H), 4.12 (q, 2H, *J* = 6.9 Hz), 5.72 (s, 2H), 6.67 (d, 2H, *J* = 7.8 Hz), 7.06 (d, 2H, *J* = 7.8 Hz); ^13^C-NMR 1.1, 13.9, 21.1, 51.3, 51.8, 52.5, 61.0, 122.5, 124.3, 125.0, 126.5, 129.4, 135.3, 136.8, 145.5, 159.5, 164.4, 166.8; HRMS (FAB) *m*/*z* 432.1841 (M + 1, C_22_H_30_NO_6_Si requires 432.1842).

#### 19b (yellow liq)

^1^H-NMR 1.23 (t, 3H, *J* = 7.2 Hz), 2.31 (s, 3H), 3.61 (s, 3H), 3.69 (s, 2H), 3.90 (s, 3H), 4.16 (q, 1H, *J* = 7.2 Hz), 4.34 (s, 2H), 4.70 (s, 1H), 7.13 (s, 4H); ^13^C-NMR 14.3, 21.3, 50.0, 51.1, 53.2, 55.0, 61.7, 86.8, 128.2, 129.7, 131.8, 138.2, 154.5, 165.9, 167.9, 168.4; HRMS (FAB) *m*/*z* 350.1603 (M + 1, C_18_H_24_NO_6_ requires 350.1604).

### Photoreactions of oxygenated solution of 16c and 17

In MeCN solution of DCA: 5 min irradiation, column chromatography (EtOAc : hexane = 1 : 5) to yield 18c (3 mg, 1%) and 19c (121 mg, 48%). In toluene solution of C_60_: 10 min irradiation, column chromatography to yield 18c (120 mg, 38%) and 19c (40 mg, 16%).

#### 18c (yellow liq)

^1^H-NMR 0.21 (s, 9H), 1.18 (t, 3H, *J* = 7.2 Hz), 2.24 (s, 3H), 2.25 (s, 3H), 3.79 (s, 3H), 3.88 (s, 3H), 4.11 (q, 2H, *J* = 7.2 Hz), 5.63 (s, 2H), 5.98 (d, 1H, *J* = 7.8 Hz), 6.82 (d, 1H, *J* = 7.8 Hz), 6.95 (s, 1H); ^13^C-NMR 0.8, 13.8, 18.8, 20.9, 49.9, 51.6, 52.4, 60.9, 122.3, 123.5, 124.0, 126.6, 127.1, 130.8, 133.3, 133.8, 136.5, 145.8, 159.4, 164.2, 166.9; HRMS (EI) *m*/*z* 445.1922 (M^+^, C_23_H_31_NO_6_Si requires 445.1921).

#### 19c (yellow liq)

^1^H-NMR 1.23 (t, 3H, *J* = 6.9 Hz), 2.18 (s, 3H), 2.27 (s, 3H), 3.60 (s, 3H), 3.63 (s, 2H), 3.88 (s, 3H), 4.15 (q, 1H, *J* = 6.9 Hz), 4.33 (s, 2H), 4.76 (s, 1H), 6.94–7.06 (s, 3H); ^13^C-NMR 14.1, 18.8, 20.9, 49.4, 50.9, 52.3, 53.0, 61.4, 87.2, 126.9, 128.6, 129.0, 131.5, 136.7, 138.0, 154.4, 165.7, 167.8, 168.5; HRMS (EI) *m*/*z* 363.1680 (M + 1, C_19_H_25_NO_6_ requires 363.1682).

### Photoreactions of oxygenated solution of 16d and 17

In MeCN solution of DCA: 5 min irradiation, column chromatography (EtOAc : hexane = 1 : 5) to yield 18d (3 mg, 1%) and 19d (115 mg, 45%). In toluene solution of C_60_: 10 min irradiation, column chromatography to yield 18d (123 mg, 39%) and 19d (46 mg, 18%).

#### 18d (yellow liq)

^1^H-NMR 0.27 (s, 9H), 1.19 (t, 3H, *J* = 7.2 Hz), 2.28 (s, 3H), 3.74 (s, 3H), 3.78 (s, 3H), 3.86 (s, 3H), 4.12 (q, 2H, *J* = 7.2 Hz), 5.68 (s, 2H), 6.70 (d, 2H, *J* = 8.7 Hz), 6.78 (d, 2H, *J* = 8.7 Hz); ^13^C-NMR 1.0, 13.8, 50.8, 51.6, 52.3, 55.1, 60.9, 114.0, 122.4, 124.2, 126.2, 126.3, 130.1, 145.2, 158.6, 159.4, 164.2, 166.6; HRMS (EI) *m*/*z* 447.1717 (M + 1, C_22_H_29_NO_7_Si requires 447.1713).

#### 19d (yellow liq)

^1^H-NMR 1.23 (t, 3H, *J* = 7.2 Hz), 3.61 (s, 3H), 3.67 (s, 2H), 3.77 (s, 3H), 3.90 (s, 3H), 4.16 (q, 1H, *J* = 7.2 Hz), 4.31 (s, 2H), 4.70 (s, 1H), 6.84 (d, 2H, *J* = 8.7 Hz), 7.17 (d, 2H, *J* = 8.7 Hz); ^13^C-NMR 13.8, 50.6, 52.7, 54.2, 54.9, 61.2, 86.2, 113.9, 126.3, 128.9, 129.2, 154.1, 159.2, 165.5, 167.5, 167.9; HRMS (EI) *m*/*z* 365.1477 (M + 1, C_18_H_23_NO_7_ requires 365.1475).

### Photoreactions of oxygenated solution of 16e and 17

In MeCN solution of DCA: 10 min irradiation, column chromatography (EtOAc : hexane = 1 : 5) to yield 18e (65 mg, 21%) and 19e (77 mg, 31%). In MeCN solution of DCN: 90 min irradiation, column chromatography to yield 18e (38 mg, 12%) and 19e (99 mg, 40%). In MeCN solution of RB: 10 min irradiation, column chromatography to yield 18e (37 mg, 12%) and 19e (111 mg, 45%). In toluene solution of C_60_: 20 min irradiation, column chromatography to yield 18e (107 mg, 35%) and 19e (49 mg, 20%).

#### 18e (yellow liq)

^1^H-NMR 0.27 (s, 9H), 1.19 (t, 3H, *J* = 7.2 Hz), 3.79 (s, 3H), 3.87 (s, 3H), 4.13 (q, 2H, *J* = 7.2 Hz), 5.72 (s, 2H), 6.73–6.78 (m, 2H), 6.96 (t, 2H, *J* = 8.7 Hz); ^13^C-NMR 1.3, 14.0, 51.0, 51.9, 52.6, 61.2, 115.8 (d, *J* = 21.6 Hz), 122.9, 124.2, 126.9 (d, *J* = 8 Hz), 134.1 (d, *J* = 3.2 Hz), 145.6, 159.6, 161.8 (d, *J* = 244.1 Hz), 164.3, 166.8; HRMS (FAB) *m*/*z* 436.1589 (M + 1, C_21_H_27_FNO_6_Si requires 436.1592).

#### 19e (yellow liq)

^1^H-NMR 1.14 (t, 3H, *J* = 6.9 Hz), 3.50 (s, 3H), 3.64 (s, 2H), 3.80 (s, 3H), 4.07 (q, 1H, *J* = 6.9 Hz), 4.27 (s, 2H), 4.60 (s, 1H), 6.92 (t, 2H, *J* = 8.7 Hz), 7.14–7.18 (m, 2H); ^13^C-NMR 13.8, 49.8, 50.6, 52.7, 54.2, 61.3, 86.8, 116.0 (d, *J* = 21.5 Hz), 129.4 (d, *J* = 8.2 Hz), 130.4 (d, *J* = 3.1 Hz), 153.9, 162.8 (d, *J* = 245.6 Hz), 165.4, 167.3, 168.8; HRMS (FAB) *m*/*z* 354.1355 (M + 1, C_17_H_21_FNO_6_ requires 354.1353).

### Photoreactions of oxygenated solution of 16f and 17

In MeCN solution of DCA: 10 min irradiation, column chromatography (EtOAc : hexane = 1 : 5) to yield 18f (76 mg, 24%) and 19f (86 mg, 33%). In toluene solution of C_60_: 20 min irradiation, column chromatography to yield 18f (102 mg, 32%) and 19f (55 mg, 21%).

#### 18f (yellow liq)

^1^H-NMR 0.25 (s, 9H), 1.20 (t, 3H, *J* = 7.2 Hz), 3.79 (s, 3H), 3.87 (s, 3H), 4.14 (q, 2H, *J* = 7.2 Hz), 5.71 (s, 2H), 6.24–6.32 (m, 1H), 6.70–6.84 (m, 2H); ^13^C-NMR 0.7, 13.7, 46.0, 51.6, 52.4, 61.0, 103.7 (t, *J* = 25.1 Hz), 111.4 (dd, *J* = 21.2 Hz, 3.7 Hz), 121.8 (dd, *J* = 14.6 Hz, 3.8 Hz), 122.7, 123.9, 126.7, 126.8 (dd, *J* = 9.7 Hz, 5.6 Hz), 145.6, 158.8 (dd, *J* = 247.1 Hz, 11.7 Hz), 159.1, 162.0 (dd, *J* = 247.1 Hz, 11.6 Hz), 163.9, 166.4; HRMS (EI) *m*/*z* 453.1421 (M^+^, C_21_H_25_F_2_NO_6_Si requires 453.1419).

#### 19f (yellow liq)

^1^H-NMR 1.21 (t, 3H, *J* = 7.2 Hz), 3.57 (s, 3H), 3.74 (s, 2H), 3.86 (s, 3H), 4.14 (q, 2H, *J* = 7.2 Hz), 4.37 (s, 2H), 4.67 (s, 1H), 6.73–6.86 (m, 2H), 7.24–7.31 (m, 1H); ^13^C-NMR 14.0, 48.2 (d, *J* = 3.8 Hz), 50.8, 50.9, 53.0, 61.6, 87.6, 103.9 (t, *J* = 25.3 Hz), 111.7 (dd, *J* = 21.2 Hz, 3.8 Hz), 117.8 (dd, *J* = 14.3 Hz, 3.7 Hz), 130.65 (dd, *J* = 9.4 Hz, 5.2 Hz), 153.8, 160.7 (dd, *J* = 248.0 Hz, 11.9 Hz), 162.6 (dd, *J* = 248.4 Hz, 11.9 Hz), 165.5, 167.4, 167.9; HRMS (EI) *m*/*z* 371.1182 (M^+^, C_17_H_19_F_2_NO_6_ requires 371.1180).

### Photoreactions of oxygenated solution of 16g and 17

In MeCN solution of DCA: 20 min irradiation, column chromatography (EtOAc : hexane = 1 : 5) to yield 18g (108 mg, 32%), 19g (64 mg, 22%). In MeCN solution of DCN: 180 min irradiation, column chromatography to yield 18g (71 mg, 21%) and 19g (90 mg, 32%). In MeCN solution of RB: 30 min irradiation, column chromatography to yield 18g (36 mg, 11%) and 19g (115 mg, 41%). In toluene solution of C_60_: 30 min irradiation, column chromatography to yield 18g (105 mg, 31%) and 19g (59 mg, 21%).

#### 18g (yellow liq)

^1^H-NMR 0.26 (s, 9H), 1.18 (t, 3H, *J* = 7.2 Hz), 3.80 (s, 3H), 3.87 (s, 3H), 4.12 (q, 2H, *J* = 7.2 Hz), 5.81 (s, 2H), 6.91 (d, *J* = 8.1 Hz), 7.53 (d, *J* = 8.1 Hz); ^13^C-NMR 1.0, 13.7, 51.1, 51.7, 52.4, 61.0, 122.8, 123.8, 125.3, 125.7 (q, *J* = 3.7 Hz), 126.9, 142.3, 145.5, 159.3, 164.0, 166.5; HRMS (EI) *m*/*z* 485.1479 (M^+^, C_22_H_26_F_3_NO_6_Si requires 485.1482).

#### 19g (yellow liq)

^1^H-NMR 1.24 (t, 3H, *J* = 7.2 Hz), 3.61 (s, 3H), 3.74 (s, 2H), 3.89 (s, 3H), 4.18 (q, 2H, *J* = 7.2 Hz), 7.38 (d, 2H, *J* = 8.4 Hz), 7.59 (d, 2H, *J* = 8.4 Hz); ^13^C-NMR 14.0, 50.7, 51.0, 53.1, 54.7, 61.7, 87.8, 125.8 (q, *J* = 3.7 Hz), 127.9, 139.0, 153.9, 165.5, 167.4, 168.0; HRMS (EI) *m*/*z* 403.1244 (M^+^, C_18_H_20_F_3_NO_6_ requires 403.1243).

### Photoreactions of oxygenated solution of 20 and 17

In MeCN solution of DCA: 10 min irradiation, column chromatography (EtOAc : hexane = 1 : 5) to yield 19a (29 mg, 10%), 22 (62 mg, 34%) and 23 (18 mg, 10%). In MeCN solution of DCN: 60 min irradiation, column chromatography to yield 19a (41 mg, 14%), 22 (58 mg, 32%) and 23 (18 mg, 10%). In MeCN solution of RB: 10 min irradiation, column chromatography to yield 19a (50 mg, 17%), 22 (53 mg, 29%) and 23 (20 mg, 11%). In toluene solution of C_60_: 30 min irradiation, column chromatography to yield 19a (123 mg, 42%) and 22 (34 mg, 19%).

#### 22 (yellow liq)

^1^H-NMR 1.25 (t, 3H, *J* = 7.2 Hz), 2.91 (s, 3H), 3.60 (s, 3H), 3.81 (s, 2H), 3.87 (s, 3H), 4.18 (q, 2H, *J* = 7.2 Hz), 4.65 (s, 1H); ^13^C-NMR 14.1, 39.2, 50.9, 53.0, 54.0, 61.6, 86.9, 154.3, 165.7, 167.7, 168.4; HRMS (EI) *m*/*z* 259.1052 (M^+^, C_11_H_17_NO_6_ requires 259.1056).

#### 23 (yellow liq)

^1^H-NMR 2.72 (s, 3H), 3.61 (s, 3H), 3.90 (s, 3H), 4.27 (s, 2H), 4.65 (s, 1H), 7.19–7.34 (m, 5H); ^13^C-NMR 36.8, 50.7, 52.9, 56.3, 84.6, 127.3, 127.8, 128.7, 135.5, 154.9, 166.0, 168.0; HRMS (EI) *m*/*z* 263.1154 (M^+^, C_14_H_17_NO_4_ requires 263.1158).

### Photoreactions of oxygenated solution of 21 and 17

In MeCN solution of DCA: 5 min irradiation, column chromatography (EtOAc : hexane = 1 : 5) to yield 23 (112 mg, 61%). In MeCN solution of DCN: 60 min irradiation, column chromatography to yield 23 (111 mg, 60%). In MeCN solution of RB: 5 min irradiation, column chromatography to yield 23 (107 mg, 58%). In toluene solution of C_60_: 10 min irradiation, column chromatography to yield 23 (144 mg, 78%).

## Conflicts of interest

There are no conflict of interest to declare.

## Supplementary Material

RA-009-C8RA09996K-s001
