# Supplementary material for: Photoaddition reactions of N-benzylglycinates containing α-trimethylsilyl group with dimethyl acetylenedicarboxylate: competitive formation of pyrroles vs. β-enamino esters
Source: RSC Adv. 2019 Feb 14;9(10):5639–48. doi: 10.1039/c8ra09996k (PMC9060770; doi:10.1039/c8ra09996k)
Supplement: RA-009-C8RA09996K-s001 [file RA-009-C8RA09996K-s001.pdf]

Electronic Supplementary Material (ESI) for RSC Advances.
This journal is © The Royal Society of Chemistry 2019

Supplementary Information

Photoaddition Reactions of *N*-Benzylglycinates Containing α-Trimethylsilyl Group with Dimethyl Acetylenedicarboxylate: Competitive Formation of Pyrroles vs β-Enamino Esters

Suk Hyun Lim,[1] Amol B. Atar,[1] Gunoh Bae,[2] Kyung-Ryang Wee,[2] Dae Won Cho[1*]

[1]*Department of Chemistry, Yeungnam University, Gyeongsan, Gyeongbuk 38541, Republic of Korea (dwcho00@yu.ac.kr)*

[2]*Department of Chemistry, Daegu University, Gyeongsan, Gyeongbuk 38453, Republic of Korea*

Table of Contents

Experimental --- S2 - S10

DCA fluorescence quenching experiment -- S11 - S12

DCN fluorescence quenching experiment -- S13 - S14

^{1}H- and ^{13}C NMR spectra of unknown Compounds ------------------------------------ S15 - S50

Experimental

^{1}H- and ^{13}C-NMR (300 *MHz*) spectra were recorded on $CDCl_3$ solutions and chemical shifts were reported in parts per million relative to $CHCl_3$ peak (7.24 ppm for ^{1}H-NMR and 77.0 ppm for ^{13}C-NMR) as an internal standard. High resolution (HRMS) mass spectra were obtained by use of quadrupole mass analyzer and electron impact ionization unless otherwise noted. All new compounds described were isolated as oils unless noted otherwise.

Synthesis of *N*-α-trimethylsilyl-*N*-benzylglycinates 16a-16g. Individual solutions of *N*-α-trimethylsilyl-*N*-benzylamines **15a-15g**[1] (10 mmol) in acetonitrile (100 mL) containing K_2CO_3 (42 mmol) and ethyl bromoacetate (30 mmol) were stirred for 12 h at room temperature and concentrated in vacuo to give residues that were triturated with CH_2Cl_2. The triturates were dried and concentrated in vacuo to afford residues, which were subjected to silica gel column chromatography (EtOAc/hexane = 1:5 to 1: 8) to yield **16a**[2] (70%), **16b**[3] (66%), **16c** (66%), **16d**[3] (51%), **16e**[3] (74%), **16f** (68%) and **16g**[3] (55%) respectively.

16c: ^{1}H-NMR 0.05 (s, 9H), 1.26 (t, 3H, *J* = 6.9 Hz), 2.24 (s, 2H), 2.30 (s, 3H), 2.34 (s, 3H), 3.25 (s, 2H), 3.75 (s, 2H), 4.14 (q, 2H, *J* = 6.9 Hz), 6.95 (d, 1H, *J* = 7.5 Hz), 6.96 (s, 1H), 7.19 (d, 1H, *J* = 7.5 Hz); ^{13}C-NMR -1.6, 14.2, 19.0, 20.9, 45.5, 56.4, 59.8, 126.0, 129.8, 130.9, 134.0, 136.4, 137.3, 171.3; HRMS (EI) *m/z* 307.1965 (M^+, $C_{17}H_{29}NO_2Si$ requires 307.1968).

16f: ^{1}H-NMR 0.01 (s, 9H), 1.22 (t, 3H, *J* = 7.2 Hz), 2.16 (s, 2H), 3.22 (s, 2H), 3.73 (s, 2H), 4.12 (q, 2H, *J* = 7.2 Hz), 6.68-6.75 (m, 1H), 6.76-6.82 (m, 1H), 7.36-7.43 (m, 1H); ^{13}C-NMR -1.7, 14.2,

45.6, 53.6, 57.0, 60.1, 103.4 (t, *J* = 102 Hz), 110.9 (dd, *J* = 83 Hz, 15 Hz), 121.9 (dd, *J* = 57 Hz, 14.4 Hz), 131.8 (dd, *J* = 37.7 Hz, 24.9 Hz), 161.2 (dd, *J* = 989.7 Hz, 47 Hz), 162.0 (dd, *J* = 983.6 Hz, 48 Hz), 171.1; HRMS (EI) *m/z* 315.1465 (M^+, $C_{15}H_{23}F_2NO_2Si$ requires 315.1466).

General procedure of photoreactions of *N*-α-trimethylsilyl-*N*-benzylglycinates and dimethyl acetylenedicarboxylate (DMAD) in the presence of photosensitizer. Preparative photochemical reactions were conducted using an apparatus consisting of a 450 W Hanovia medium vapor pressure mercury lamp equipped with a flint glass filter (>310 nm) in a water-cooled quartz immersion well surrounded by the solution being irradiated, consisting of solution (220 mL) containing glycinate (0.7 mmol, 3.2 mM), acethylene **17** (0.7 mmol, 3.2 mM), and photocatalyst (DCA (0.27 mM), DCN (0.32 mM), RB (0.32 mM), C_{60} (0.16 mM)). The solution being irradiated was purged with oxygen before and during irradiations for the time periods given below. The photolysates were concentrated in vacuo to yield residues, which were subjected to silica gel column chromatography to isolate the pure photoproducts.

Photoreactions of oxygenated solution of 16a and 17. *In MeCN solution of DCA.* 5 min irradiation, column chromatography (EtOAc: hexane = 1: 5) to yield **18a** (18 mg, 6%) and **19a** (117 mg, 50%). *In MeCN solution of DCN.* 60 min irradiation, column chromatography to yield **18a** (32 mg, 11%) and **19a** (96 mg, 41%). *In MeCN solution of RB.* 5 min irradiation, column chromatography to yield **18a** (32 mg, 11%) and **19a** (115 mg, 49%). *In toluene solution of C_{60}.* 20 min irradiation, column chromatography to yield **18a** (111 mg, 38%) and **19a** (49 mg, 21%).

18a: ^{1}H-NMR 0.26 (s, 9H), 1.19 (t, 3H, *J* = 7.2 Hz), 3.80 (s, 3H), 3.88 (s, 3H), 4.12 (q, 2H, *J* = 7.2 Hz), 5.77 (s, 2H), 6.78 (d, 2H, *J* = 7.2 Hz), 7.18-7.31 (m, 3H); ^{13}C-NMR 1.2, 14.0, 51.5, 51.8, 52.6, 61.1, 122.7, 124.4, 125.2, 126.7, 127.3, 128.8, 138.4, 145.6, 159.6, 164.4, 166.9; HRMS (FAB) *m/z* 418.1680 (M+1, $C_{21}H_{28}NO_6Si$ requires 418.1686).

19a: ^{1}H-NMR 1.23 (t, 3H, *J* = 6.9 Hz), 3.59 (s, 3H), 3.70 (s, 2H), 3.89 (s, 3H), 4.16 (q, 1H, *J* = 6.9 Hz), 4.38 (s, 2H), 4.70 (s, 1H), 7.22-7.34 (m, 5H); ^{13}C-NMR 14.2, 50.2, 51.1, 53.2, 55.2, 61.7, 87.0, 128.1, 128.3, 129.0, 134.9, 154.5, 165.9, 167.9, 168.3; HRMS (FAB) *m/z* 336.1445 (M+1, $C_{17}H_{22}NO_6$ requires 336.1447).

Photoreactions of oxygenated solution of 16b and 17. *In MeCN solution of DCA.* 5 min irradiation, column chromatography (EtOAc: hexane = 1: 5) to yield **18b** (12 mg, 4%) and **19b** (125 mg, 51%). *In MeCN solution of DCN.* 60 min irradiation, column chromatography to yield **18b** (12 mg, 11%) and **19b** (120 mg, 49%). *In MeCN solution of RB.* 5 min irradiation, column chromatography to yield **18b** (36 mg, 12%) and **19b** (115 mg, 47%). *In toluene solution of C_{60}.* 10 min irradiation, column chromatography to yield **18b** (124 mg, 41%) and **19b** (46 mg, 19%).

18b: ^{1}H-NMR 0.27 (s, 9H), 1.19 (t, 3H, *J* = 6.9 Hz), 2.28 (s, 3H), 3.79 (s, 3H), 3.87 (s, 3H), 4.12 (q, 2H, *J* = 6.9 Hz), 5.72 (s, 2H), 6.67 (d, 2H, *J* = 7.8 Hz), 7.06 (d, 2H, *J* = 7.8 Hz); ^{13}C-NMR 1.1, 13.9, 21.1, 51.3, 51.8, 52.5, 61.0, 122.5, 124.3, 125.0, 126.5, 129.4, 135.3, 136.8, 145.5, 159.5, 164.4, 166.8; HRMS (FAB) *m/z* 432.1841 (M+1, $C_{22}H_{30}NO_6Si$ requires 432.1842).

19b: ^{1}H-NMR 1.23 (t, 3H, *J* = 7.2 Hz), 2.31 (s, 3H), 3.61 (s, 3H), 3.69 (s, 2H), 3.90 (s, 3H), 4.16 (q, 1H, *J* = 7.2 Hz), 4.34 (s, 2H), 4.70 (s, 1H), 7.13 (s, 4H); ^{13}C-NMR 14.3, 21.3, 50.0, 51.1, 53.2, 55.0, 61.7, 86.8, 128.2, 129.7, 131.8, 138.2, 154.5, 165.9, 167.9, 168.4; HRMS (FAB) *m/z* 350.1603 (M+1, $C_{18}H_{24}NO_6$ requires 350.1604).

Photoreactions of oxygenated solution of 16c and 17. *In MeCN solution of DCA.* 5 min irradiation, column chromatography (EtOAc: hexane = 1: 5) to yield **18c** (3 mg, 1%) and **19c** (127 mg, 50%). *In toluene solution of C_{60}.* 10 min irradiation, column chromatography to yield **18c** (122 mg, 39%) and **19c** (38 mg, 15%).

18c: ^{1}H-NMR 0.21 (s, 9H), 1.18 (t, 3H, *J* = 7.2 Hz), 2.24 (s, 3H), 2.25 (s, 3H), 3.79 (s, 3H), 3.88 (s, 3H), 4.11 (q, 2H, *J* = 7.2 Hz), 5.63 (s, 2H), 5.98 (d, 1H, *J* = 7.8 Hz), 6.82 (d, 1H, *J* = 7.8 Hz), 6.95 (s, 1H) ; ^{13}C-NMR 0.8, 13.8, 18.8, 20.9, 49.9, 51.6, 52.4, 60.9, 122.3, 123.5, 124.0, 126.6, 127.1, 130.8, 133.3, 133.8, 136.5, 145.8, 159.4, 164.2, 166.9; HRMS (EI) *m/z* 445.1922 (M^+, $C_{23}H_{31}NO_6Si$ requires 445.1921).

19c: ^{1}H-NMR 1.23 (t, 3H, *J* = 6.9 Hz), 2.18 (s, 3H), 2.27 (s, 3H), 3.60 (s, 3H), 3.63 (s, 2H), 3.88 (s, 3H), 4.15 (q, 1H, *J* = 6.9 Hz), 4.33 (s, 2H), 4.76 (s, 1H), 6.94-7.06 (s, 3H); ^{13}C-NMR 14.1, 18.8, 20.9, 49.4, 50.9, 52.3, 52.9, 61.4, 87.2, 126.9, 128.6, 129.0, 131.5, 136.7, 138.0, 154.4, 165.7, 167.7, 168.5; HRMS (EI) *m/z* 363.1680 (M+1, $C_{19}H_{25}NO_6$ requires 363.1682).

Photoreactions of oxygenated solution of 16d and 17. *In MeCN solution of DCA.* 5 min irradiation, column chromatography (EtOAc: hexane = 1: 5) to yield **18d** (3 mg, 1%) and **19d** (118 mg,

46%). *In toluene solution of C_{60}.* 10 min irradiation, column chromatography to yield **18d** (125 mg, 40%) and **19d** (46 mg, 18%).

18d: ^{1}H-NMR 0.27 (s, 9H), 1.19 (t, 3H, *J* = 7.2 Hz), 2.28 (s, 3H), 3.74 (s, 3H), 3.78 (s, 3H), 3.86 (s, 3H), 4.12 (q, 2H, *J* = 7.2 Hz), 5.68 (s, 2H), 6.70 (d, 2H, *J* = 8.7 Hz), 6.78 (d, 2H, *J* = 8.7 Hz); ^{13}C-NMR 1.0, 13.8, 50.8, 51.6, 52.3, 55.1, 60.9, 114.0, 122.4, 124.2, 126.2, 126.3, 130.1, 145.2, 158.6, 159.4, 164.2, 166.6; HRMS (EI) *m/z* 447.1717 (M+1, $C_{22}H_{29}NO_7Si$ requires 447.1713).

19d: ^{1}H-NMR 1.23 (t, 3H, *J* = 7.2 Hz), 3.61 (s, 3H), 3.67 (s, 2H), 3.77 (s, 3H), 3.90 (s, 3H), 4.16 (q, 1H, *J* = 7.2 Hz), 4.31 (s, 2H), 4.70 (s, 1H), 6.84 (d, 2H, *J* = 8.7 Hz), 7.17 (d, 2H, *J* = 8.7 Hz); ^{13}C-NMR 14.1, 49.6, 51.0, 53.1, 54.5, 55.3, 61.5, 86.7, 114.2, 126.6, 129.5, 154.3, 159.5, 165.8, 167.8, 168.3; HRMS (EI) *m/z* 365.1477 (M+1, $C_{18}H_{23}NO_7$ requires 365.1475).

Photoreactions of oxygenated solution of 16e and 17. *In MeCN solution of DCA.* 10 min irradiation, column chromatography (EtOAc: hexane = 1: 5) to yield **18e** (67 mg, 22%) and **19e** (79 mg, 32%). *In MeCN solution of DCN.* 90 min irradiation, column chromatography to yield **18e** (40 mg, 13%) and **19e** (99 mg, 40%). *In MeCN solution of RB.* 10 min irradiation, column chromatography to yield **18e** (37 mg, 12%) and **19e** (111 mg, 45%). *In toluene solution of C_{60}.* 20 min irradiation, column chromatography to yield **18e** (110 mg, 36%) and **19e** (47 mg, 19%).

18e: ^{1}H-NMR 0.27 (s, 9H), 1.19 (t, 3H, *J* = 7.2 Hz), 3.79 (s, 3H), 3.87 (s, 3H), 4.13 (q, 2H, *J* = 7.2 Hz), 5.72 (s, 2H), 6.73-6.78 (m, 2H), 6.96 (t, 2H, *J* = 8.7 Hz); ^{13}C-NMR 1.3, 14.0, 51.0, 51.9, 52.6, 61.2, 115.8 (d, *J* = 86.4 Hz), 122.9, 124.2, 126.9 (d, *J* = 32.1 Hz), 134.1 (d, *J* = 12.6 Hz), 145.6, 159.6,

161.8 (d, *J* = 976.2 Hz), 164.3, 166.8; HRMS (FAB) *m/z* 436.1589 (M+1, $C_{21}H_{27}FNO_6Si$ requires 436.1592).

19e: ^{1}H-NMR 1.23 (t, 3H, *J* = 6.9 Hz), 3.60 (s, 3H), 3.69 (s, 2H), 3.89 (s, 3H), 4.16 (q, 1H, *J* = 6.9 Hz), 4.34 (s, 2H), 4.69 (s, 1H), 7.00 (t, 2H, *J* = 8.7 Hz), 7.20-7.24 (m, 2H); ^{13}C-NMR 14.3, 50.3, 51.2, 53.3, 54.7, 61.9, 87.4, 116.0 (d, *J* = 85.8 Hz), 130.0 (d, *J* = 32.7 Hz), 130.8 (d, *J* = 12.3 Hz), 154.3, 162.8 (d, *J* = 982.2 Hz), 166.0, 167.9, 168.3; HRMS (FAB) *m/z* 354.1355 (M+1, $C_{17}H_{21}FNO_6$ requires 354.1353).

Photoreactions of oxygenated solution of 16f and 17. *In MeCN solution of DCA.* 10 min irradiation, column chromatography (EtOAc: hexane = 1: 5) to yield **18f** (76 mg, 24%) and **19f** (88 mg, 34%). *In toluene solution of* C_{60}. 20 min irradiation, column chromatography to yield **18f** (102 mg, 32%) and **19f** (55 mg, 21%).

18f: ^{1}H-NMR 0.25 (s, 9H), 1.20 (t, 3H, *J* = 7.2 Hz), 3.79 (s, 3H), 3.87 (s, 3H), 4.14 (q, 2H, *J* = 7.2 Hz), 5.71 (s, 2H), 6.24-6.32 (m, 1H), 6.70-6.84 (m, 2H); ^{13}C-NMR 0.7, 13.7, 46.0, 51.6, 52.4, 61.0, 103.7 (t, *J* = 100.2 Hz), 111.4 (dd, *J* = 84.8 Hz, 14.7 Hz), 121.8 (dd, *J* = 58.4 Hz, 15 Hz), 122.7, 123.9, 126.7, 126.8 (dd, *J* = 38.6 Hz, 22.2 Hz), 145.6, 158.8 (dd, *J* = 988.5 Hz, 46.8 Hz), 159.1, 162.0 (dd, *J* = 988.4 Hz, 46.2 Hz), 163.9, 166.4; HRMS (EI) *m/z* 453.1421 (M^+, $C_{21}H_{25}F_2NO_6Si$ requires 453.1419).

19f: ^{1}H-NMR 1.21 (t, 3H, *J* = 7.2 Hz), 3.57 (s, 3H), 3.74 (s, 2H), 3.86 (s, 3H), 4.14 (q, 2H, *J* = 7.2 Hz), 4.37 (s, 2H), 4.67 (s, 1H), 6.73-6.86 (m, 2H), 7.24-7.31 (m, 1H); ^{13}C-NMR 14.0, 48.2 (d, J = 15.3 Hz), 50.8, 50.9, 53.0, 61.6, 87.6, 103.9 (t, *J* = 101.1 Hz), 111.7 (dd, *J* = 84.9 Hz, 15 Hz), 117.8

(dd, *J* = 57.2 Hz, 14.7 Hz), 130.65 (dd, *J* = 37.5 Hz, 20.7 Hz), 153.8, 160.7 (dd, *J* = 992.1 Hz, 47.7 Hz), 162.6 (dd, *J* = 993.5 Hz, 47.4 Hz), 165.5, 167.4, 167.9; HRMS (EI) *m/z* 371.1182 (M^+, $C_{17}H_{19}F_2NO_6$ requires 371.1180).

Photoreactions of oxygenated solution of 16g and 17. *In MeCN solution of DCA.* 20 min irradiation, column chromatography (EtOAc: hexane = 1: 5) to yield **18g** (105 mg, 31%), **19g** (66 mg, 23%). *In MeCN solution of DCN.* 180 min irradiation, column chromatography to yield **18g** (71 mg, 21%) and **19g** (93 mg, 33%). *In MeCN solution of RB.* 30 min irradiation, column chromatography to yield **18g** (34 mg, 10%) and **19g** (121 mg, 43%). *In toluene solution of* C_{60}. 30 min irradiation, column chromatography to yield **18g** (105 mg, 31%) and **19g** (59 mg, 21%).

18g: ^{1}H-NMR 0.26 (s, 9H), 1.18 (t, 3H, *J* = 7.2 Hz), 3.80 (s, 3H), 3.87 (s, 3H), 4.12 (q, 2H, *J* = 7.2 Hz), 5.81 (s, 2H), 6.91 (d, *J* = 8.1 Hz), 7.53 (d, *J* = 8.1 Hz); ^{13}C-NMR 1.0, 13.7, 51.1, 51.7, 52.4, 61.0, 122.8, 123.8, 125.3, 125.7 (q, *J* = 14.7 Hz), 126.9, 142.3, 145.5, 159.3, 164.0, 166.5; HRMS (EI) *m/z* 485.1479 (M^+, $C_{22}H_{26}F_3NO_6Si$ requires 485.1482).

19g: ^{1}H-NMR 1.24 (t, 3H, *J* = 7.2 Hz), 3.61 (s, 3H), 3.74 (s, 2H), 3.89 (s, 3H), 4.18 (q, 2H, *J* = 7.2 Hz), 7.38 (d, 2H, *J* = 8.4 Hz), 7.59 (d, 2H, *J* = 8.4 Hz); ^{13}C-NMR 14.0, 50.7, 51.0, 53.1, 54.7, 61.7, 87.8, 125.8 (q, *J* = 14.7 Hz), 127.9, 139.0, 153.9, 165.5, 167.4, 168.0; HRMS (EI) *m/z* 403.1244 (M^+, $C_{18}H_{20}F_3NO_6$ requires 403.1243).

Photoreactions of oxygenated solution of 20 and 17. *In MeCN solution of DCA.* 10 min irradiation, column chromatography (EtOAc: hexane = 1: 5) to yield **19a** (29 mg, 10%), **22** (62 mg,

34%) and **23** (18 mg, 10%). *In MeCN solution of DCN.* 60 min irradiation, column chromatography to yield **19a** (41 mg, 14%), **22** (58 mg, 32%) and **23** (18 mg, 10%). *In MeCN solution of RB.* 10 min irradiation, column chromatography to yield **19a** (50 mg, 17%), **22** (53 mg, 29%) and **23** (20 mg, 11%). *In toluene solution of C_{60}.* 30 min irradiation, column chromatography to yield **19a** (123 mg, 42%) and **22** (34 mg, 19%).

22: ^{1}H-NMR 1.25 (t, 3H, *J* = 7.2 Hz), 2.91 (s, 3H), 3.60 (s, 3H), 3.81 (s, 2H), 3.87 (s, 3H), 4.18 (q, 2H, *J* = 7.2 Hz), 4.65 (s, 1H); ^{13}C-NMR 14.1, 39.2, 50.9, 53.0, 54.0, 61.6, 86.9, 154.3, 165.7, 167.7, 168.4; HRMS (EI) *m/z* 259.1052 (M^+, $C_{11}H_{17}NO_6$ requires 259.1056).

23: ^{1}H-NMR 2.72 (s, 3H), 3.61 (s, 3H), 3.90 (s, 3H), 4.27 (s, 2H), 4.65 (s, 1H), 7.19-7.34 (m, 5H); ^{13}C-NMR 36.8, 50.7, 52.9, 56.3, 84.6, 127.3, 127.8, 128.7, 135.5, 154.9, 166.0, 168.0; HRMS (EI) *m/z* 263.1154 (M^+, $C_{14}H_{17}NO_4$ requires 263.1158).

Photoreactions of oxygenated solution of 21 and 17. *In MeCN solution of DCA.* 5 min irradiation, column chromatography (EtOAc: hexane = 1: 5) to yield **23** (112 mg, 61%). *In MeCN solution of DCN.* 60 min irradiation, column chromatography to yield **23** (111 mg, 60%). *In MeCN solution of RB.* 5 min irradiation, column chromatography to yield **23** (107 mg, 58%). *In toluene solution of C_{60}.* 10 min irradiation, column chromatography to yield **23** (144 mg, 78%).

References

1. S. H. Lim, H. C. Jeong, Y. Sohn, Y. -I. Kim, D. W. Cho, H. -J. Woo, I. -S. Shin, U. C. Yoon and P.

S. Mariano, *J. Org. Chem.*, 2016, **81**, 2460;

2. S. H. Lim, J. Yi, G. M. Moon, C. S. Ra, K. Nahm, D. W. Cho, K. Kim, T. G. Hyung, U. C. Yoon, G. Y. Lee, S. Kim, J. Kim and P. S. Mariano, *J. Org. Chem.*, 2014, **79**, 6946.

3. S. H. Lim, D. W. Cho and P. S. Mariano, *Heterocycles*, 2016, **93**, 202.

DCA-fluorescence quenching experiment by *N*-benzyl glycinate. Fluorescence of MeCN solutions of DCA (3 mL, 2.5 x 10^{-6} M) containing 0, 0.2, 0.4, 1, 2 and 3 mM of the respective glycinate **16a, 16b, 16e** and **16f** were measured. The excitation wavelength was 365 nm. The spectra are displayed in Figure S1. The Stern-Volmer plots were determined by equation (1), where Φ_0 is the intensity of the fluorescence from the DCA in the absence of quencher (*i.e.*, *N*-benzylglycinate), Φ_q is the intensity of the DCA when the quencher is present at a concentration [Q], k_q is quenching rate constant and τ is lifetime of DCA. (τ_{S1} (DCA) =14.9 ns)

$$\Phi_0/\Phi_q = 1 + k_q\tau\,[Q] \qquad (1)$$

(a)

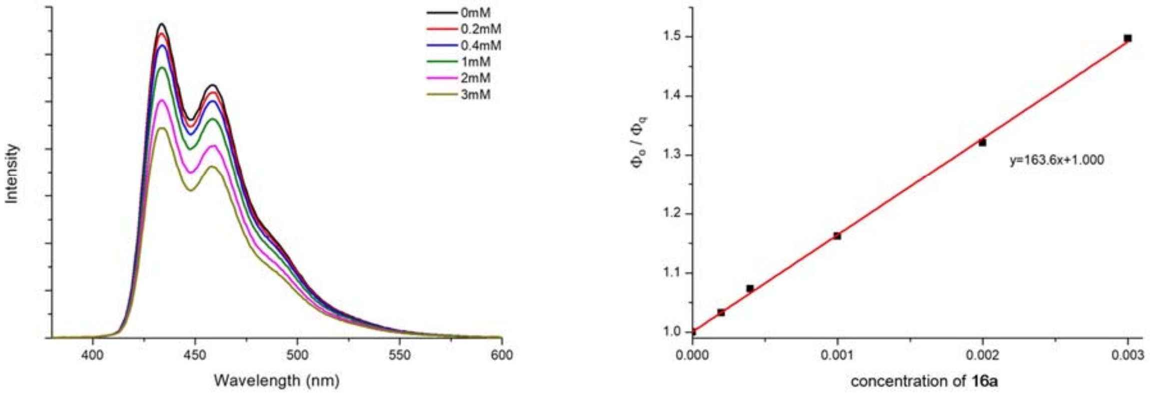

(b)

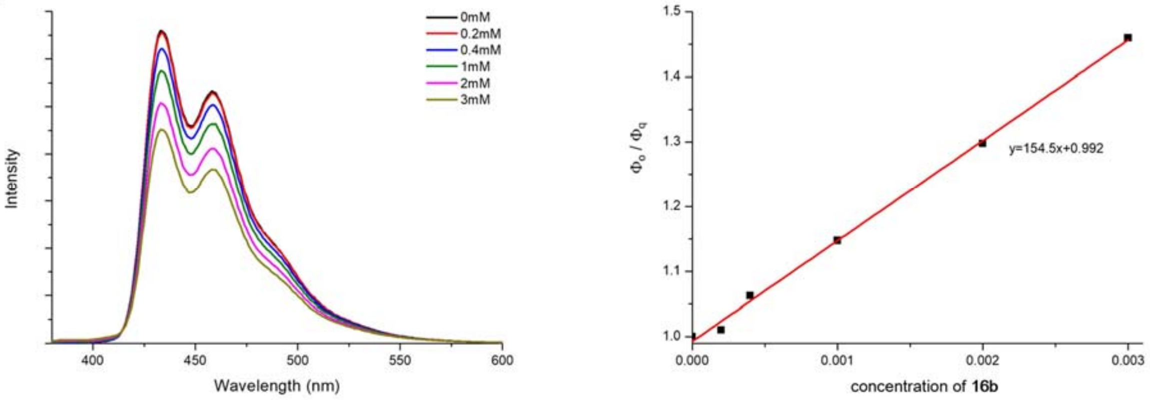

(c)

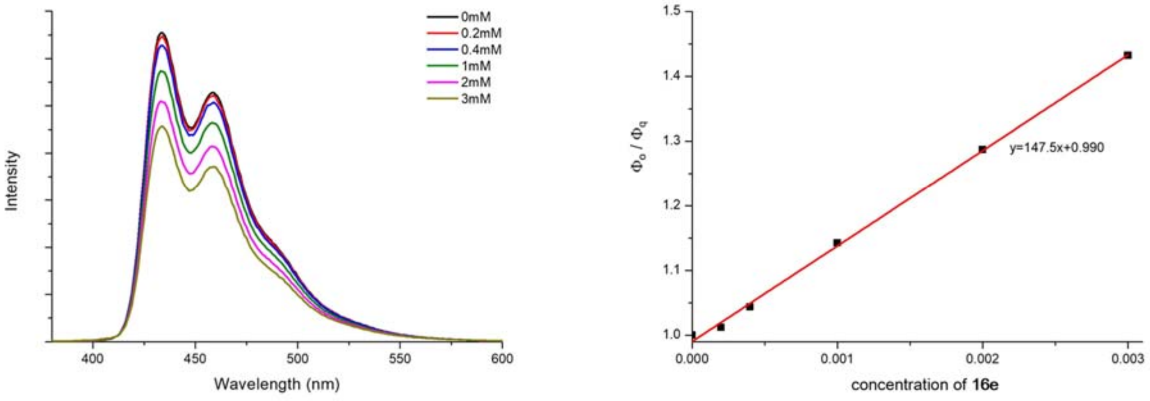

(d)

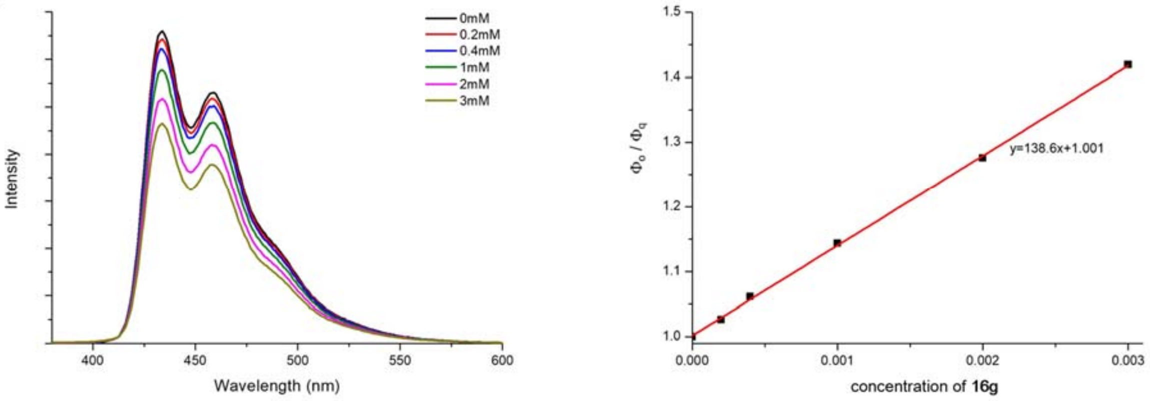

Figure S1. Fluorescence spectral changes of MeCN solutions of DCA (2.5 x 10^{-6} M) upon addition of *N*-benzylglycinates (a) **16a**, (b) **16b**, (c) **16e**, (d) **16f** (λ_{ex} = 365 nm) and Stern-Volmer plot of *N*-benzyl glycinates concentration dependence of the fluorescence intensity of DCA. (τ_{S1} (DCA) =14.9 ns)

DCN-fluorescence quenching experiment by *N*-benzylglycinate. Fluorescence of MeCN solutions of DCN (3 mL, 5 x 10^{-4} M) containing 0, 0.2, 0.4, 1, 2 and 3 mM of the respective glycinate **16a, 16b, 16e** and **16f** were measured. The excitation wavelength was 330 nm. The spectra are displayed in Figure S2. The Stern-Volmer plots were determined by equation (1), where Φ_0 is the intensity of the fluorescence from the DCN in the absence of *N*-benzylglycinate, Φ_q is the intensity of the DCN when the *N*-benzylglycinate is present at a certain concentration [Q], k_q is quenching rate constant and τ is lifetime of DCN. (τ_{S1} (DCN) =10.3 ns)

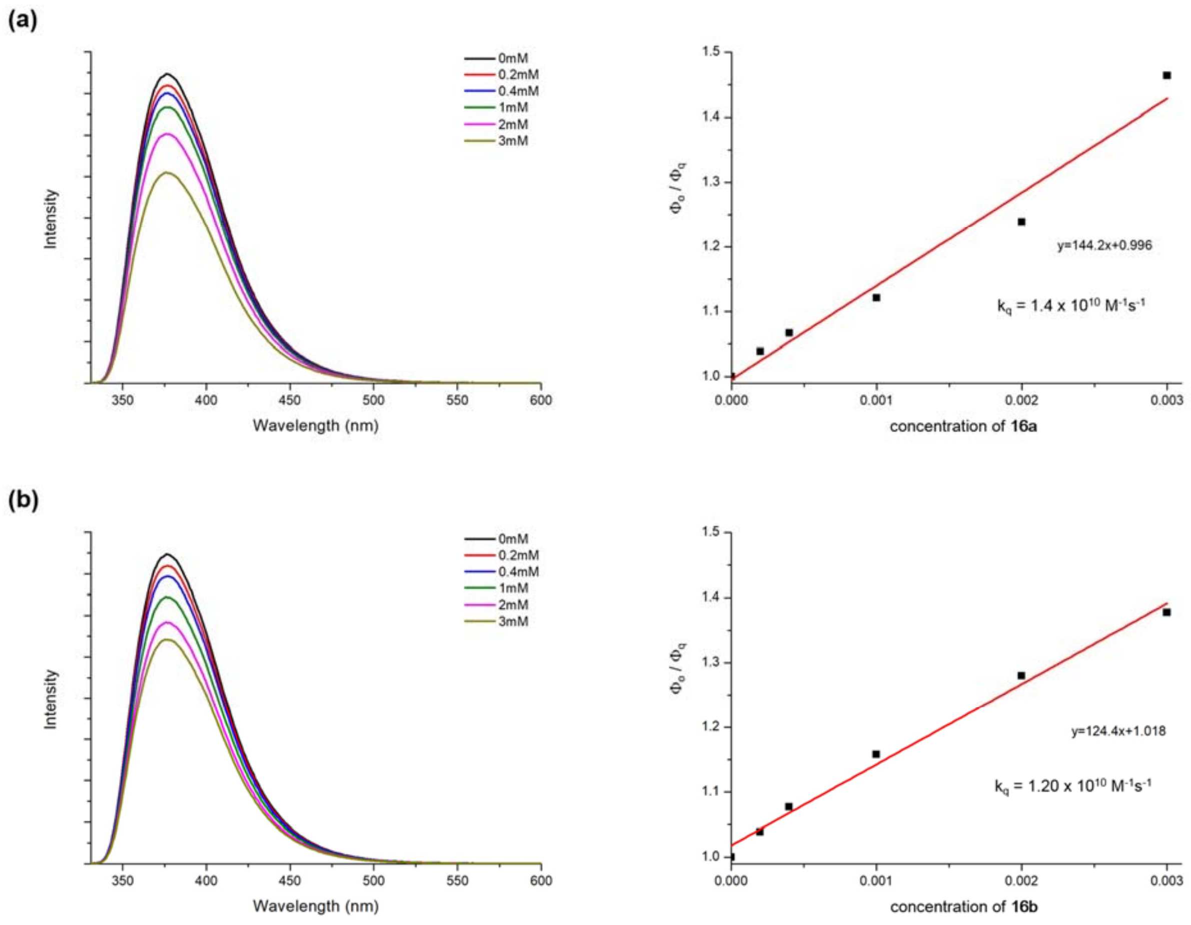

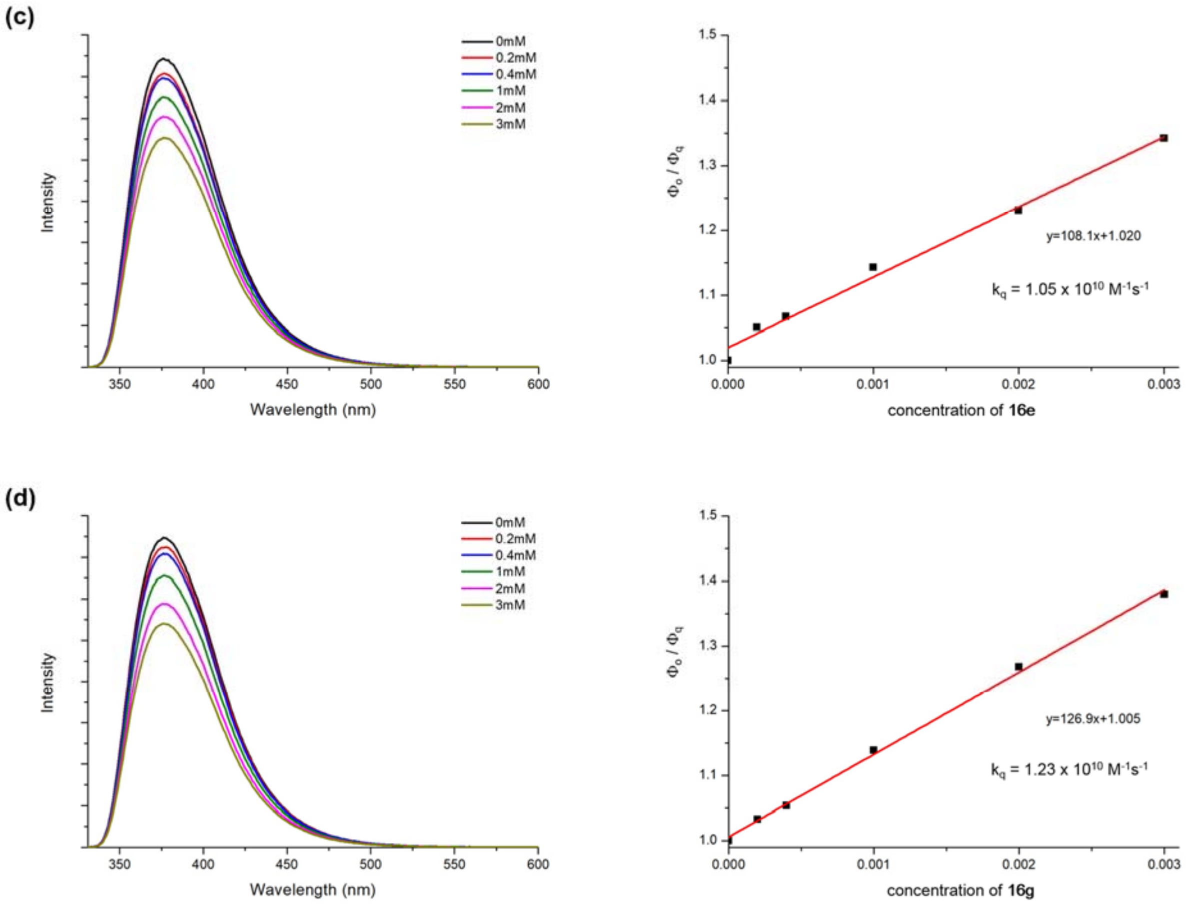

Figure S2. Fluorescence spectral changes of MeCN solutions of DCN (5 x 10^{-4} M) upon addition of *N*-benzylglycinates (a) **16a**, (b) **16b**, (c) **16e**, (d) **16f** (λ_{ex} = 365 nm) and Stern-Volmer plot of *N*-benzyl glycinates concentration dependence of the fluorescence intensity of DCA. (τ_{S1} (DCN) =10.3 ns)

16c

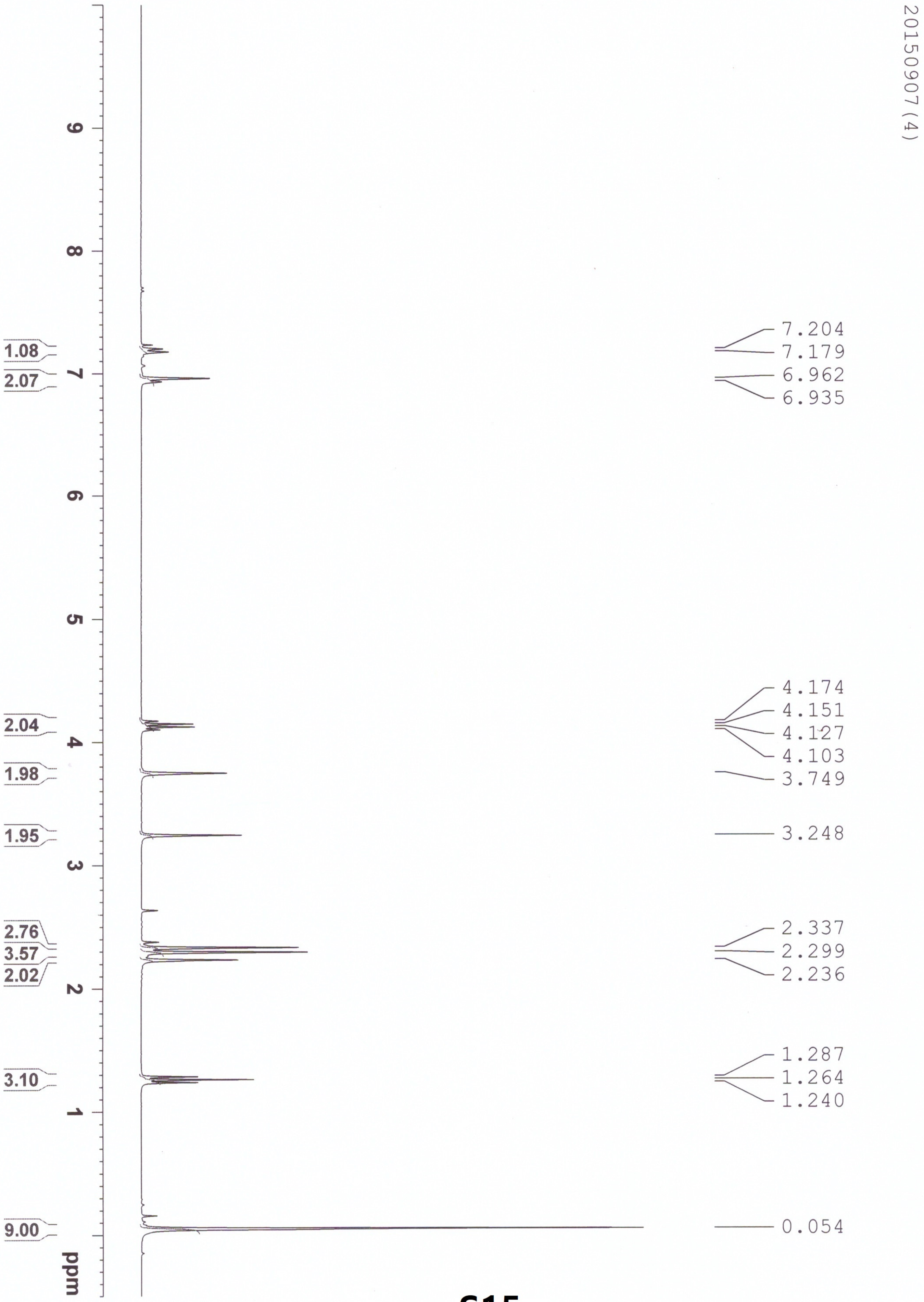
20150907(4)
7.204
7.179
6.962
6.935
4.174
4.151
4.127
4.103
3.749
3.248
2.337
2.299
2.236
1.287
1.264
1.240
0.054
1.08
2.07
2.04
1.98
1.95
2.76
3.57
2.02
3.10
9.00
ppm

16c

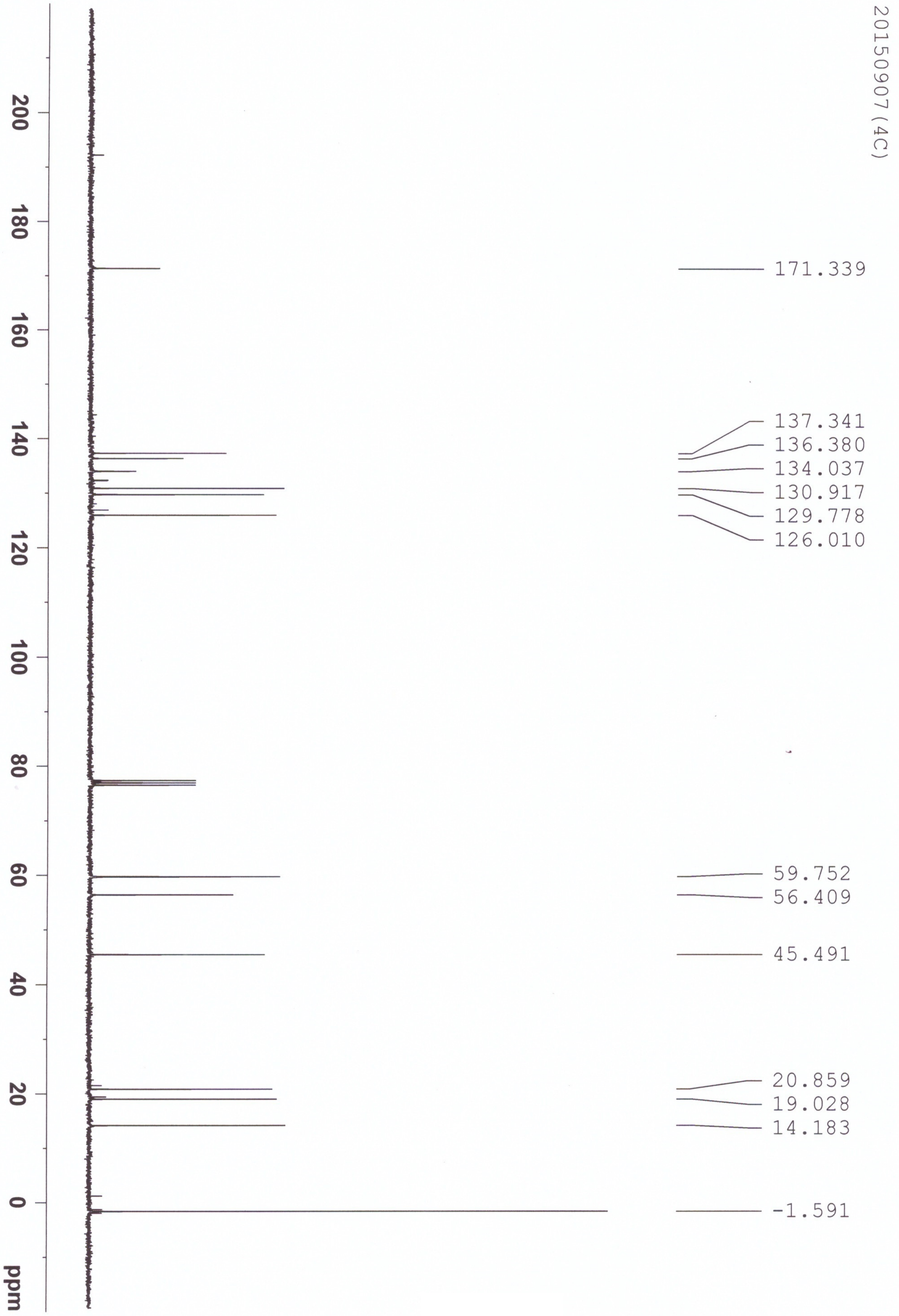
20150907(4C)
171.339
137.341
136.380
134.037
130.917
129.778
126.010
59.752
56.409
45.491
20.859
19.028
14.183
-1.591
200
180
160
140
120
100
80
60
40
20
0
ppm

16f

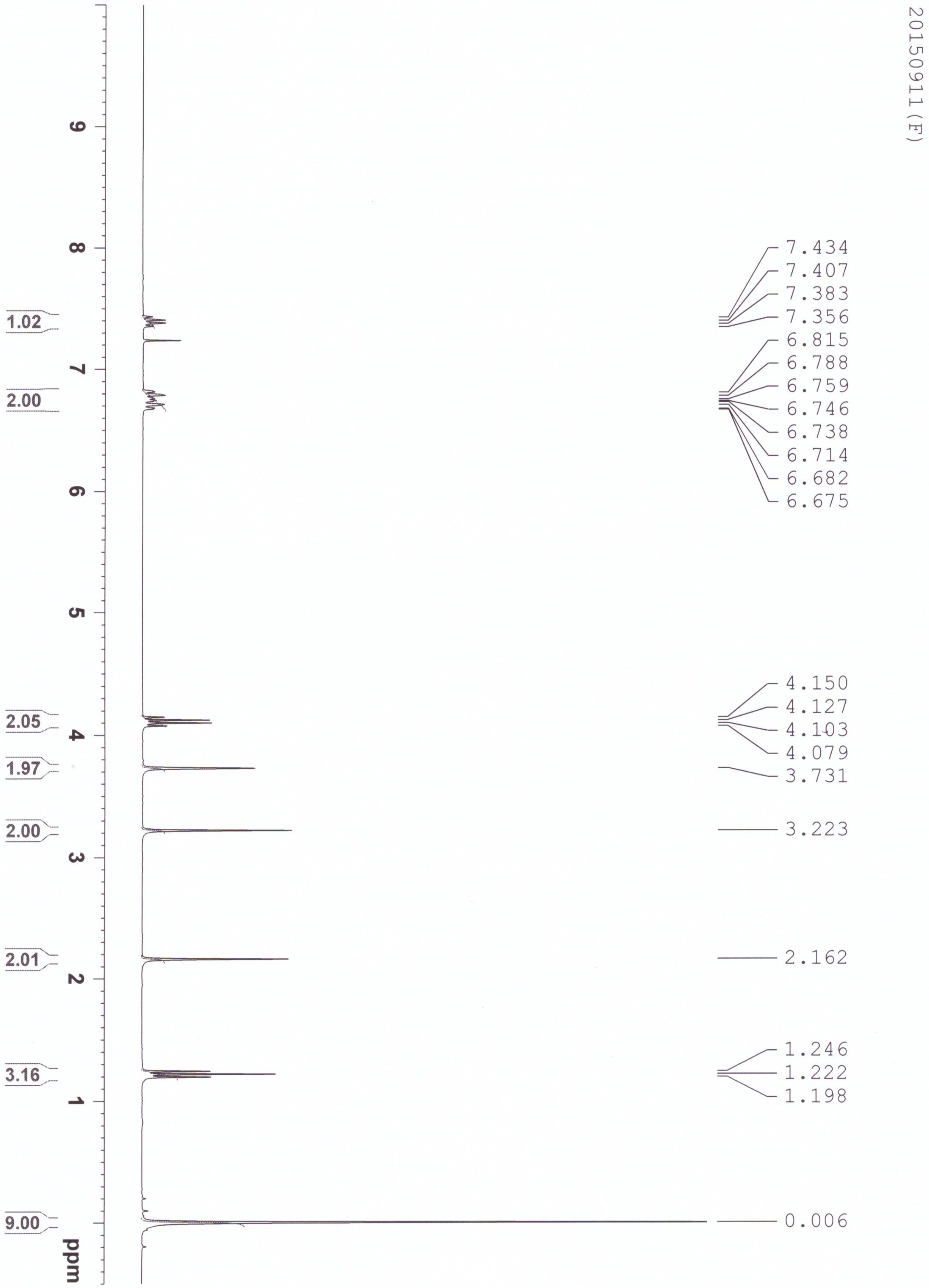
20150911(F)
7.434
7.407
7.383
7.356
6.815
6.788
6.759
6.746
6.738
6.714
6.682
6.675
4.150
4.127
4.103
4.079
3.731
3.223
2.162
1.246
1.222
1.198
0.006
1.02
2.00
2.05
1.97
2.00
2.01
3.16
9.00
9
8
7
6
5
4
3
2
1
ppm

16f

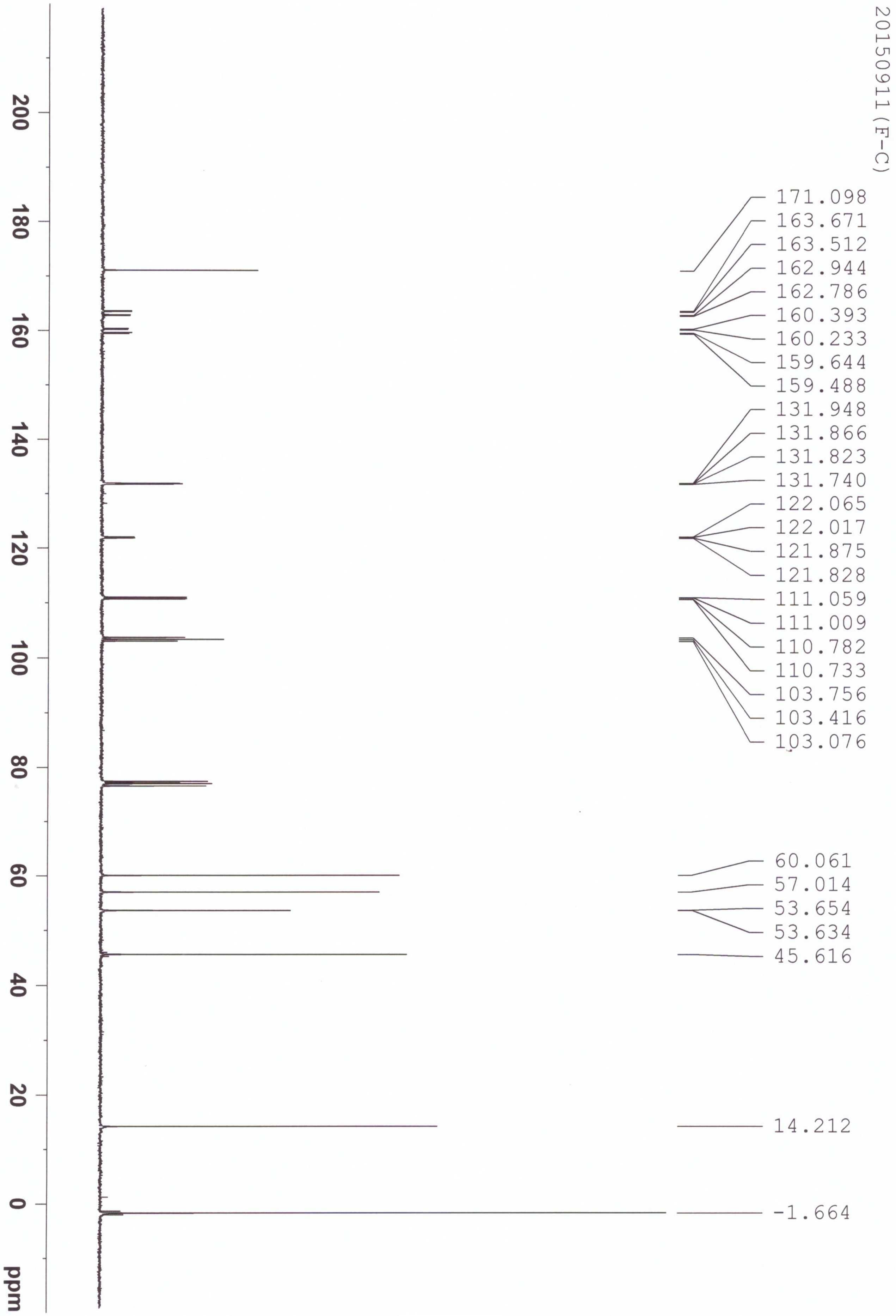

20150911(F-C)
171.098
163.671
163.512
162.944
162.786
160.393
160.233
159.644
159.488
131.948
131.866
131.823
131.740
122.065
122.017
121.875
121.828
111.059
111.009
110.782
110.733
103.756
103.416
103.076
60.061
57.014
53.654
53.634
45.616
14.212
-1.664
200
180
160
140
120
100
80
60
40
20
0
ppm

18a

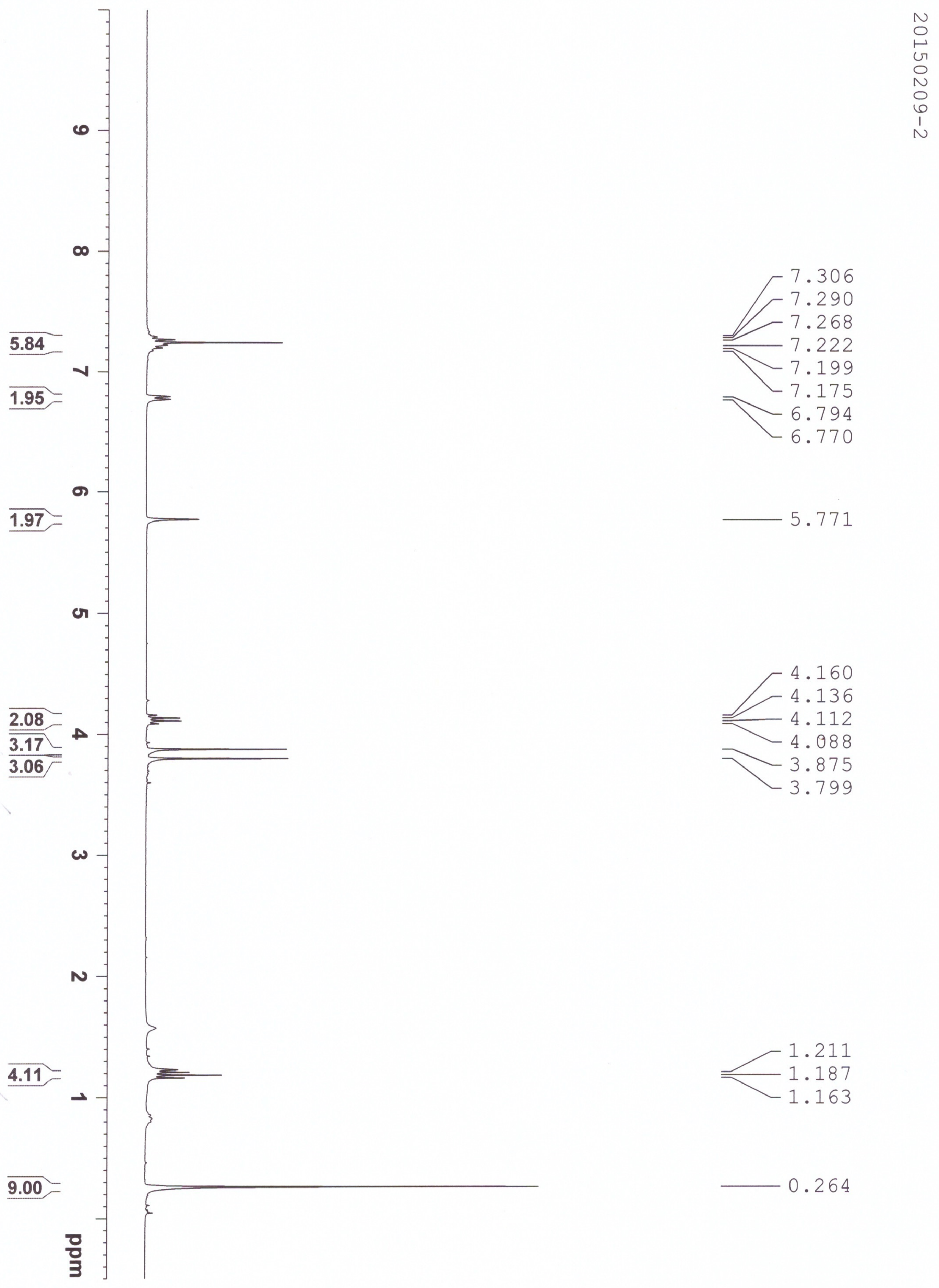
20150209-2
7.306
7.290
7.268
7.222
7.199
7.175
6.794
6.770
5.771
4.160
4.136
4.112
4.088
3.875
3.799
1.211
1.187
1.163
0.264
5.84
1.95
1.97
2.08
3.17
3.06
4.11
9.00
ppm

18a

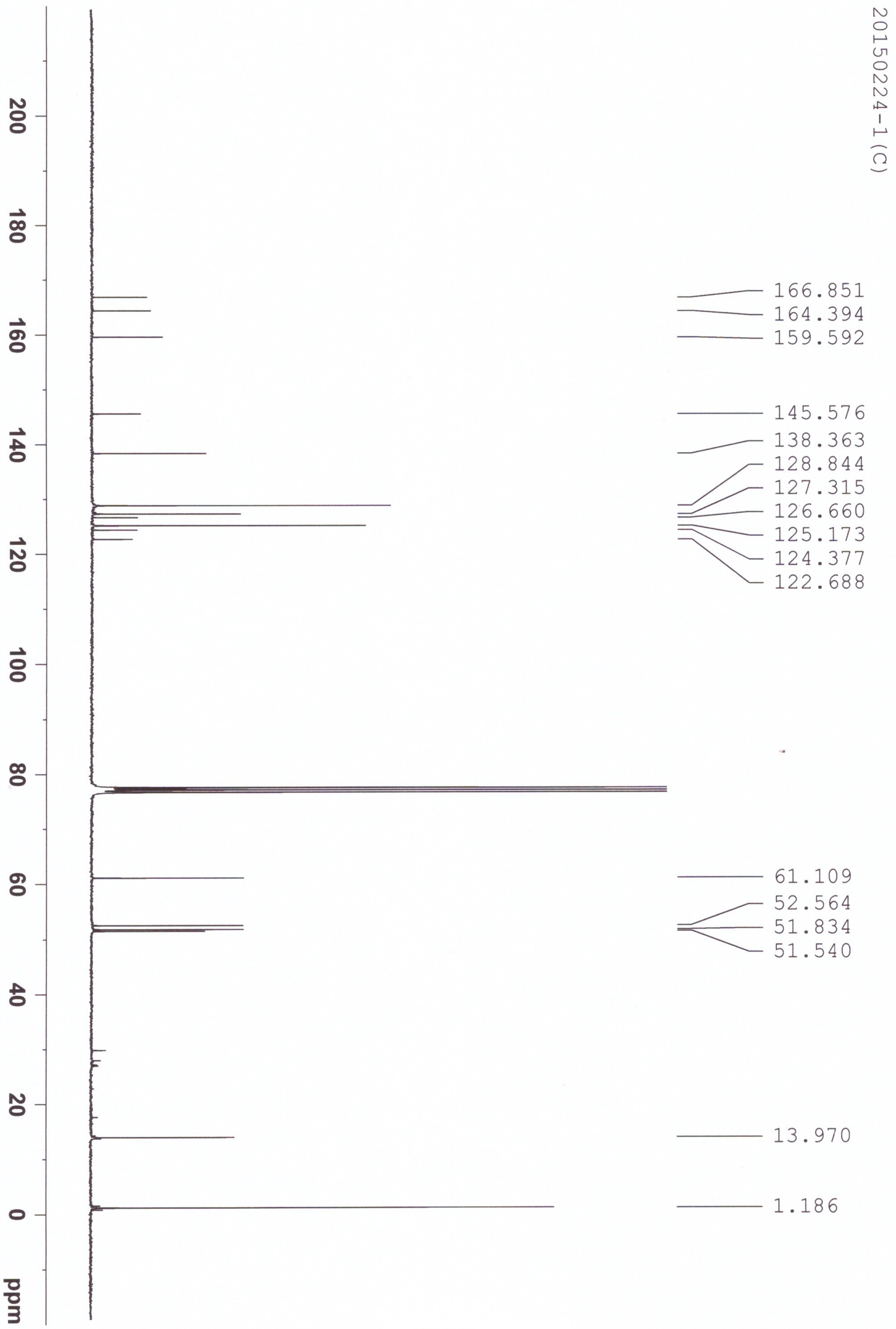
20150224-1(C)
166.851
164.394
159.592
145.576
138.363
128.844
127.315
126.660
125.173
124.377
122.688
61.109
52.564
51.834
51.540
13.970
1.186
200
180
160
140
120
100
80
60
40
20
0
ppm

19a

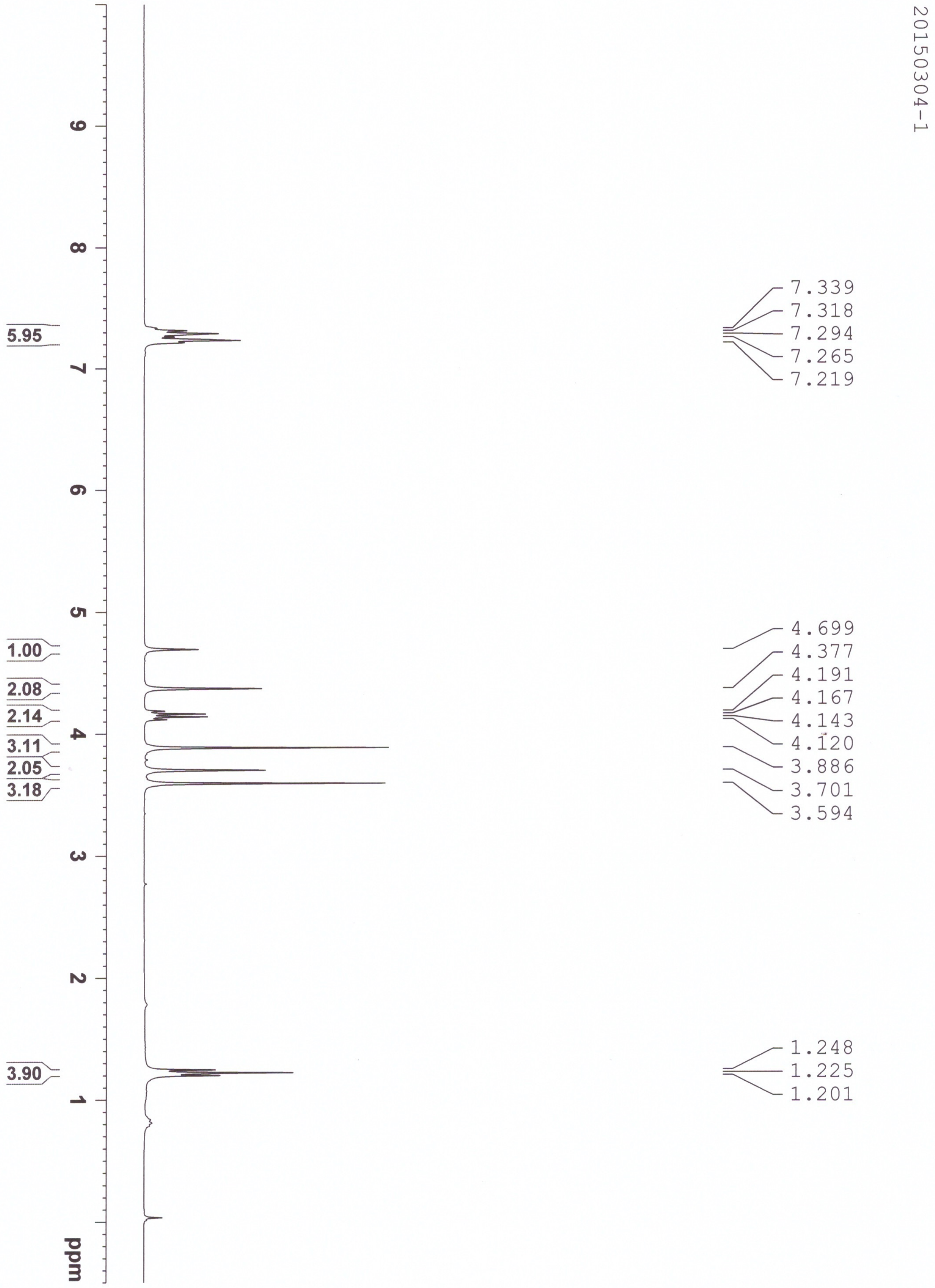
20150304-1
7.339
7.318
7.294
7.265
7.219
4.699
4.377
4.191
4.167
4.143
4.120
3.886
3.701
3.594
1.248
1.225
1.201
5.95
1.00
2.08
2.14
3.11
2.05
3.18
3.90
9
8
7
6
5
4
3
2
1
ppm

19a

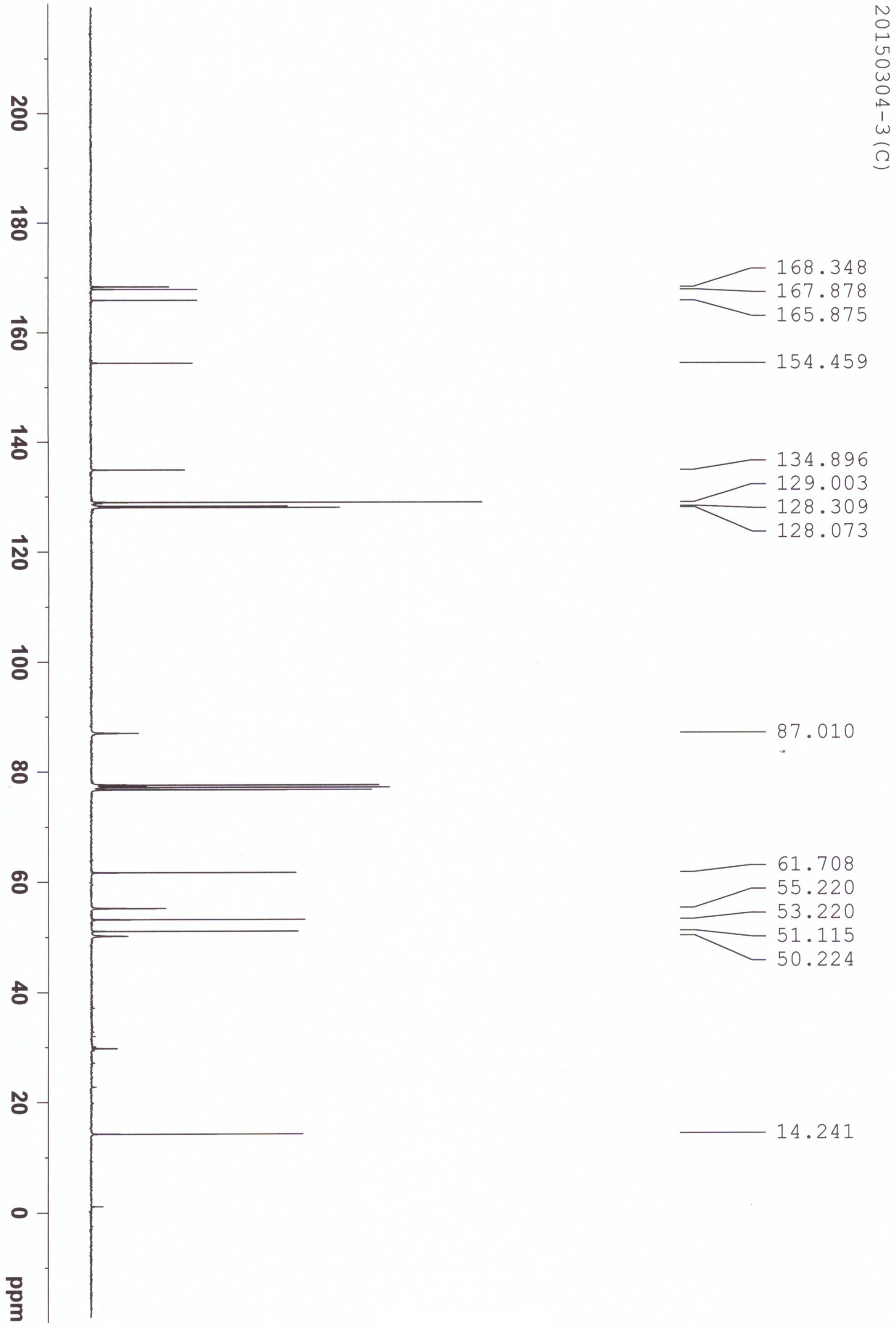
20150304-3(C)
168.348
167.878
165.875
154.459
134.896
129.003
128.309
128.073
87.010
61.708
55.220
53.220
51.115
50.224
14.241
200
180
160
140
120
100
80
60
40
20
0
ppm

18b

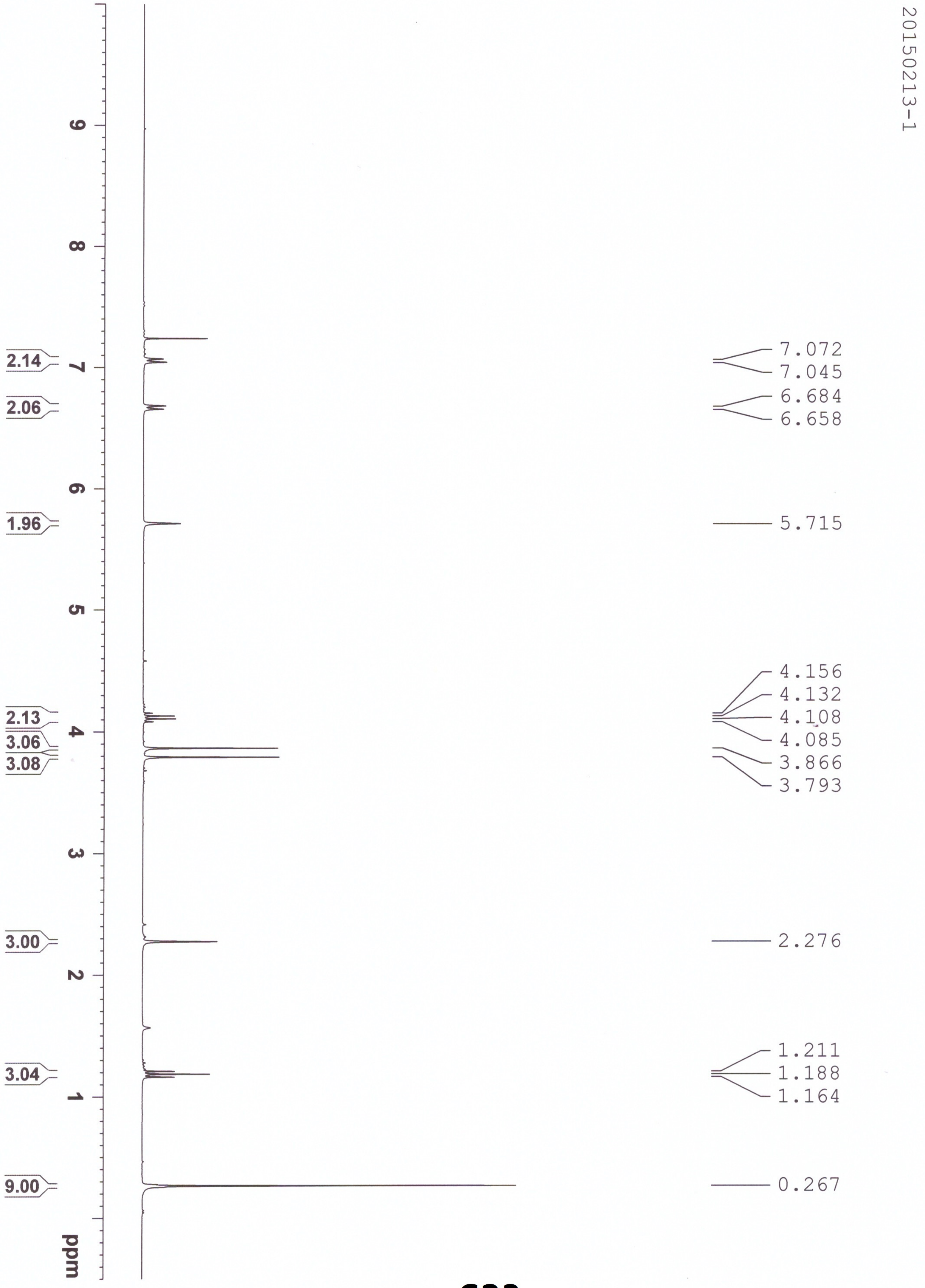

20150213-1
7.072
7.045
6.684
6.658
5.715
4.156
4.132
4.108
4.085
3.866
3.793
2.276
1.211
1.188
1.164
0.267
2.14
2.06
1.96
2.13
3.06
3.08
3.00
3.04
9.00
9
8
7
6
5
4
3
2
1
ppm

18b

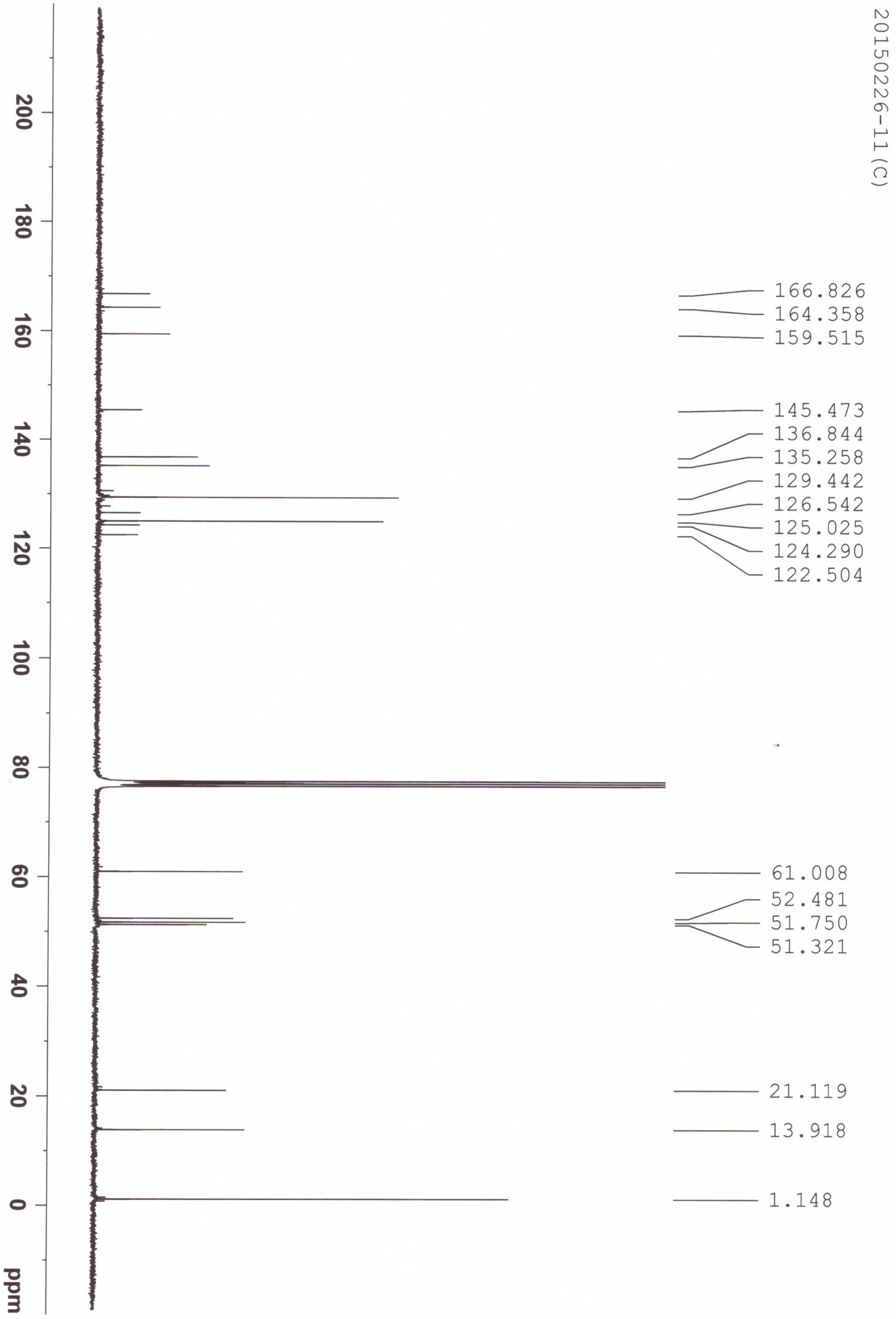
20150226-11(C)
166.826
164.358
159.515
145.473
136.844
135.258
129.442
126.542
125.025
124.290
122.504
61.008
52.481
51.750
51.321
21.119
13.918
1.148
200
180
160
140
120
100
80
60
40
20
0
ppm

19b

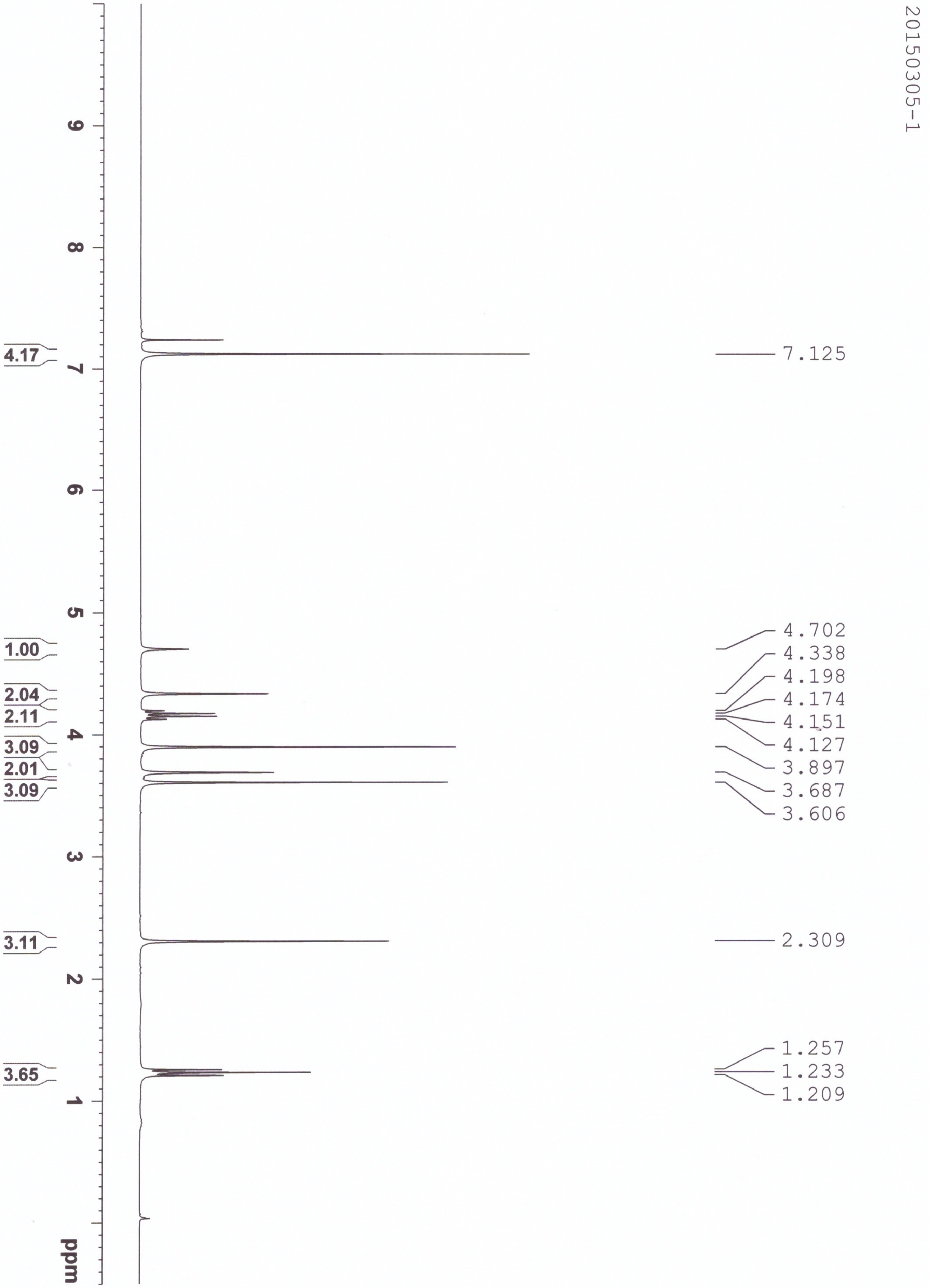
20150305-1
7.125
4.702
4.338
4.198
4.174
4.151
4.127
3.897
3.687
3.606
2.309
1.257
1.233
1.209
4.17
1.00
2.04
2.11
3.09
2.01
3.09
3.11
3.65
ppm

19b

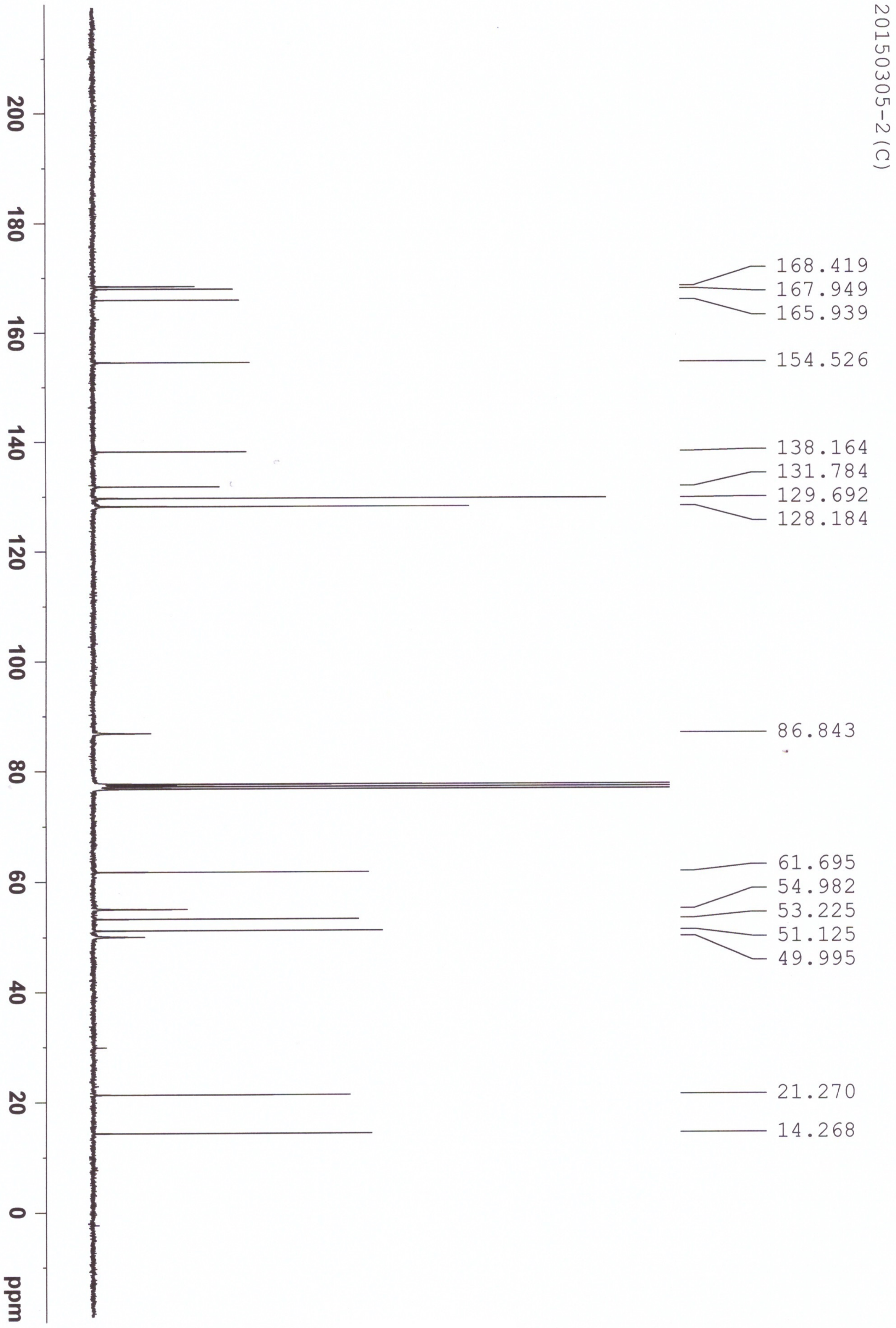
20150305-2(C)
168.419
167.949
165.939
154.526
138.164
131.784
129.692
128.184
86.843
61.695
54.982
53.225
51.125
49.995
21.270
14.268
200
180
160
140
120
100
80
60
40
20
0
ppm

18c

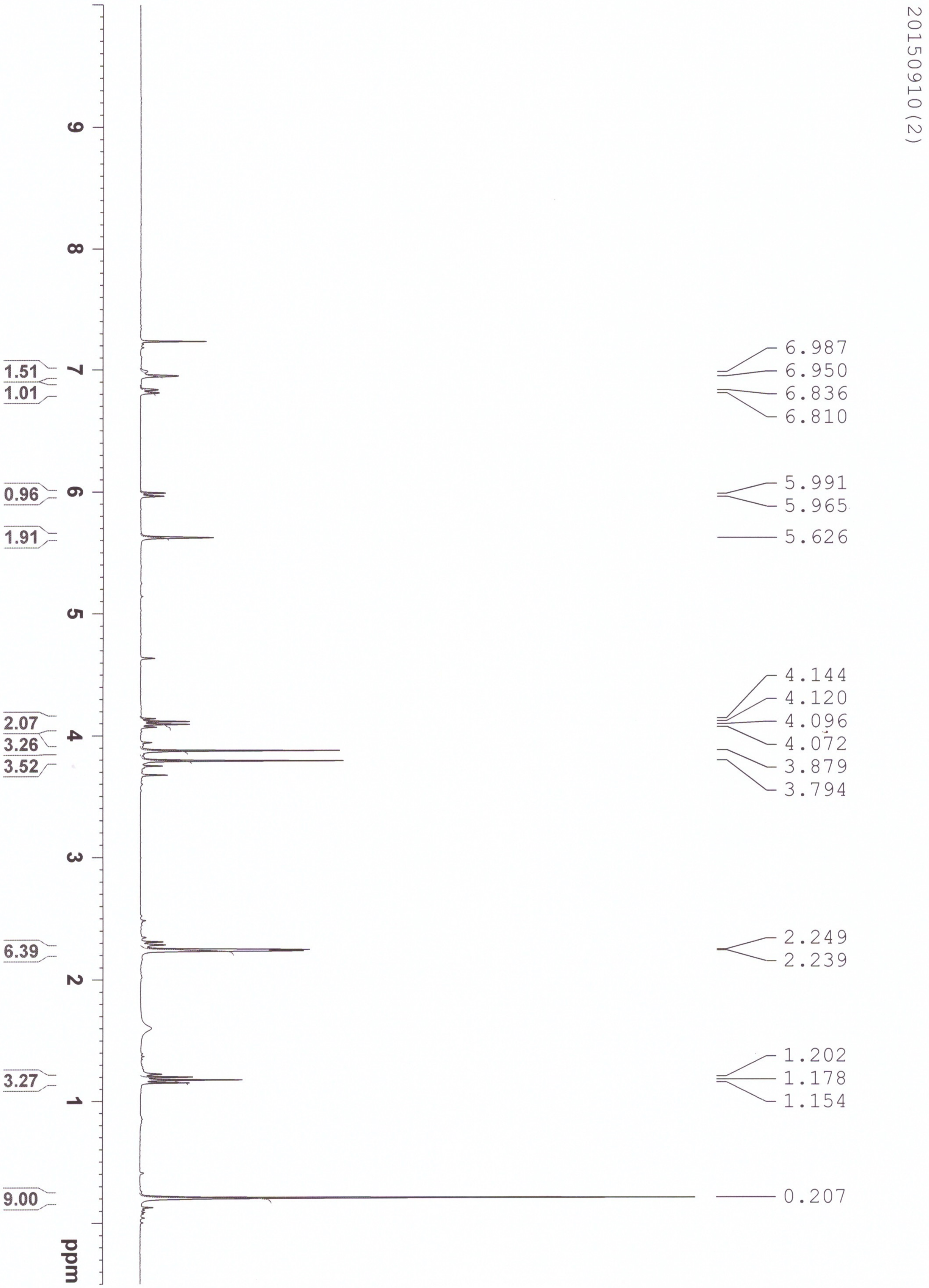
6.987
6.950
6.836
6.810
5.991
5.965
5.626
4.144
4.120
4.096
4.072
3.879
3.794
2.249
2.239
1.202
1.178
1.154
0.207
1.51
1.01
0.96
1.91
2.07
3.26
3.52
6.39
3.27
9.00
ppm
20150910(2)

18c

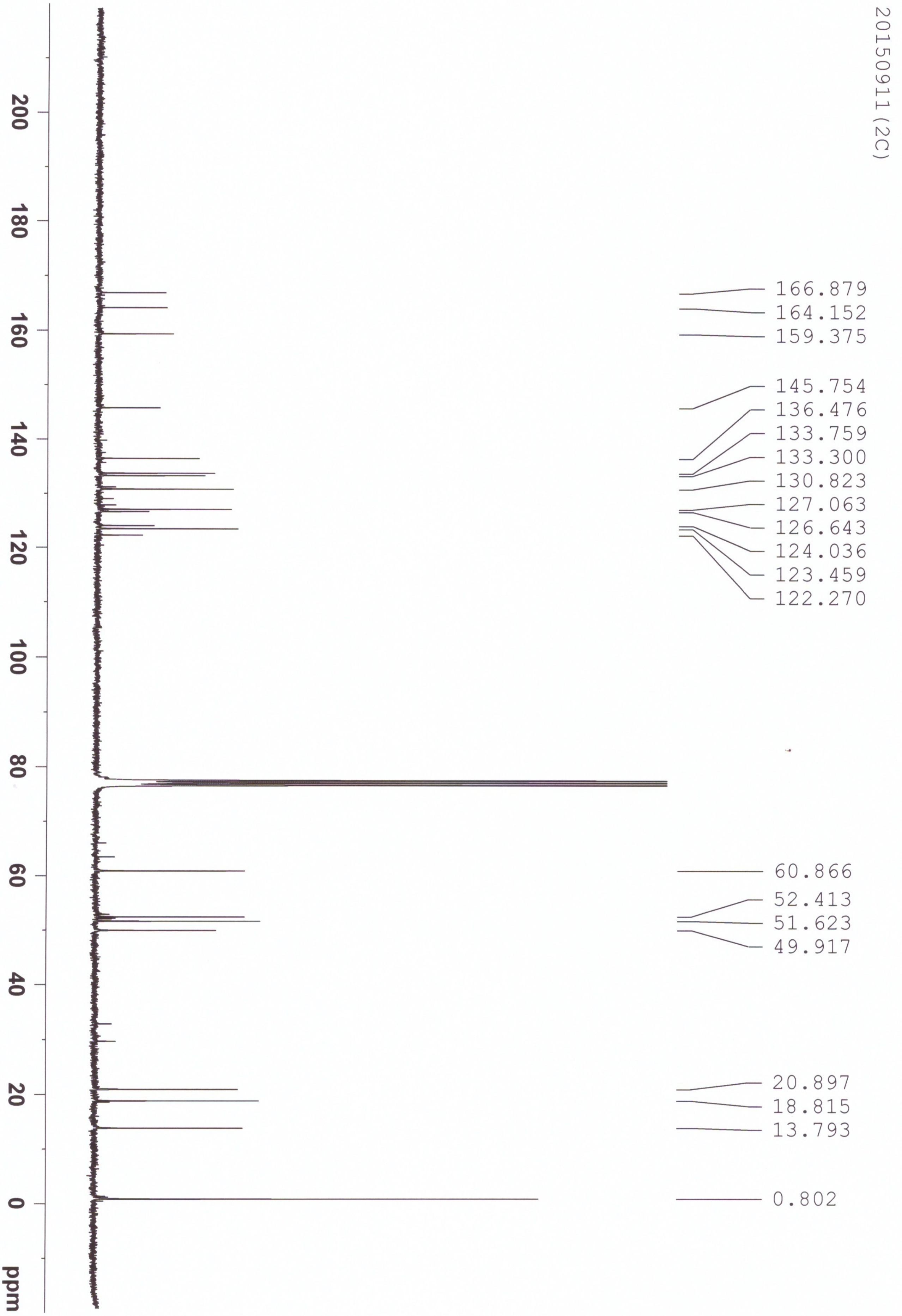
20150911(2C)
166.879
164.152
159.375
145.754
136.476
133.759
133.300
130.823
127.063
126.643
124.036
123.459
122.270
60.866
52.413
51.623
49.917
20.897
18.815
13.793
0.802
200
180
160
140
120
100
80
60
40
20
0
ppm

19c

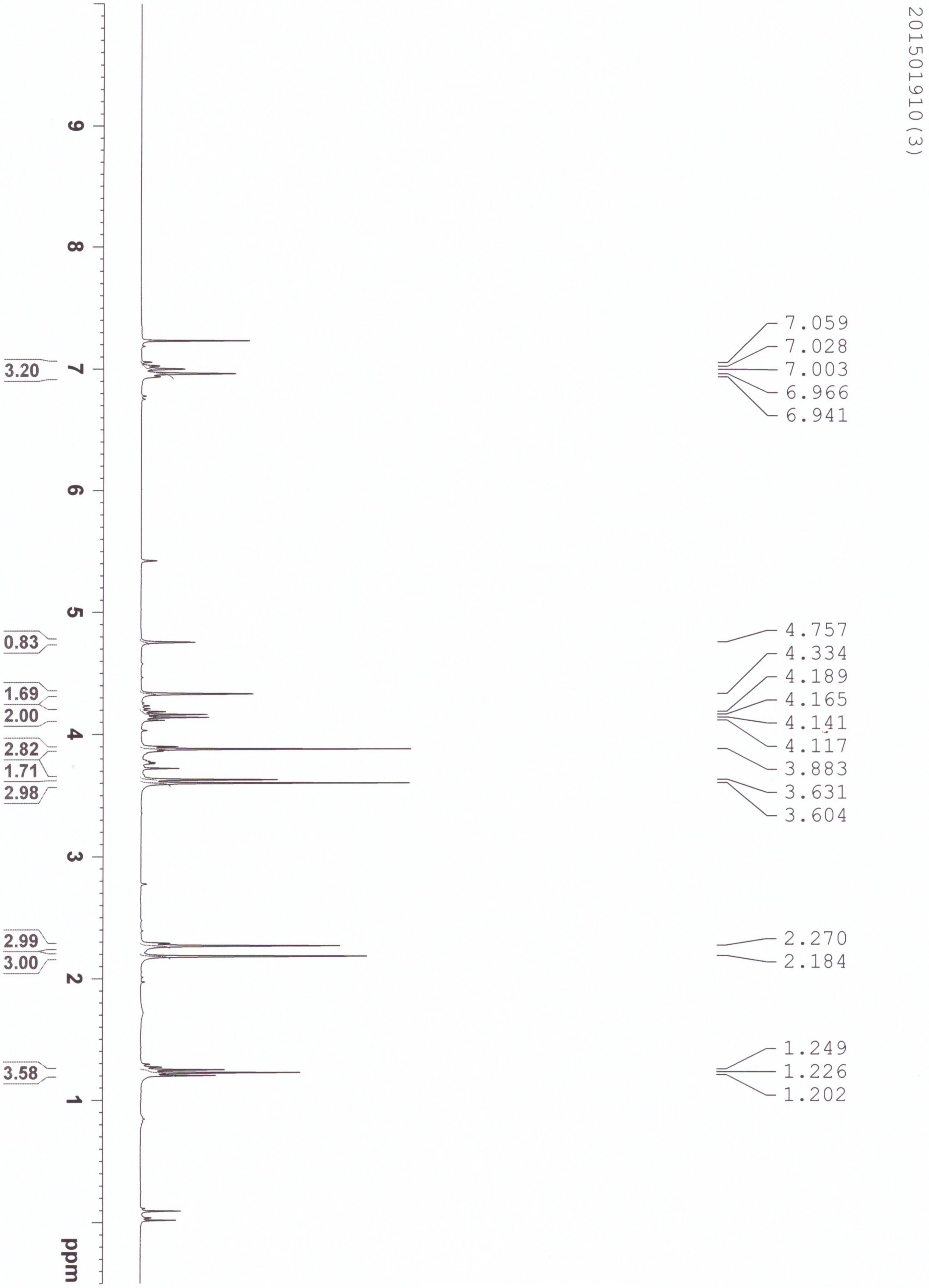

201501910(3)
7.059
7.028
7.003
6.966
6.941
4.757
4.334
4.189
4.165
4.141
4.117
3.883
3.631
3.604
2.270
2.184
1.249
1.226
1.202
3.20
0.83
1.69
2.00
2.82
1.71
2.98
2.99
3.00
3.58
ppm

19c

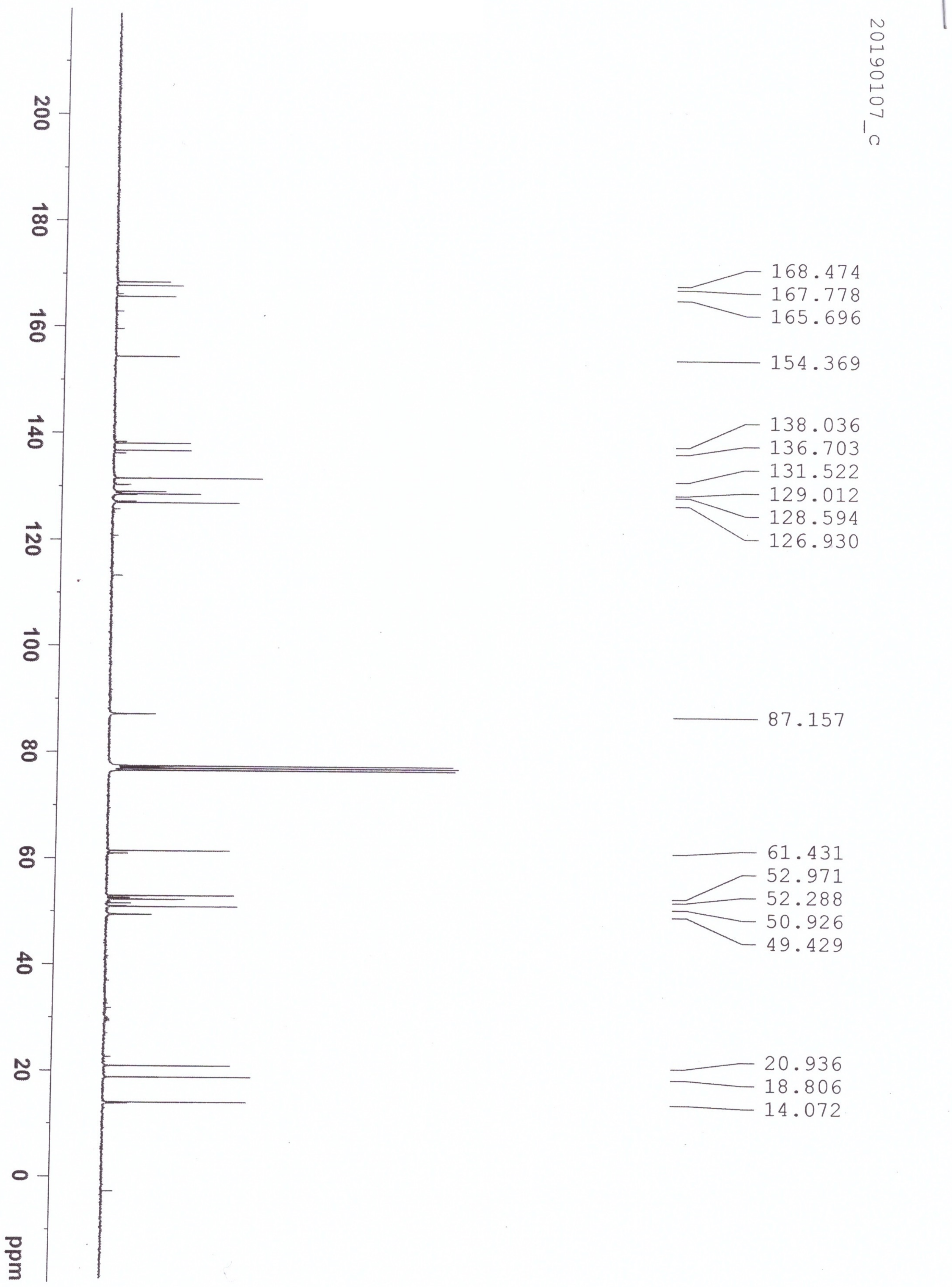
20190107_c
168.474
167.778
165.696
154.369
138.036
136.703
131.522
129.012
128.594
126.930
87.157
61.431
52.971
52.288
50.926
49.429
20.936
18.806
14.072
200
180
160
140
120
100
80
60
40
20
0
ppm

18d

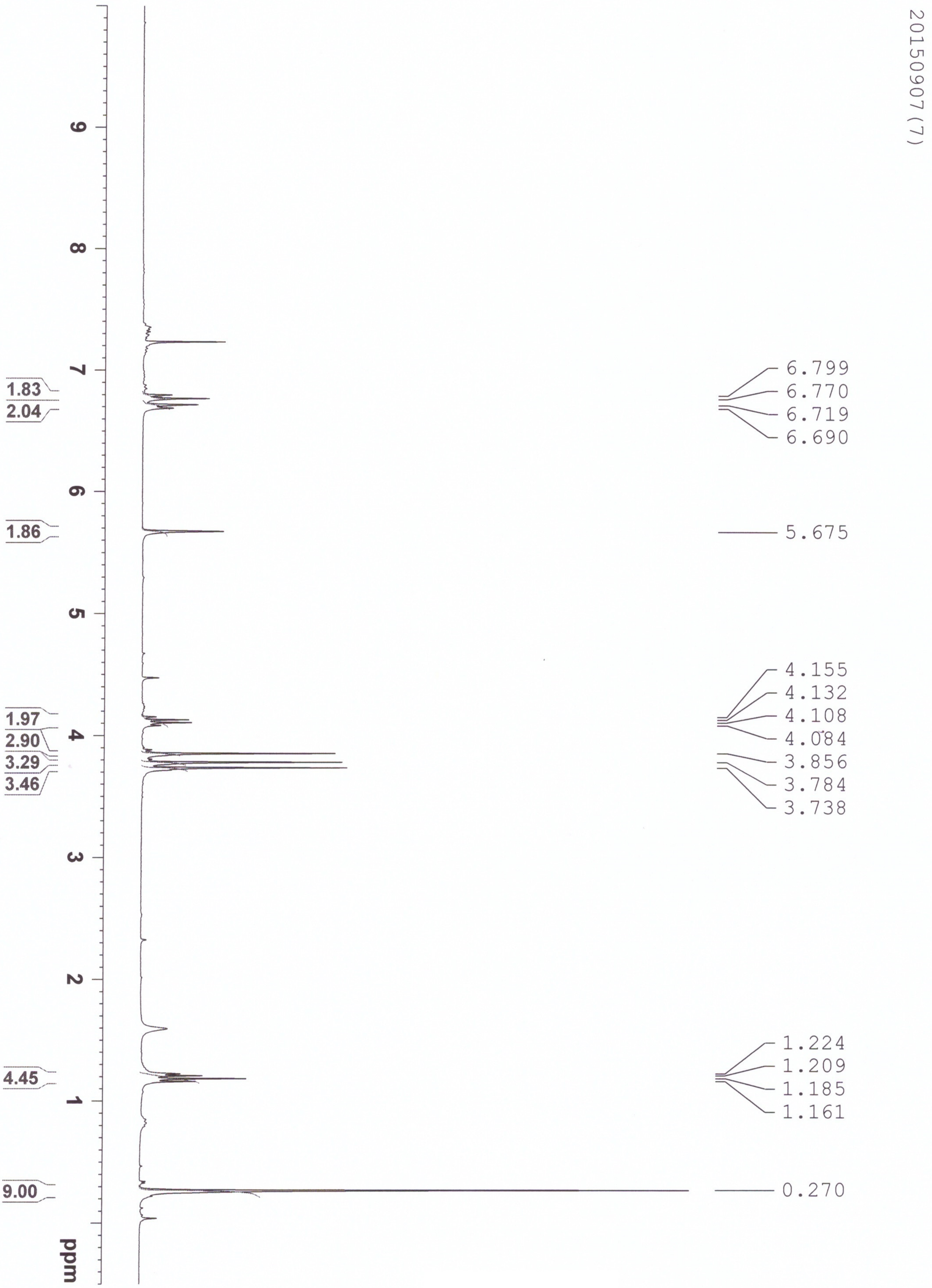
20150907(7)
6.799
6.770
6.719
6.690
5.675
4.155
4.132
4.108
4.084
3.856
3.784
3.738
1.224
1.209
1.185
1.161
0.270
1.83
2.04
1.86
1.97
2.90
3.29
3.46
4.45
9.00
9
8
7
6
5
4
3
2
1
ppm

18d

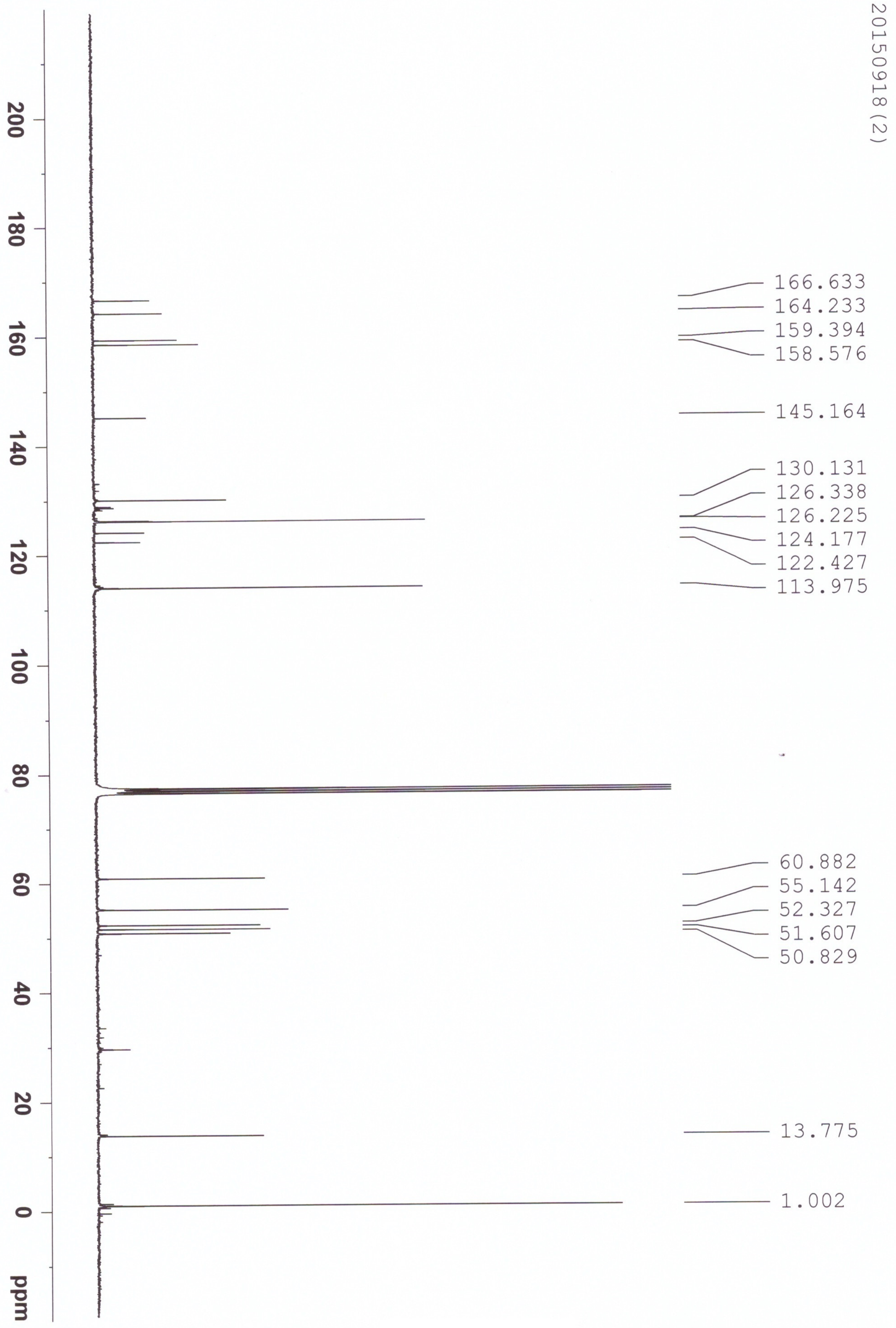
20150918(2)
166.633
164.233
159.394
158.576
145.164
130.131
126.338
126.225
124.177
122.427
113.975
60.882
55.142
52.327
51.607
50.829
13.775
1.002
ppm

19d

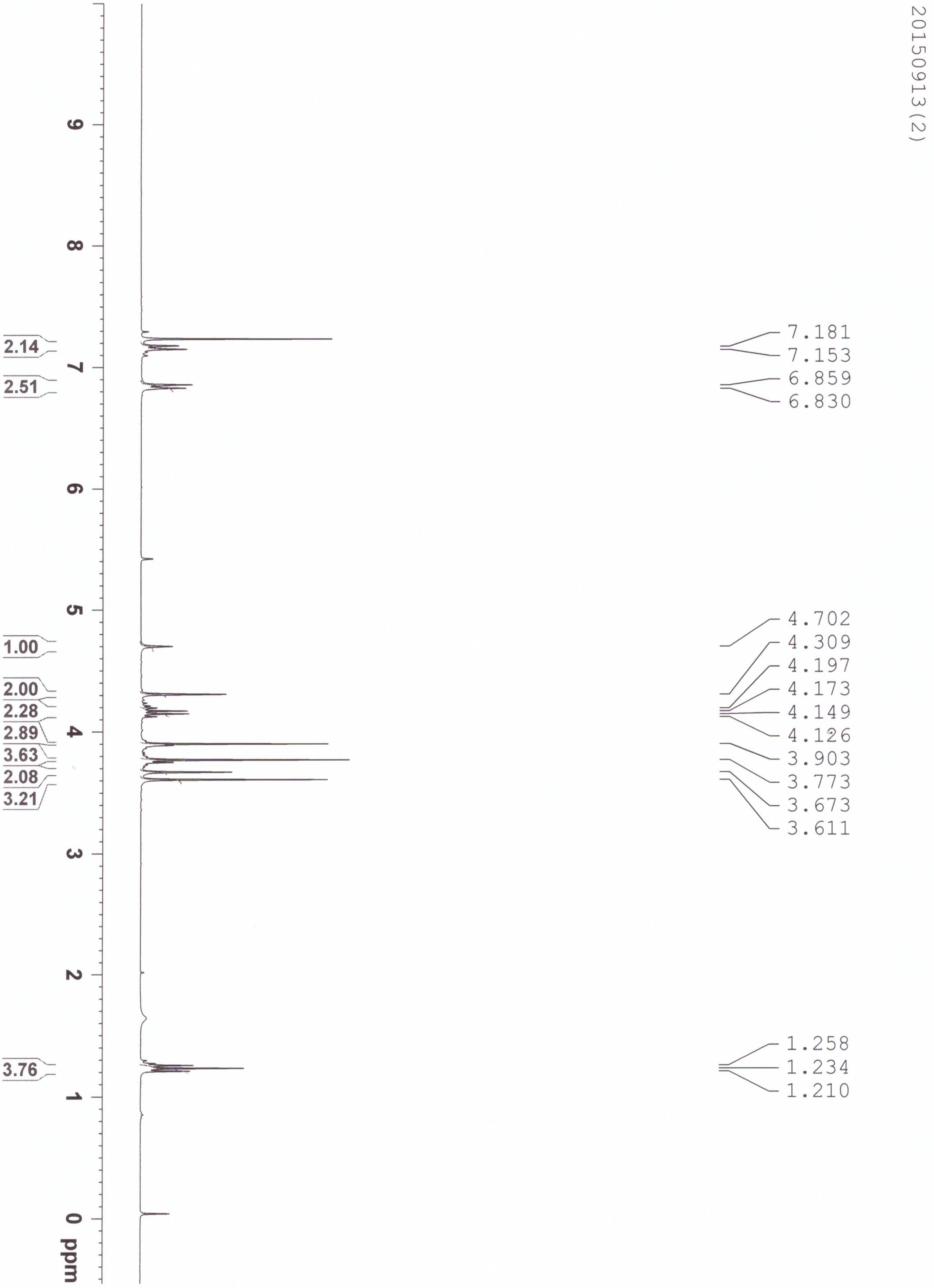
20150913(2)
7.181
7.153
6.859
6.830
4.702
4.309
4.197
4.173
4.149
4.126
3.903
3.773
3.673
3.611
1.258
1.234
1.210
2.14
2.51
1.00
2.00
2.28
2.89
3.63
2.08
3.21
3.76
9
8
7
6
5
4
3
2
1
0 ppm

19d

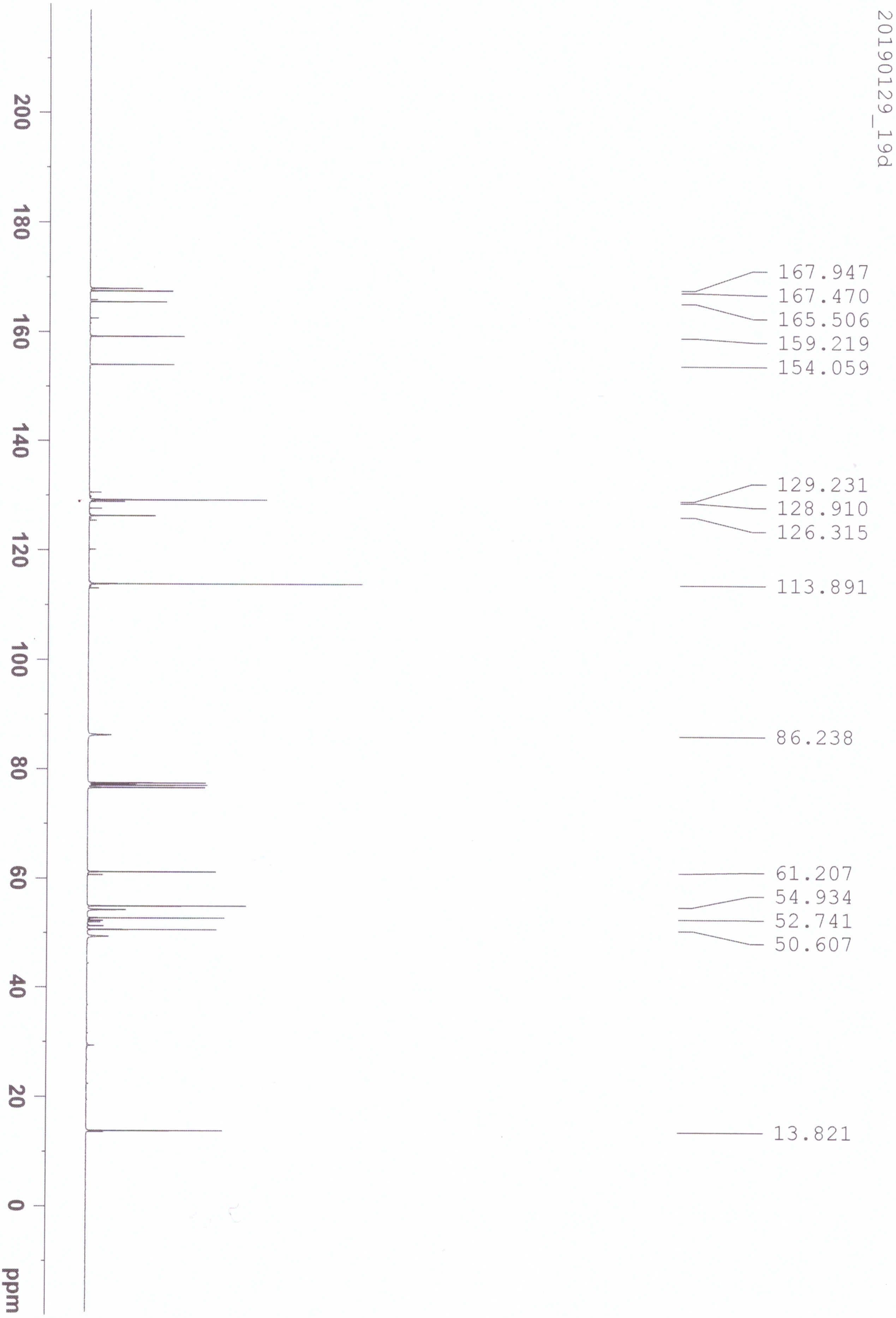
20190129_19d
167.947
167.470
165.506
159.219
154.059
129.231
128.910
126.315
113.891
86.238
61.207
54.934
52.741
50.607
13.821
200
180
160
140
120
100
80
60
40
20
0
ppm

18e

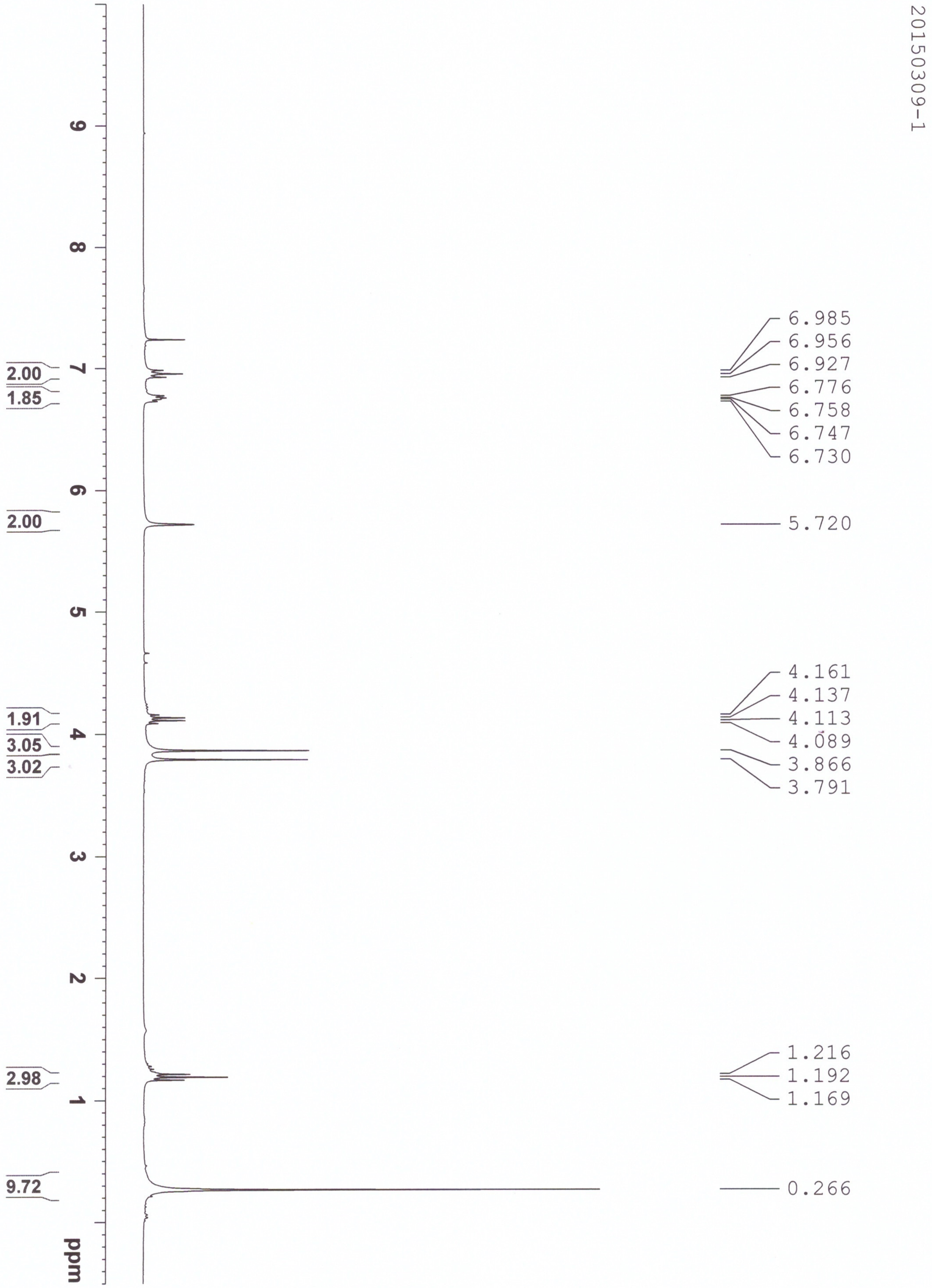
20150309-1
6.985
6.956
6.927
6.776
6.758
6.747
6.730
5.720
4.161
4.137
4.113
4.089
3.866
3.791
1.216
1.192
1.169
0.266
2.00
1.85
2.00
1.91
3.05
3.02
2.98
9.72
9
8
7
6
5
4
3
2
1
ppm

18e

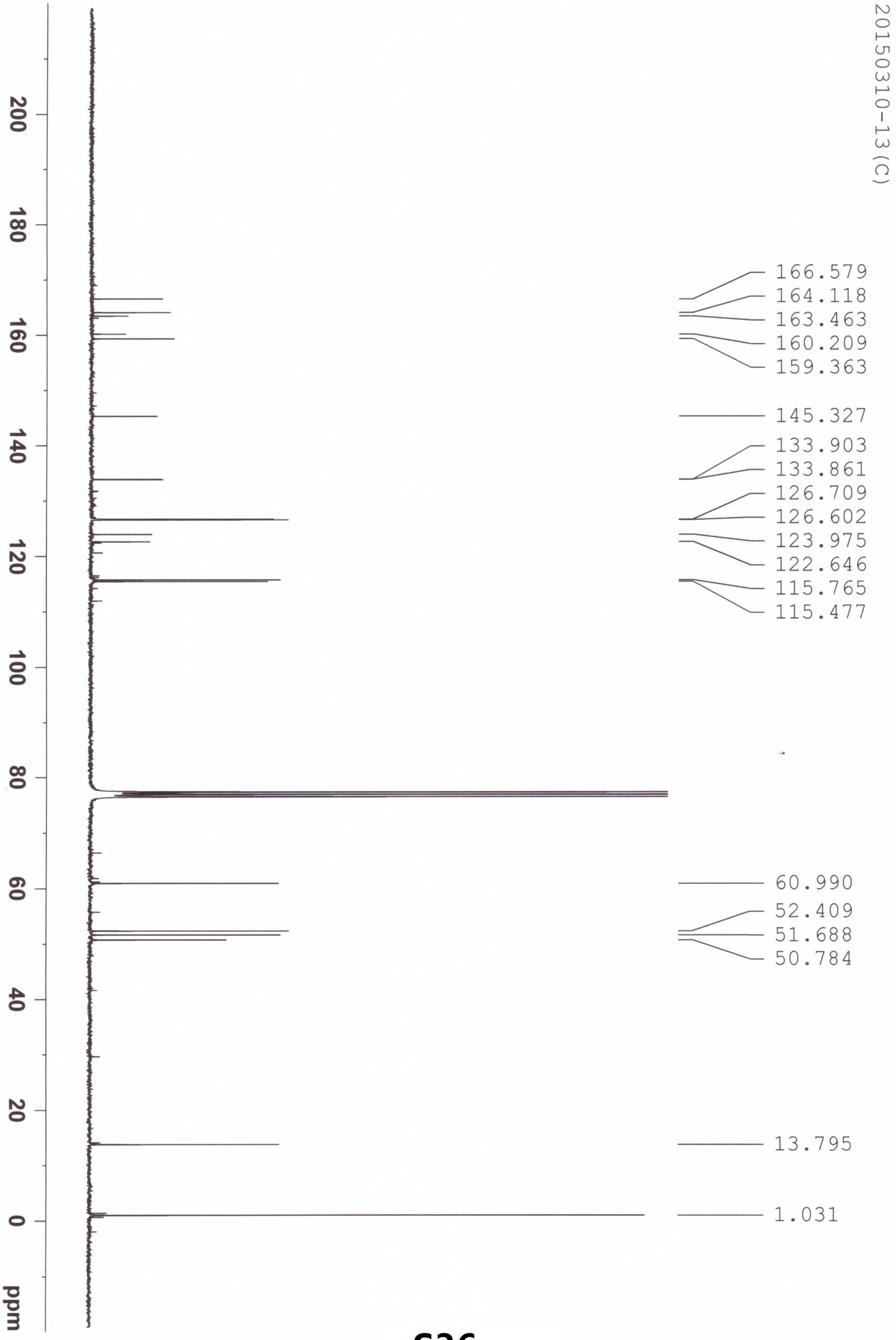
20150310-13(C)
166.579
164.118
163.463
160.209
159.363
145.327
133.903
133.861
126.709
126.602
123.975
122.646
115.765
115.477
60.990
52.409
51.688
50.784
13.795
1.031
200
180
160
140
120
100
80
60
40
20
0
ppm

19e

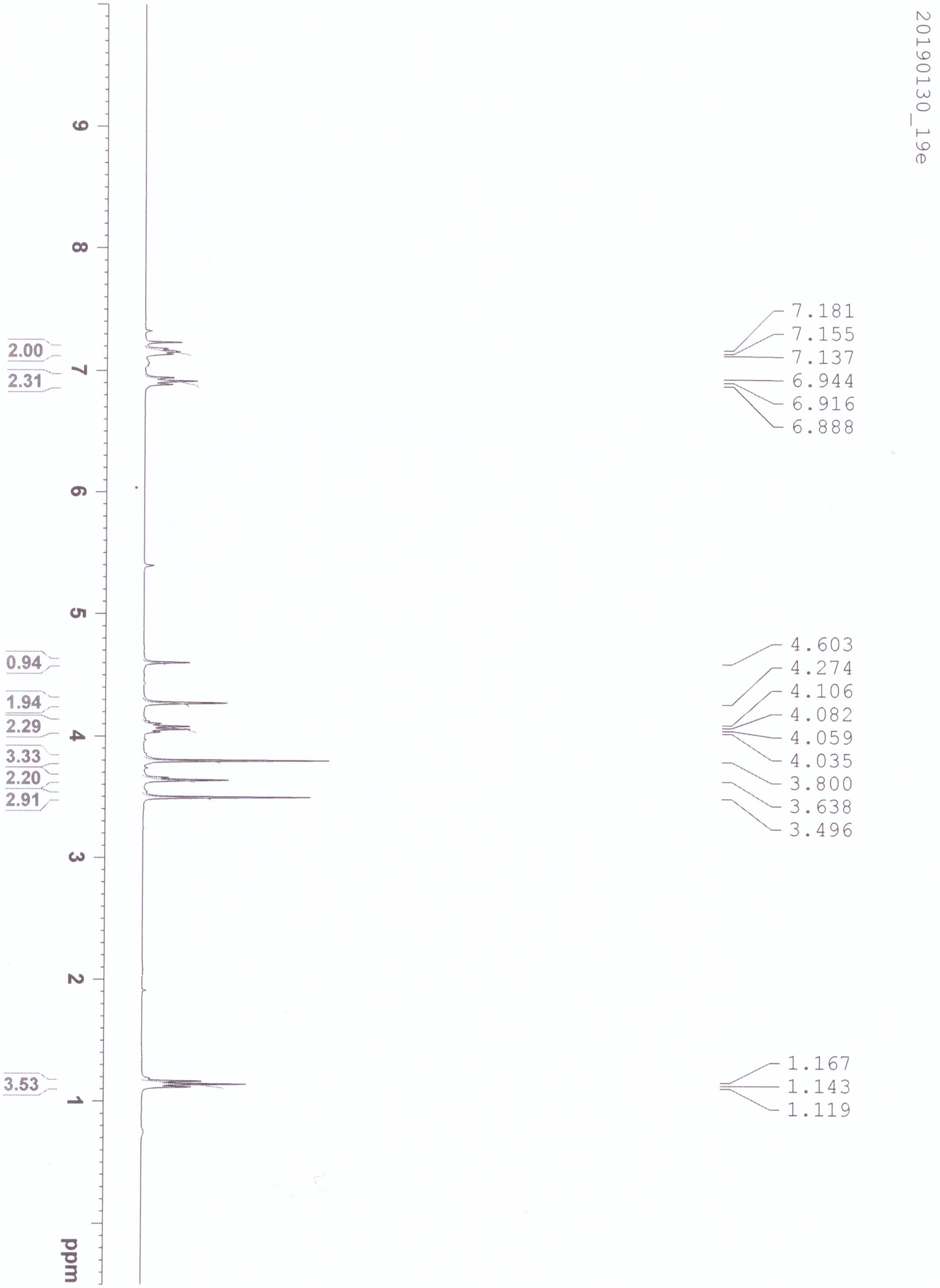

20190130_19e
7.181
7.155
7.137
6.944
6.916
6.888
4.603
4.274
4.106
4.082
4.059
4.035
3.800
3.638
3.496
1.167
1.143
1.119
2.00
2.31
0.94
1.94
2.29
3.33
2.20
2.91
3.53
ppm

19e

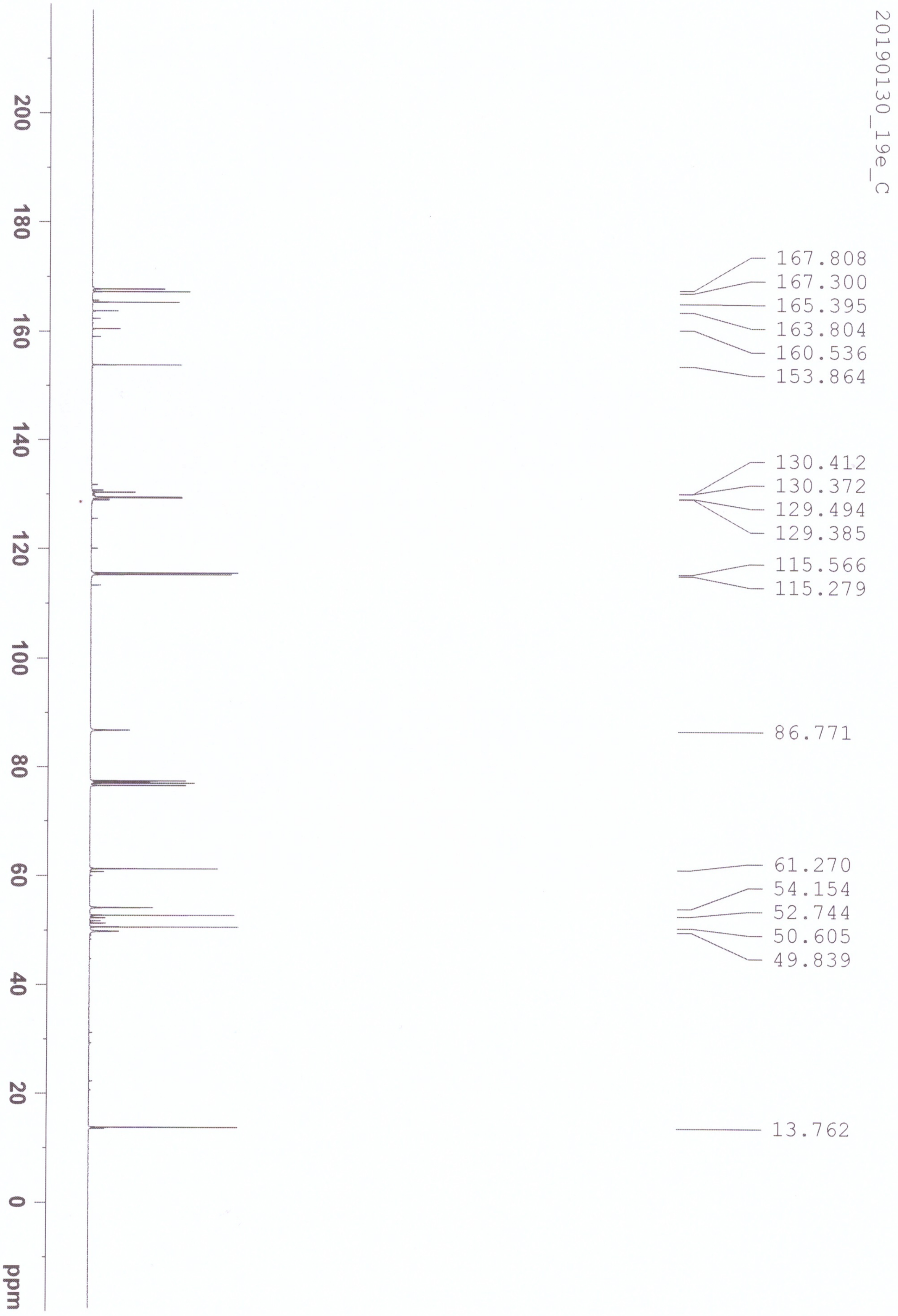
20190130_19e_C
167.808
167.300
165.395
163.804
160.536
153.864
130.412
130.372
129.494
129.385
115.566
115.279
86.771
61.270
54.154
52.744
50.605
49.839
13.762
200
180
160
140
120
100
80
60
40
20
0
ppm

18f

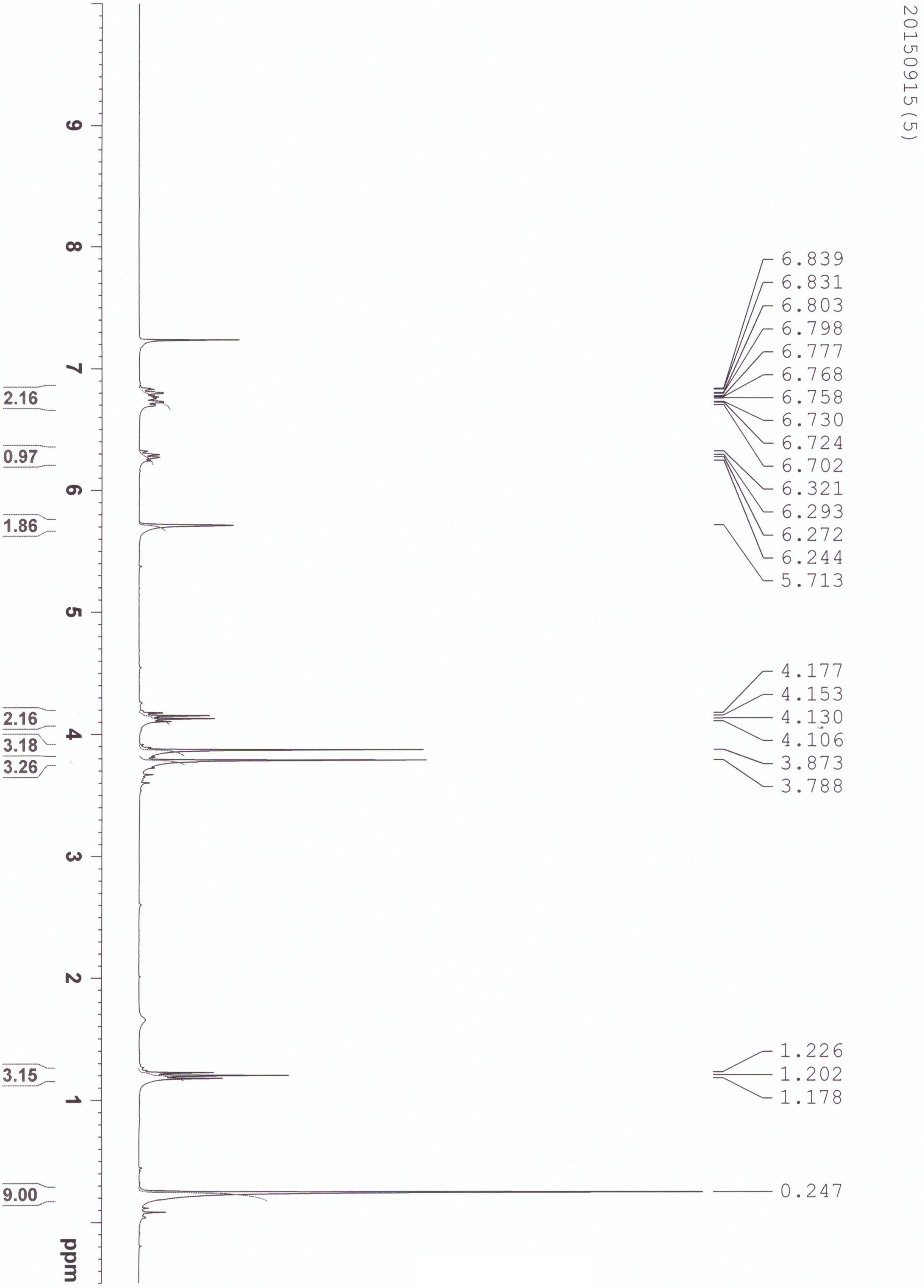
20150915(5)
6.839
6.831
6.803
6.798
6.777
6.768
6.758
6.730
6.724
6.702
6.321
6.293
6.272
6.244
5.713
4.177
4.153
4.130
4.106
3.873
3.788
1.226
1.202
1.178
0.247
2.16
0.97
1.86
2.16
3.18
3.26
3.15
9.00
ppm

18f

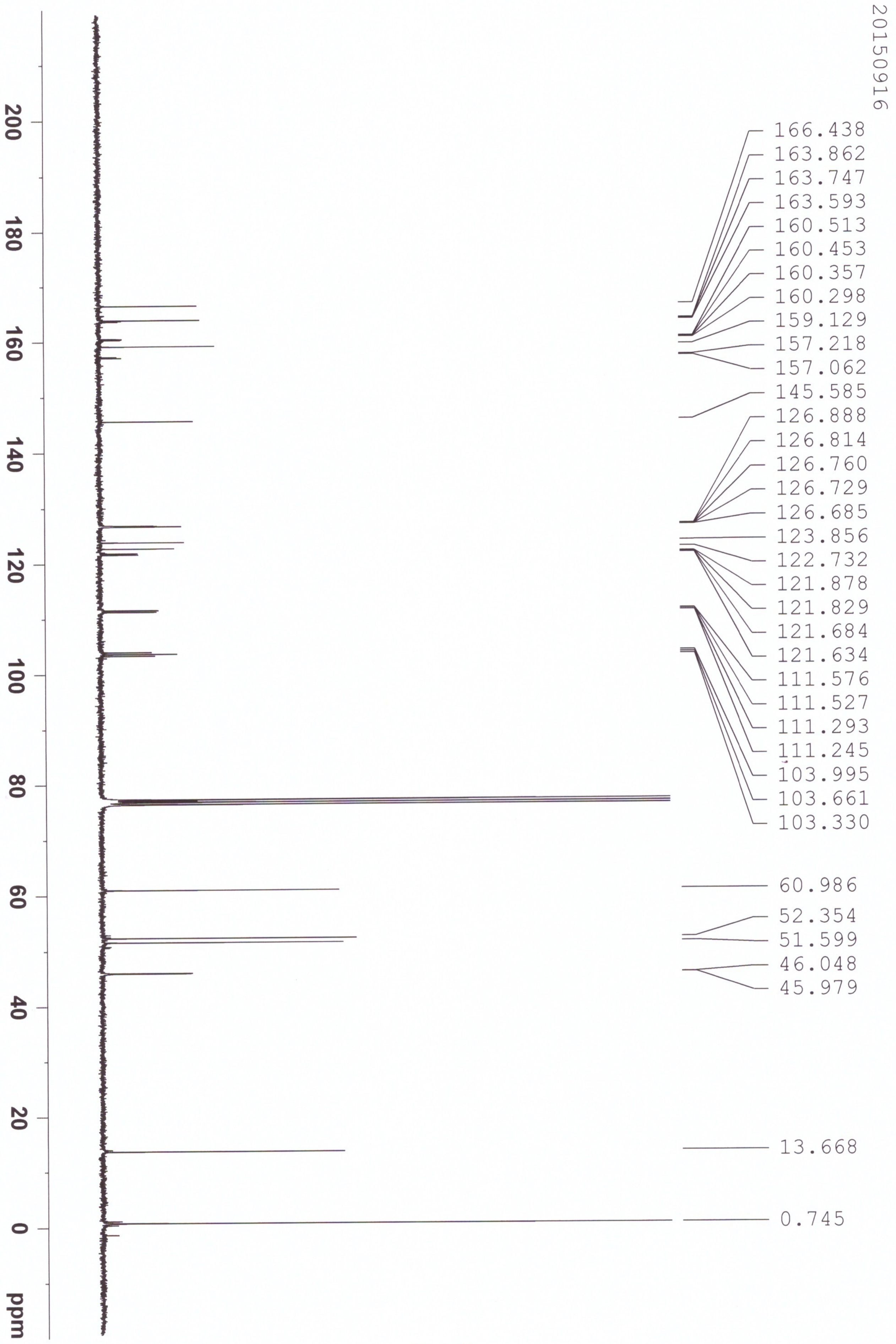
20150916
166.438
163.862
163.747
163.593
160.513
160.453
160.357
160.298
159.129
157.218
157.062
145.585
126.888
126.814
126.760
126.729
126.685
123.856
122.732
121.878
121.829
121.684
121.634
111.576
111.527
111.293
111.245
103.995
103.661
103.330
60.986
52.354
51.599
46.048
45.979
13.668
0.745
200
180
160
140
120
100
80
60
40
20
0
ppm

19f

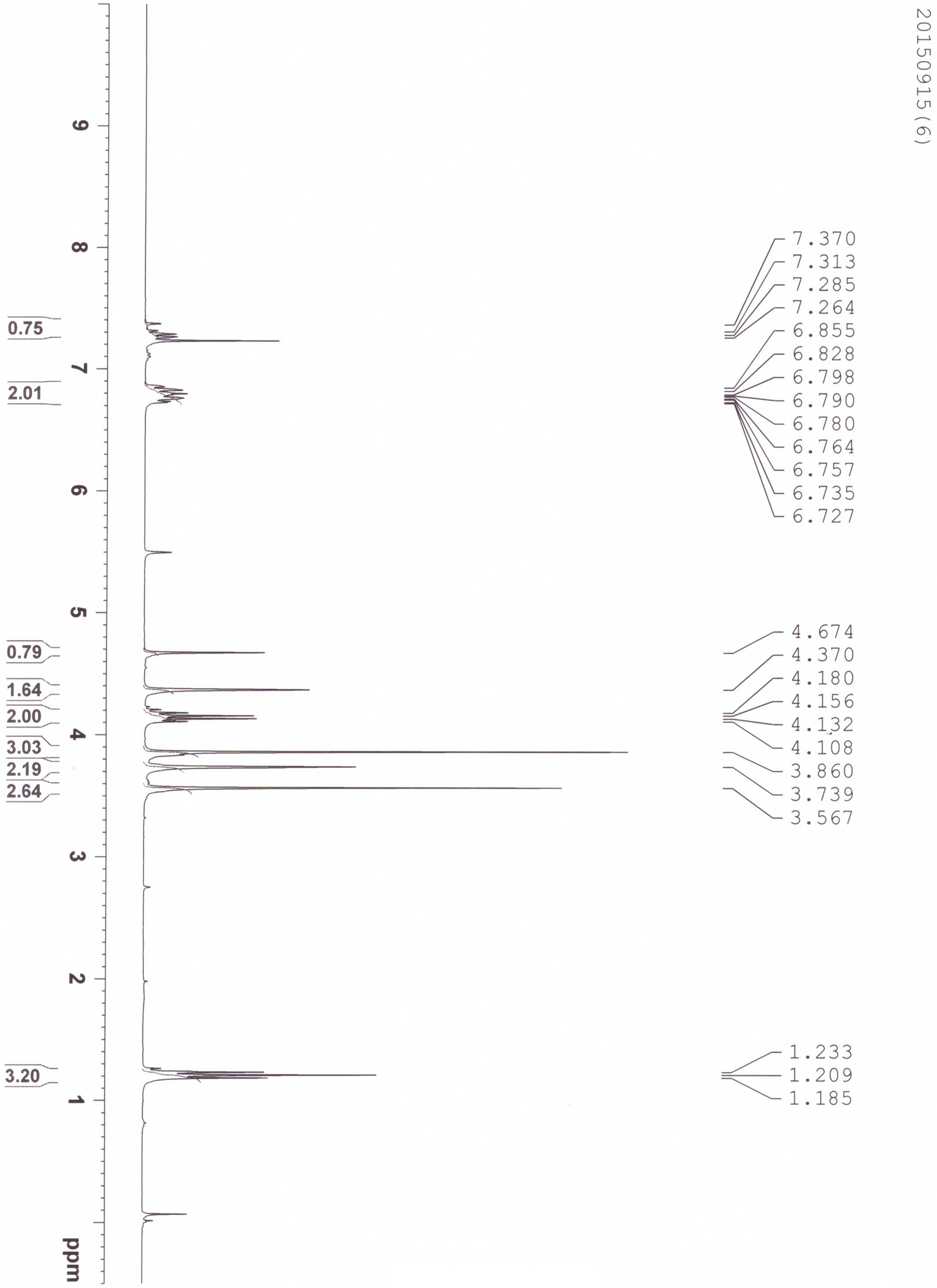
20150915(6)
7.370
7.313
7.285
7.264
6.855
6.828
6.798
6.790
6.780
6.764
6.757
6.735
6.727
4.674
4.370
4.180
4.156
4.132
4.108
3.860
3.739
3.567
1.233
1.209
1.185
0.75
2.01
0.79
1.64
2.00
3.03
2.19
2.64
3.20
9
8
7
6
5
4
3
2
1
ppm

19f

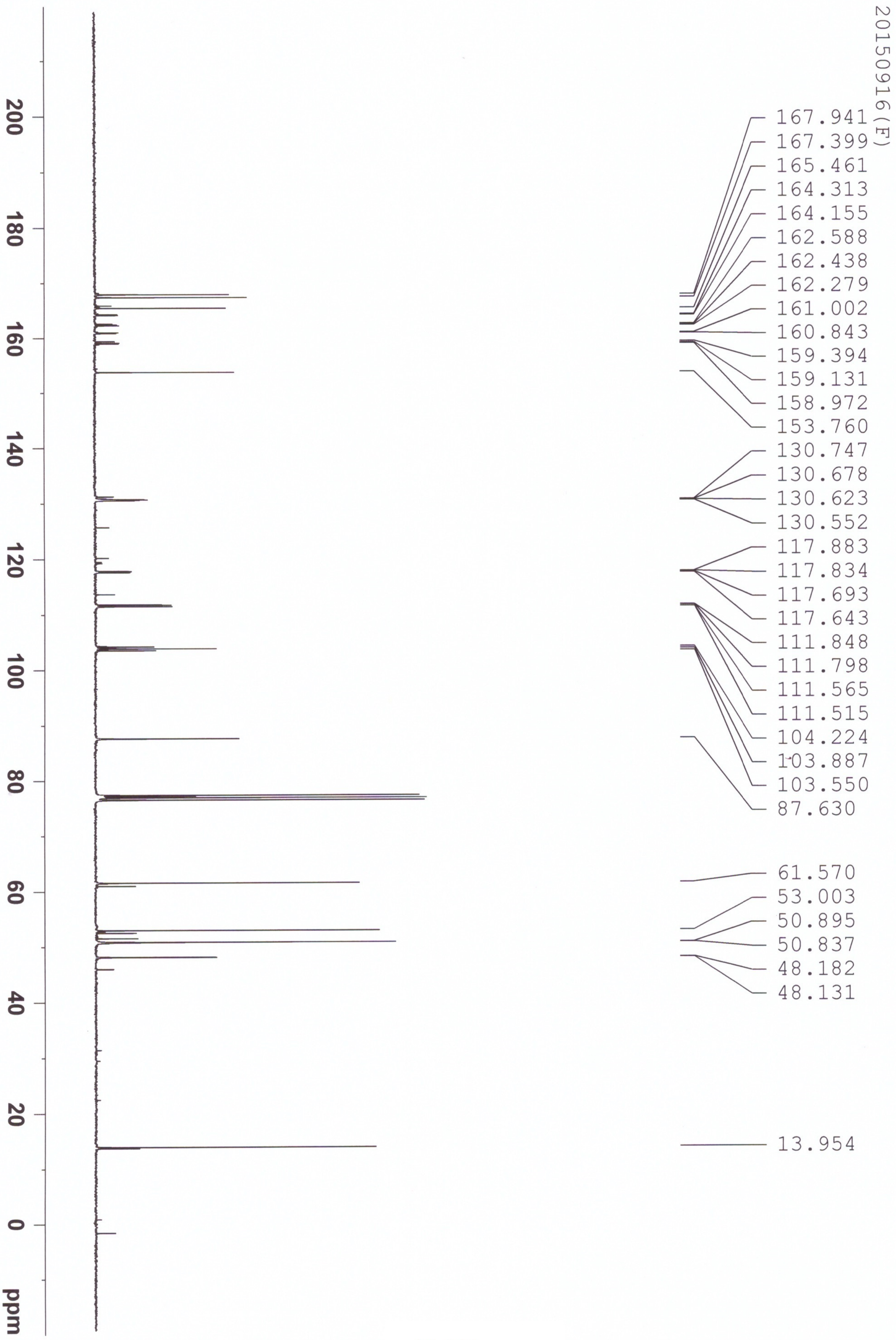
20150916(F)
167.941
167.399
165.461
164.313
164.155
162.588
162.438
162.279
161.002
160.843
159.394
159.131
158.972
153.760
130.747
130.678
130.623
130.552
117.883
117.834
117.693
117.643
111.848
111.798
111.565
111.515
104.224
103.887
103.550
87.630
61.570
53.003
50.895
50.837
48.182
48.131
13.954
200
180
160
140
120
100
80
60
40
20
0
ppm

18g

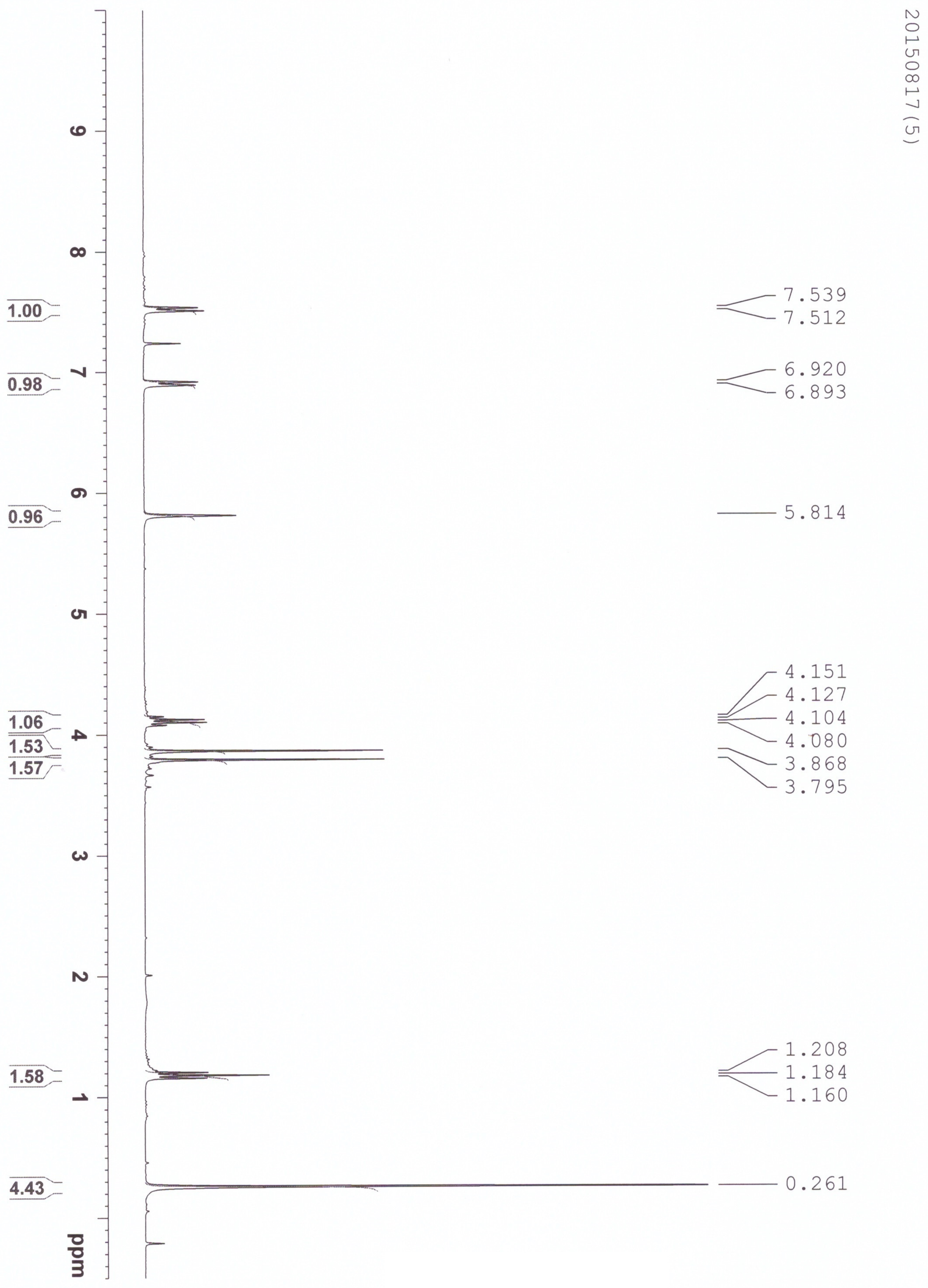

20150817(5)
7.539
7.512
6.920
6.893
5.814
4.151
4.127
4.104
4.080
3.868
3.795
1.208
1.184
1.160
0.261
1.00
0.98
0.96
1.06
1.53
1.57
1.58
4.43
9
8
7
6
5
4
3
2
1
ppm

18g

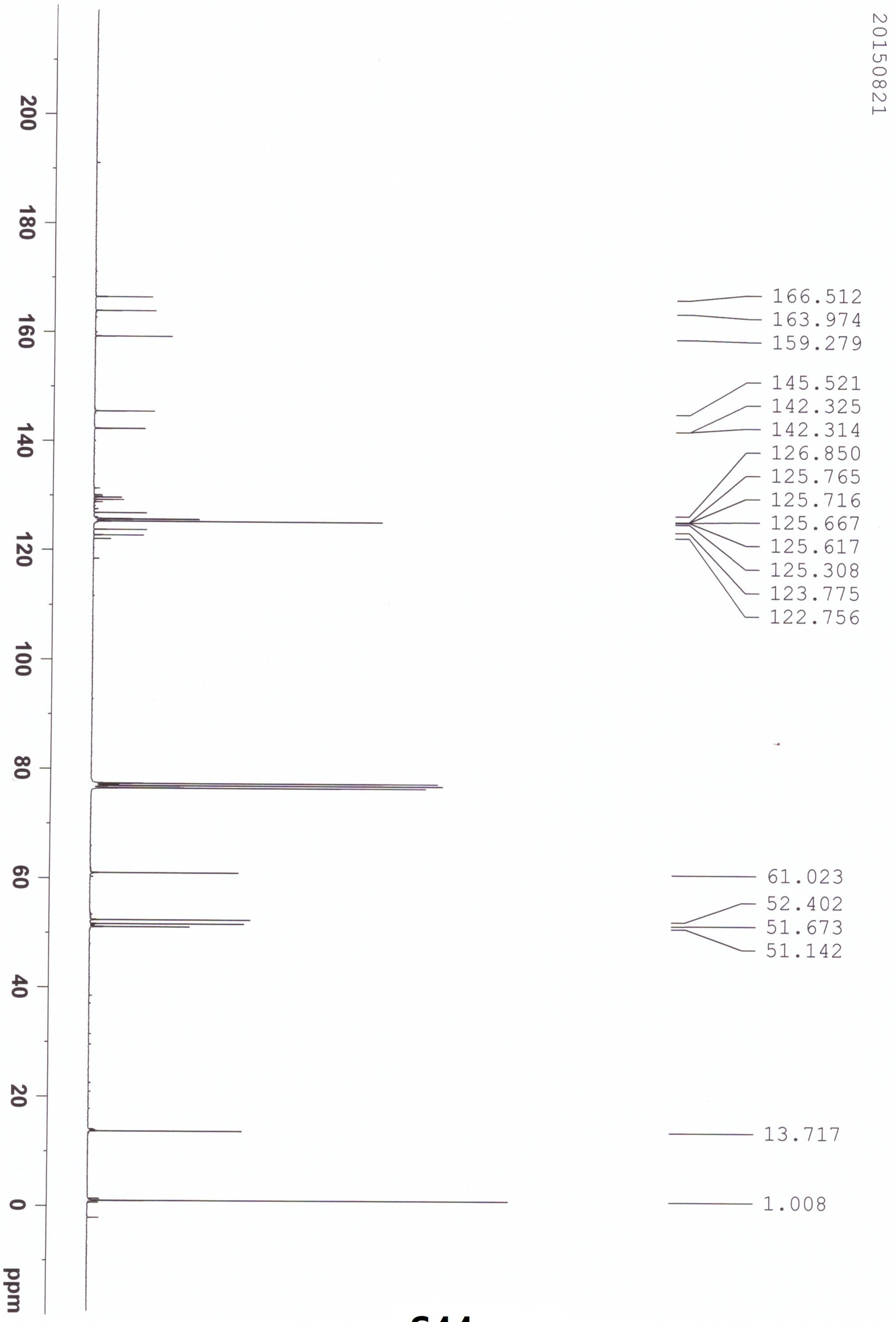
20150821
166.512
163.974
159.279
145.521
142.325
142.314
126.850
125.765
125.716
125.667
125.617
125.308
123.775
122.756
61.023
52.402
51.673
51.142
13.717
1.008
200
180
160
140
120
100
80
60
40
20
0
ppm

19g

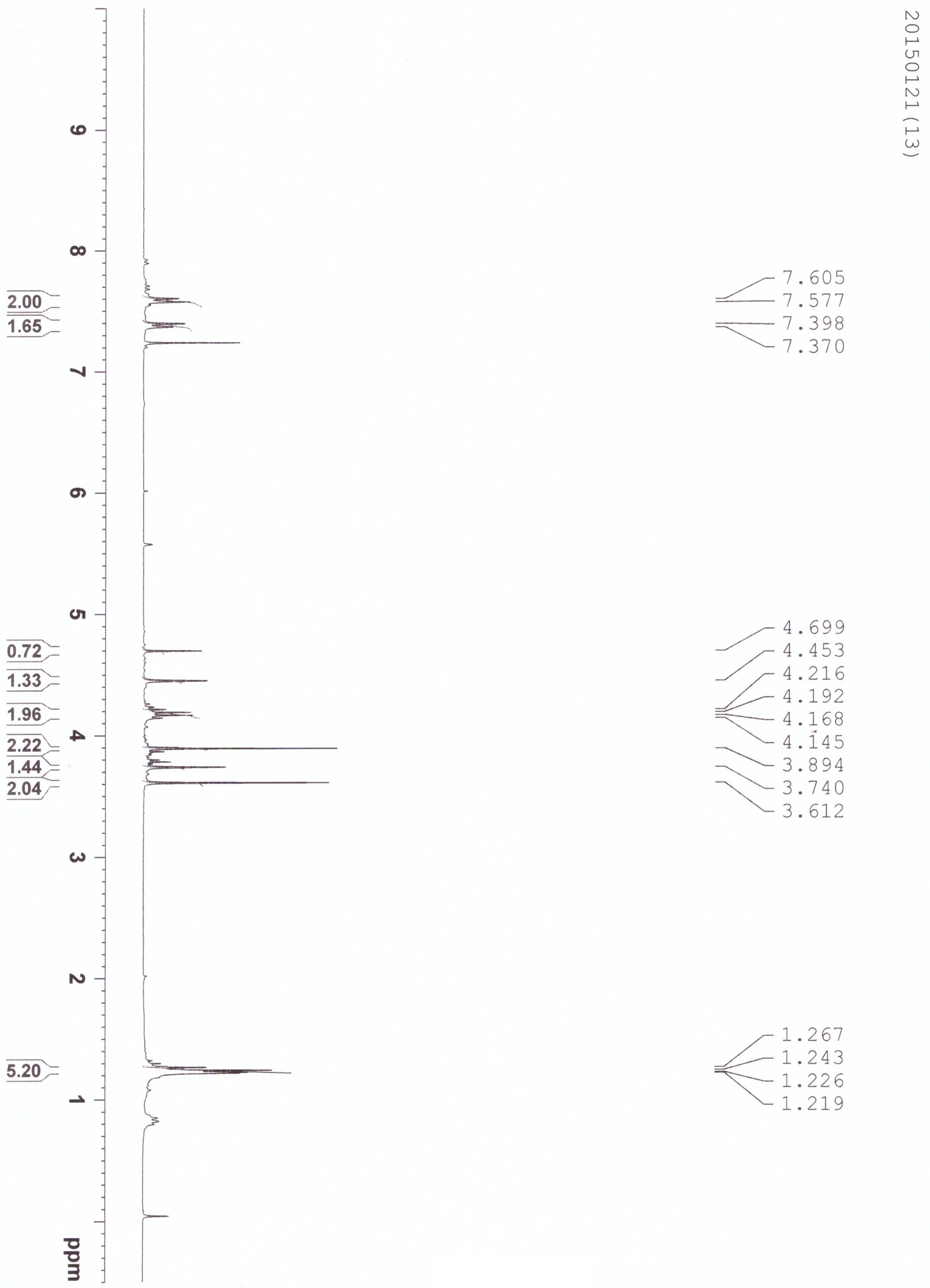
20150121(13)
7.605
7.577
7.398
7.370
4.699
4.453
4.216
4.192
4.168
4.145
3.894
3.740
3.612
1.267
1.243
1.226
1.219
2.00
1.65
0.72
1.33
1.96
2.22
1.44
2.04
5.20
ppm

19g

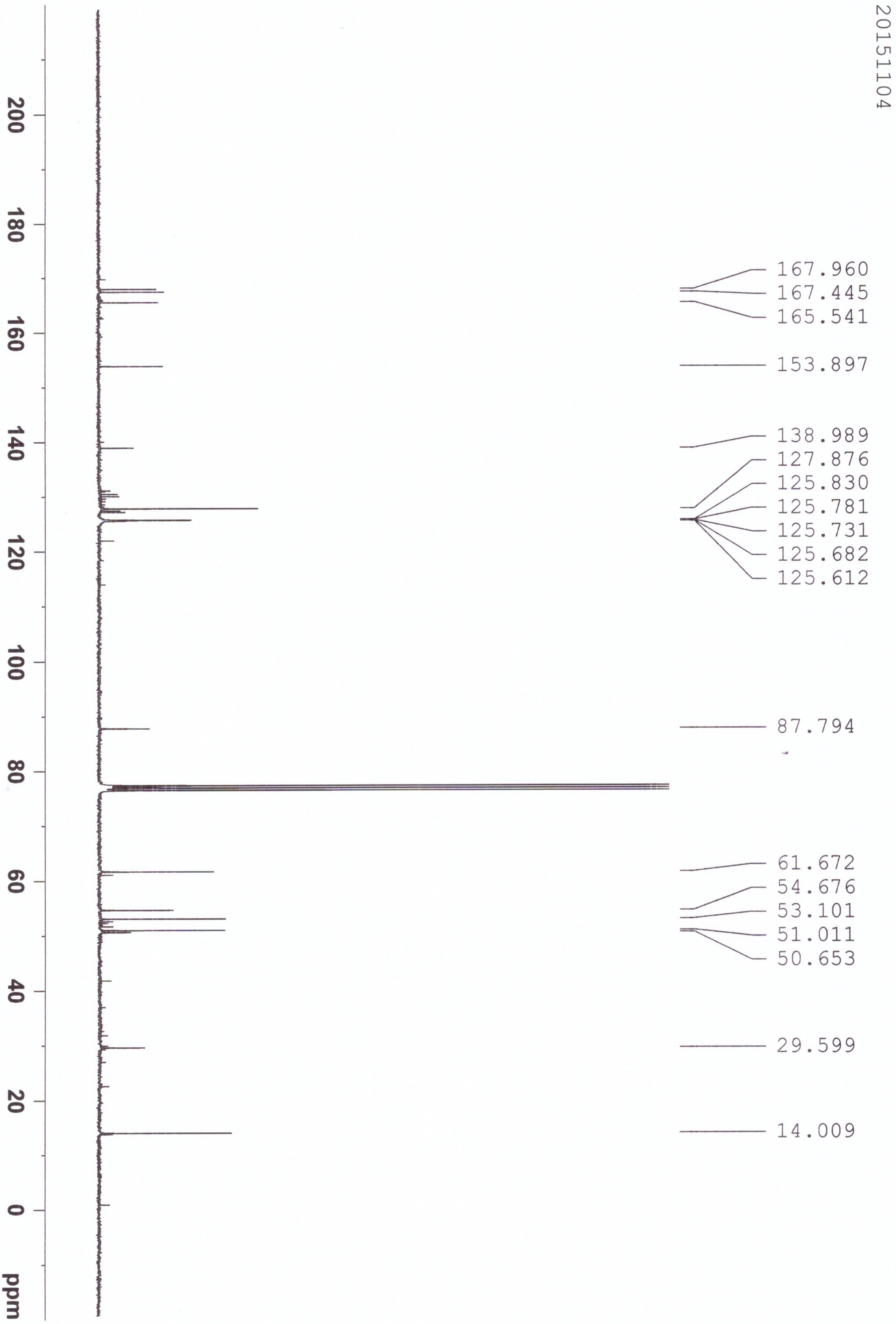
20151104
167.960
167.445
165.541
153.897
138.989
127.876
125.830
125.781
125.731
125.682
125.612
87.794
61.672
54.676
53.101
51.011
50.653
29.599
14.009
ppm

22

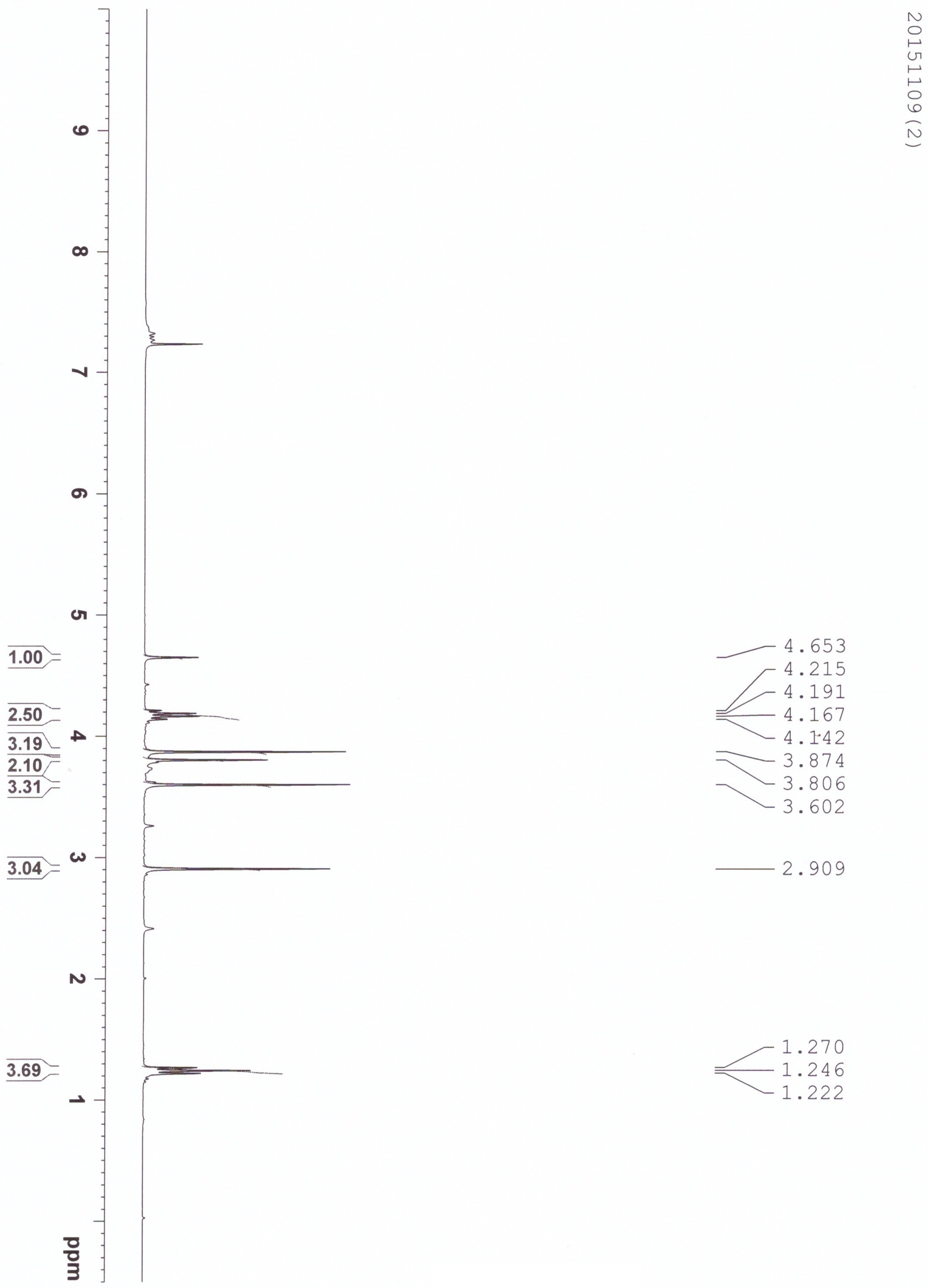
20151109(2)
4.653
4.215
4.191
4.167
4.142
3.874
3.806
3.602
2.909
1.270
1.246
1.222
1.00
2.50
3.19
2.10
3.31
3.04
3.69
9
8
7
6
5
4
3
2
1
ppm

22

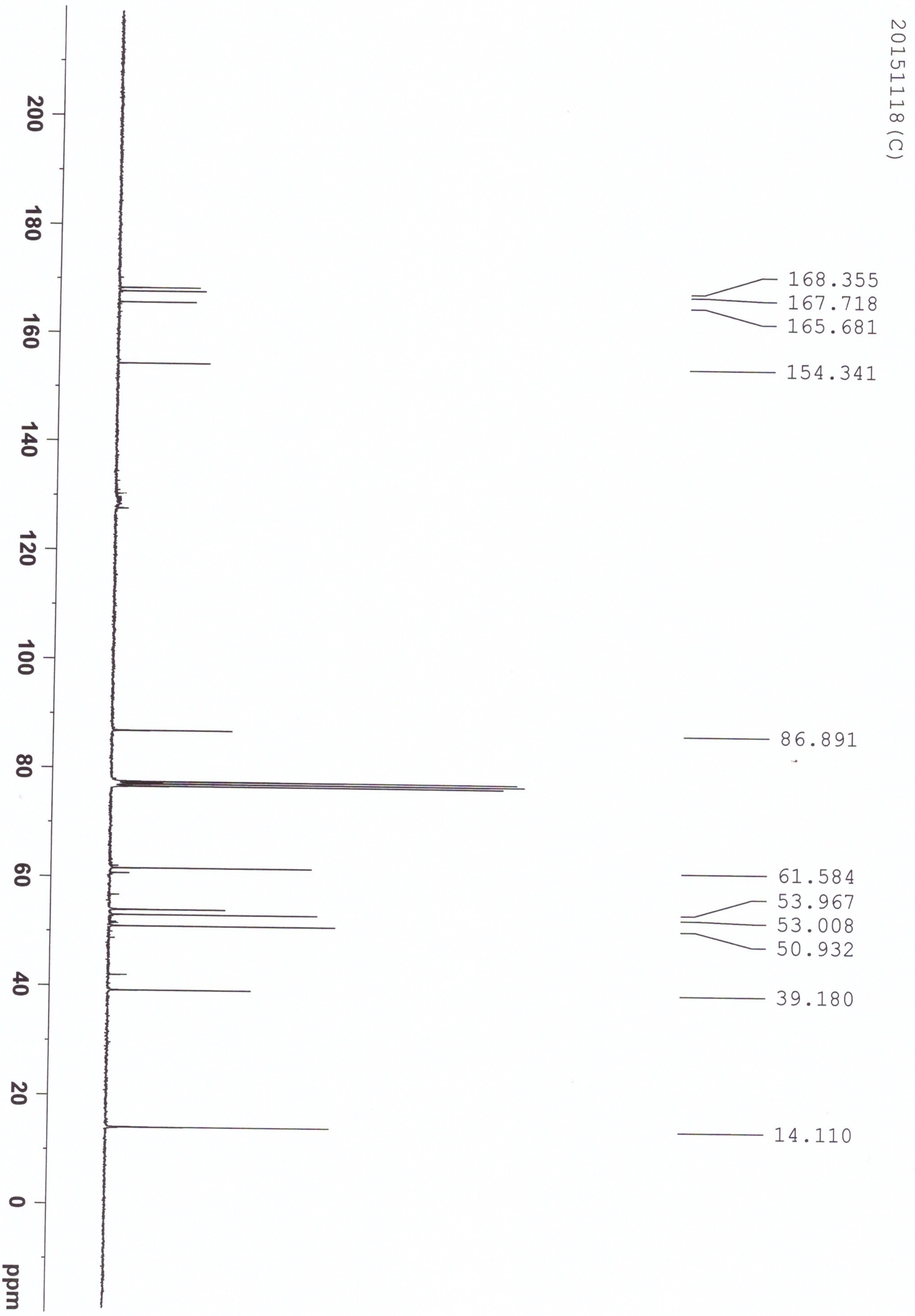
20151118(C)
168.355
167.718
165.681
154.341
86.891
61.584
53.967
53.008
50.932
39.180
14.110
200
180
160
140
120
100
80
60
40
20
0
ppm

23

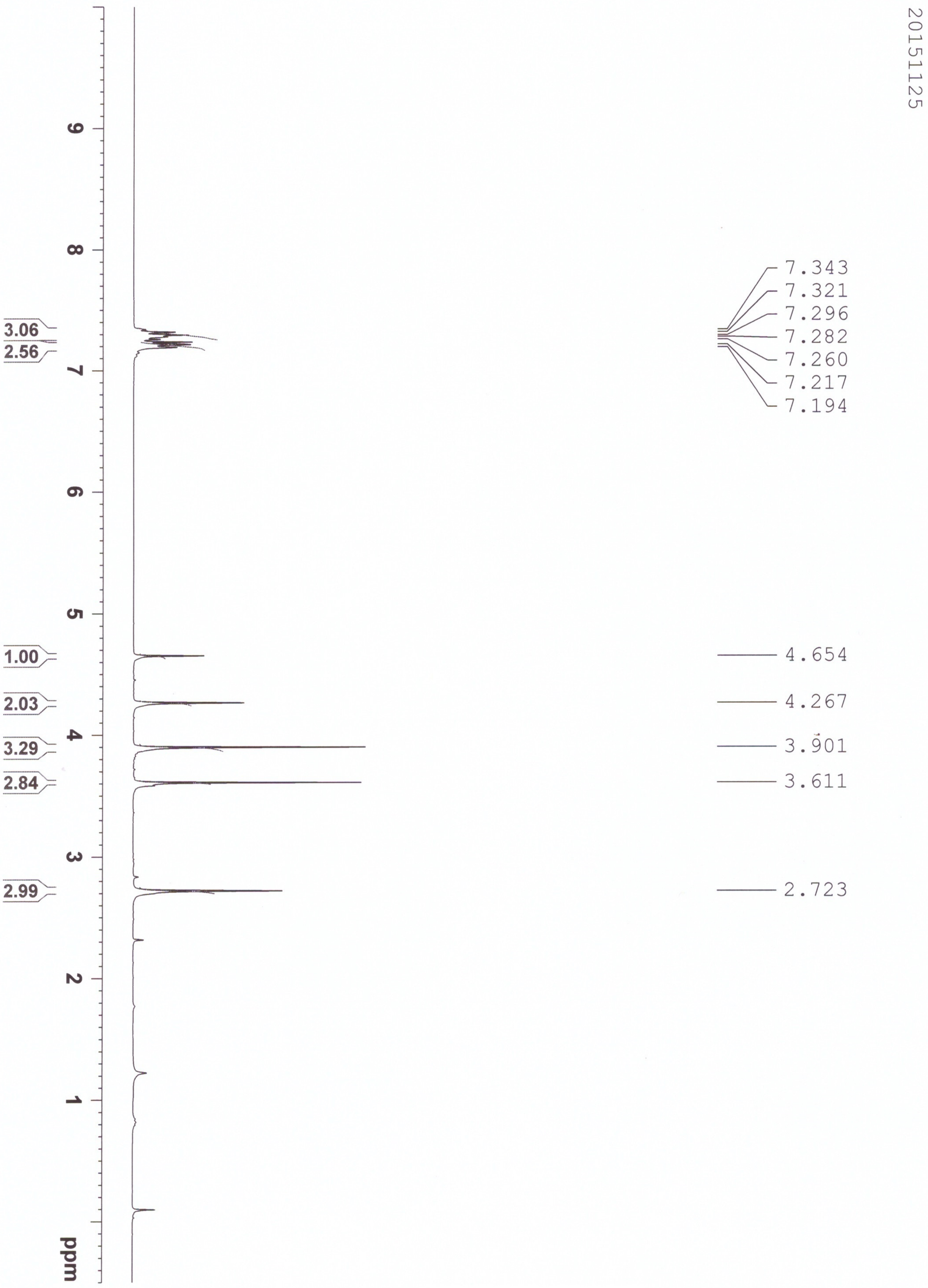
20151125
7.343
7.321
7.296
7.282
7.260
7.217
7.194
4.654
4.267
3.901
3.611
2.723
3.06
2.56
1.00
2.03
3.29
2.84
2.99
ppm

23

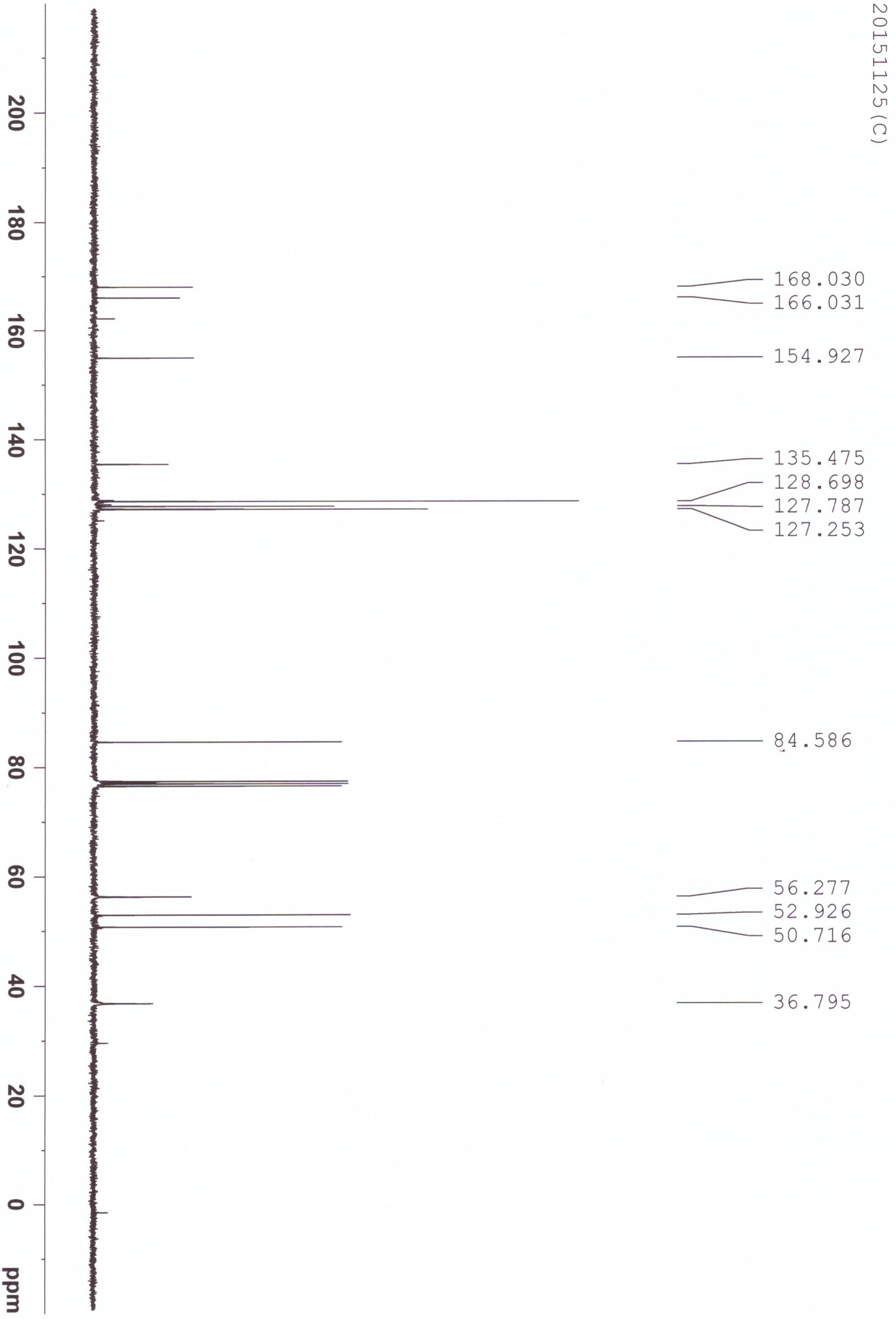
20151125(C)
168.030
166.031
154.927
135.475
128.698
127.787
127.253
84.586
56.277
52.926
50.716
36.795
200
180
160
140
120
100
80
60
40
20
0
ppm
